# Supplementary material for: Phytosterol, Lipid and Phenolic Composition, and Biological Activities of Guava Seed Oil
Source: Molecules. 2020 May 27;25(11):2474. doi: 10.3390/molecules25112474 (PMC7321134; doi:10.3390/molecules25112474)
Supplement: Supplementary file 1 [file molecules-25-02474-s001.zip › molecules-804750-final-SM/Figure S1 HPLC ESI QTOF MS for lipids of GSO.pdf]

Target Compound Screening Report

**Data File**	guavaoil.d	**Sample Name**	guava seed oil
**Sample Type**	Sample	**Position**	P1-A2
**Instrument Name**	Instrument 1	**User Name**	
**Acq Method**	Pesticides screening_20170825.m	**Acquired Time**	3/13/2020 3:03:59 PM (UTC+07:00)
**IRM Calibration Status**	All Ions Missed	**DA Method**	Pesticides_Screening_MFE.m
**Comment**			
**Sample Group**		**Info.**	
**Stream Name**	LC 1	**Acquisition Time (Local)**	3/13/2020 3:03:59 PM (UTC+07:00)
**Acquisition SW Version**	6200 series TOF/6500 series Q-TOF B.08.00 (B8058.0)	**QTOF Driver Version**	8.00.00
**QTOF Firmware Version**	25.698	**Tune Mass Range Max.**	3200

Compound Table

Label	Tgt Name	Tgt Score	Mass Error (ppm)	Tgt Formula	Obs. RT	Ref. Mass	Obs. Mass
Cpd 1: 4-hexyl-decanoic acid; C16 H32 O2; 0.576	4-hexyl-decanoic acid	96.14	3.86	C16 H32 O2	0.576	256.2402	256.2412
Cpd 7: C16 Sphinganine; C16 H35 N O2; 0.576	C16 Sphinganine	96.14	3.54	C16 H35 N O2	0.576	273.2668	273.2677
Cpd 2: 5S-HETE di-endoperoxide; C20 H34 O8; 1.051	5S-HETE di-endoperoxide	82.26	1.82	C20 H34 O8	1.051	402.2254	402.2261
Cpd 5: Didrovaltratum; C22 H32 O8; 1.051	Didrovaltratum	89.23	-3.36	C22 H32 O8	1.051	424.2097	424.2083
Cpd 9: Sphingofungin B; C20 H39 N O6; 1.066	Sphingofungin B	95.18	1.45	C20 H39 N O6	1.066	389.2777	389.2783
Cpd 3: 13,14-dihydro-19(R)-hydroxyPGE1; C20 H36 O6; 1.094	13,14-dihydro-19(R)-hydroxyPGE1	96.85	2.19	C20 H36 O6	1.094	372.2512	372.252
Cpd 6: Eschscholtzxanthin; C40 H54 O2; 1.177	Eschscholtzxanthin	52.09	-1.18	C40 H54 O2	1.177	566.4124	566.4117
Cpd 4: Tetradecan-3-one; C14 H28 O; 21.926	Tetradecan-3-one	98.82	2.03	C14 H28 O	21.926	212.214	212.2144
Cpd 8: Xestoaminol C; C14 H31 N O; 21.926	Xestoaminol C	98.82	1.88	C14 H31 N O	21.926	229.2406	229.241

Name	Obs. *m/z*	Obs. RT	Obs. Mass	Tgt Formula	Tgt Mass	Tgt Mass Error (ppm)	Find Cpds Algorithm
4-hexyl-decanoic acid	274.2751	0.576	256.2412	C16 H32 O2	256.2402	3.86	Find by Formula

Compound Chromatograms

Target Compound Screening Report

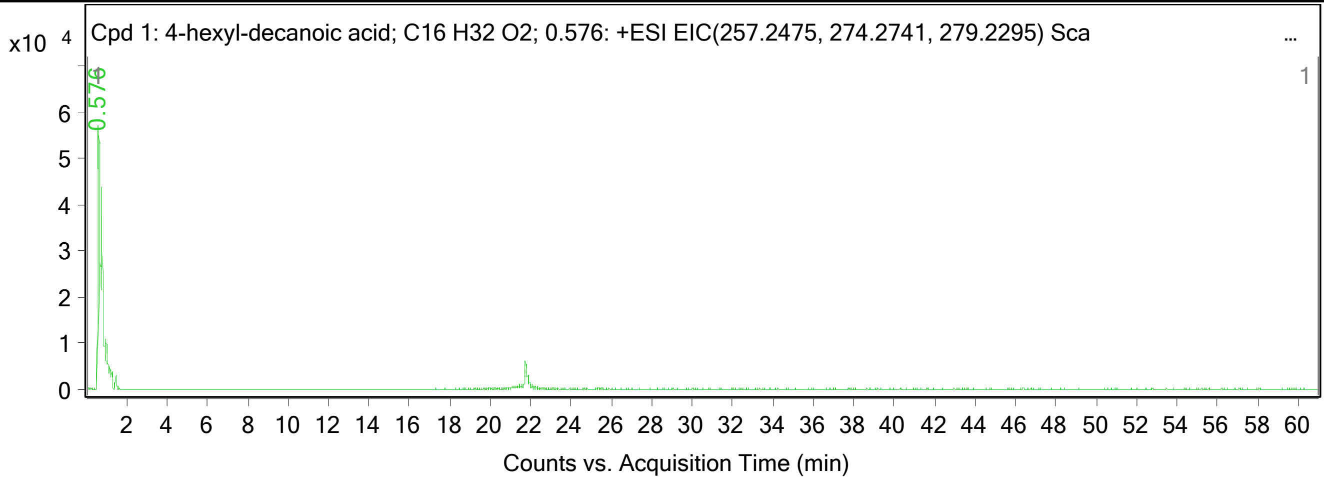

MS Zoomed Spectrum

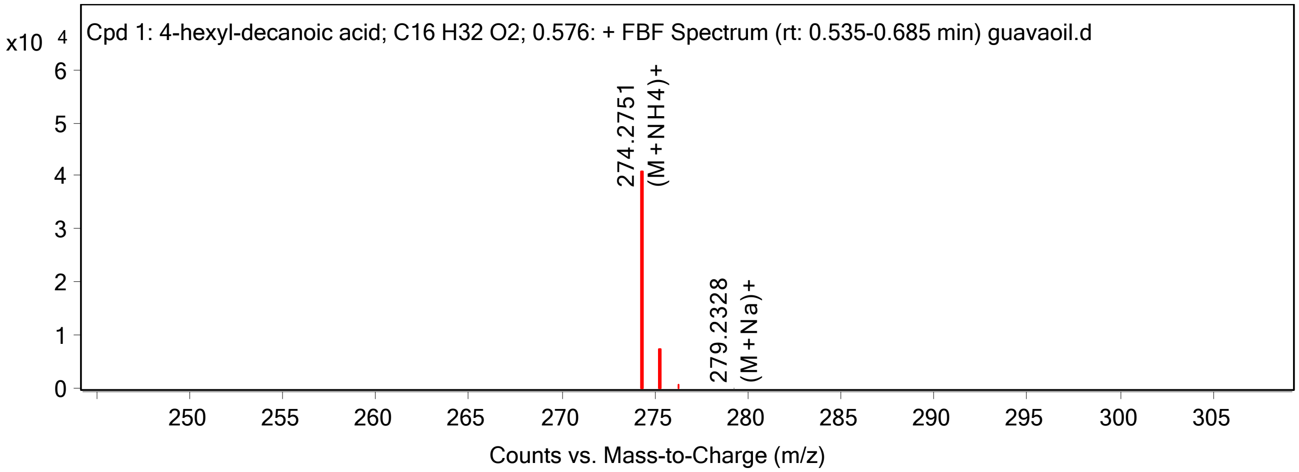

MS Spectrum Peak List

Obs. *m/z*	Charge	Abund	Ion/Isotope
274.2751	1	40796.12	(M+NH4)+
275.2782	1	6979.9	(M+NH4)+
276.2812	1	707.75	(M+NH4)+
279.2328	1	153.3	(M+Na)+

MS Zoomed Spectrum

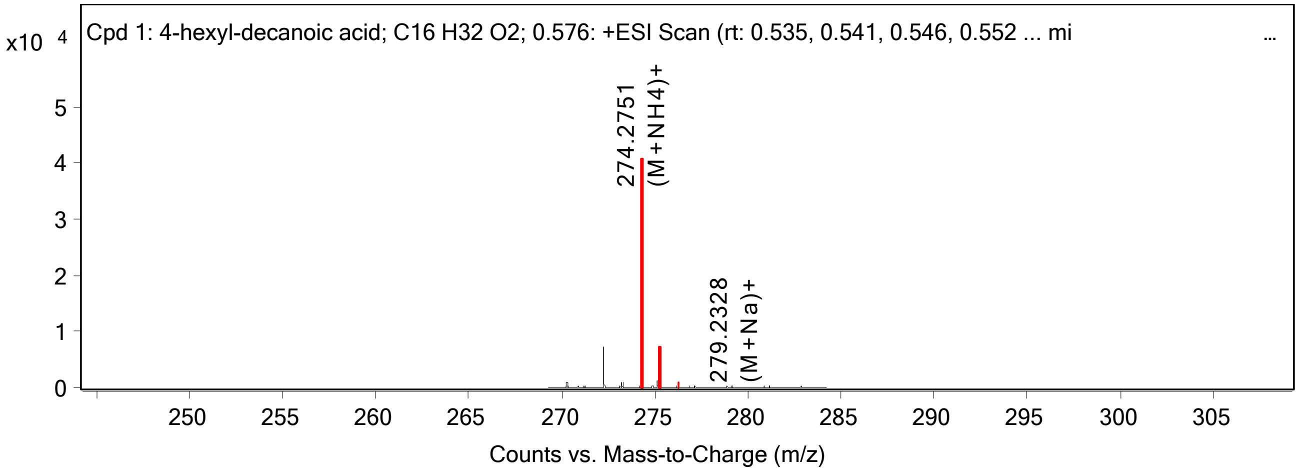

MS Spectrum Peak List

Obs. *m/z*	Charge	Abund	Ion/Isotope	Tgt Mass Error (ppm)

274.2751		40791.44		-3.67
274.2751	1	40796.12	(M+NH4)+	-3.67
275.2782	1	6979.9	(M+NH4)+	-2.99
276.2812	1	707.75	(M+NH4)+	-3.85
279.2328	1	153.3	(M+Na)+	-11.86

Name	Obs. *m/z*	Obs. RT	Obs. Mass	Tgt Formula	Tgt Mass	Tgt Mass Error (ppm)	Find Cpds Algorithm
C16 Sphinganine	274.2751	0.576	273.2677	C16 H35 N O2	273.2668	3.54	Find by Formula

Compound Chromatograms

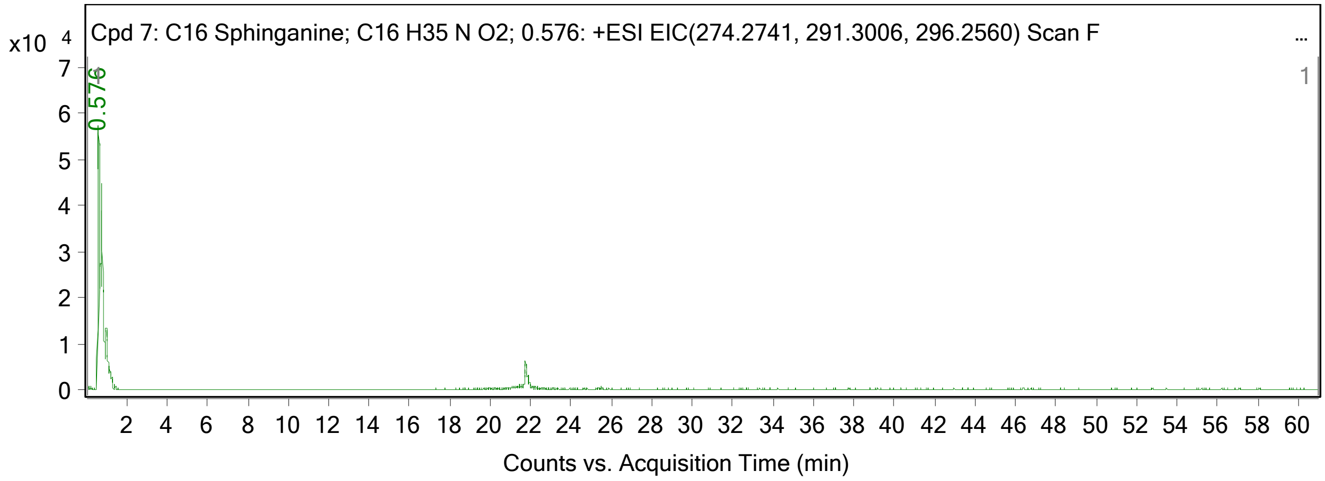

MS Zoomed Spectrum

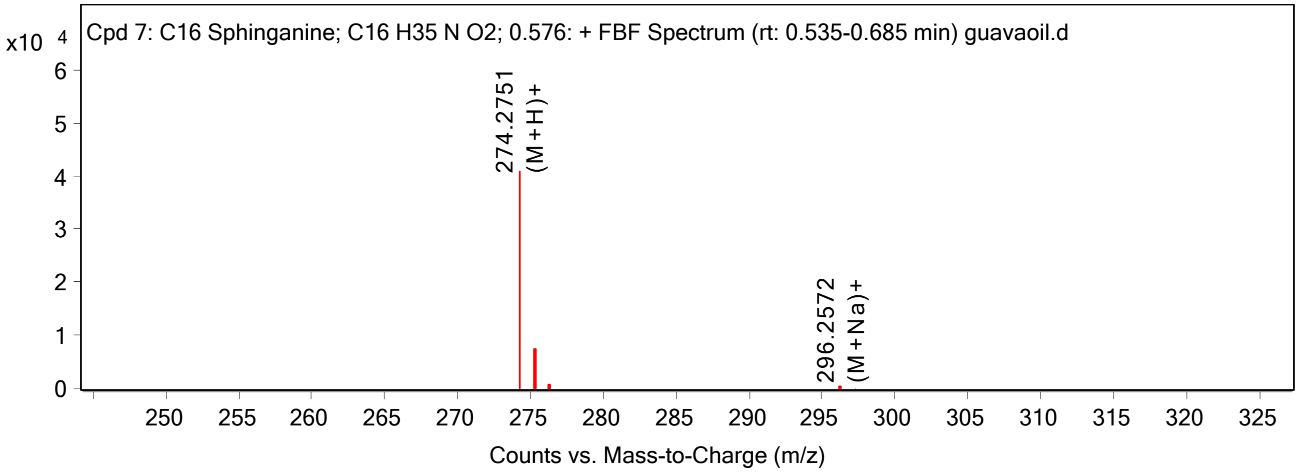

MS Spectrum Peak List

Obs. *m/z*	Charge	Abund	Ion/Isotope
274.2751	1	40796.12	(M+H)+
275.2782	1	6979.9	(M+H)+
276.2812	1	707.75	(M+H)+
296.2572	1	325.45	(M+Na)+
297.249	1	70.74	(M+Na)+

MS Zoomed Spectrum

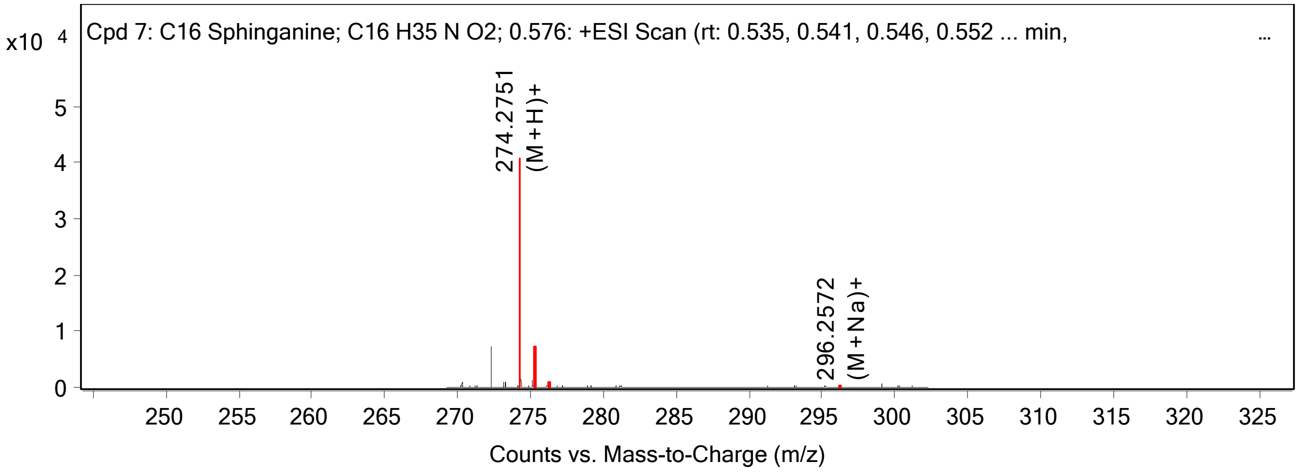

MS Spectrum Peak List

Obs. *m/z*	Charge	Abund	Ion/Isotope	Tgt Mass Error (ppm)
274.2751		40791.44		
274.2751	1	40796.12	(M+H)+	-3.67
275.2782	1	6979.9	(M+H)+	-2.99
276.2812	1	707.75	(M+H)+	-3.85
296.2572	1	325.45	(M+Na)+	-4.05
297.249	1	70.74	(M+Na)+	34.63

Name	Obs. *m/z*	Obs. RT	Obs. Mass	Tgt Formula	Tgt Mass	Tgt Mass Error (ppm)	Find Cpds Algorithm
5S-HETE di-endoperoxide	425.2153	1.051	402.2261	C20 H34 O8	402.2254	1.82	Find by Formula

Compound Chromatograms

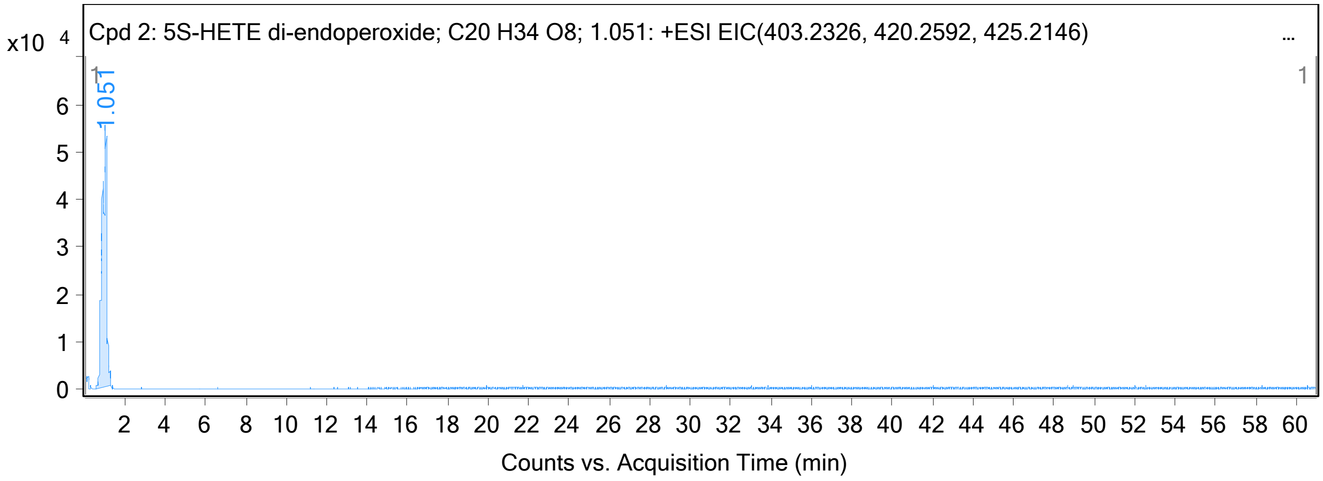

MS Zoomed Spectrum

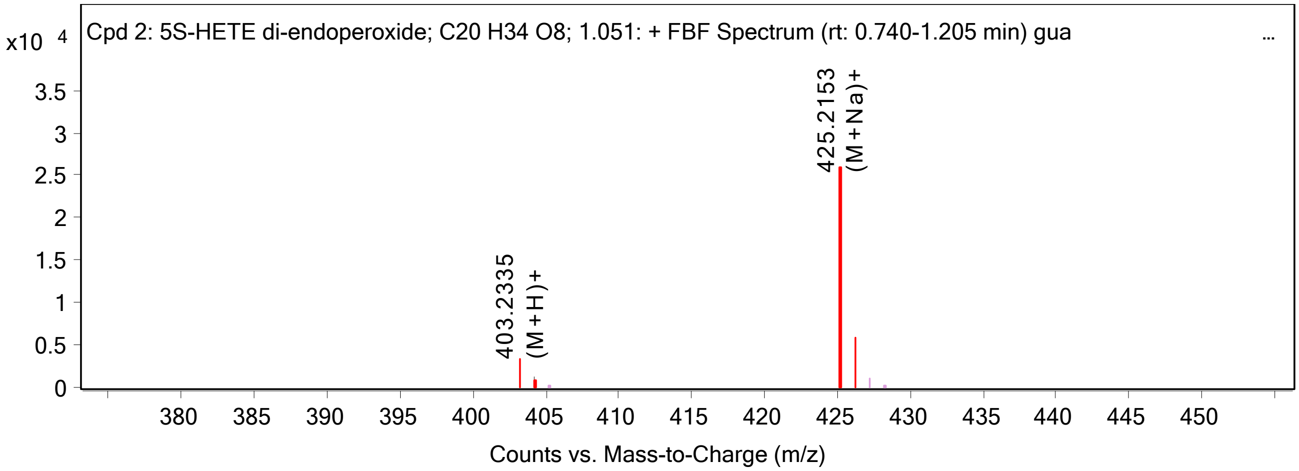

MS Spectrum Peak List

Obs. *m/z*	Charge	Abund	Ion/Isotope
403.2335	1	2864.07	(M+H)+
404.2393	1	1186.12	(M+H)+
425.2153	1	25849.69	(M+Na)+
426.2184	1	5663.76	(M+Na)+

MS Zoomed Spectrum

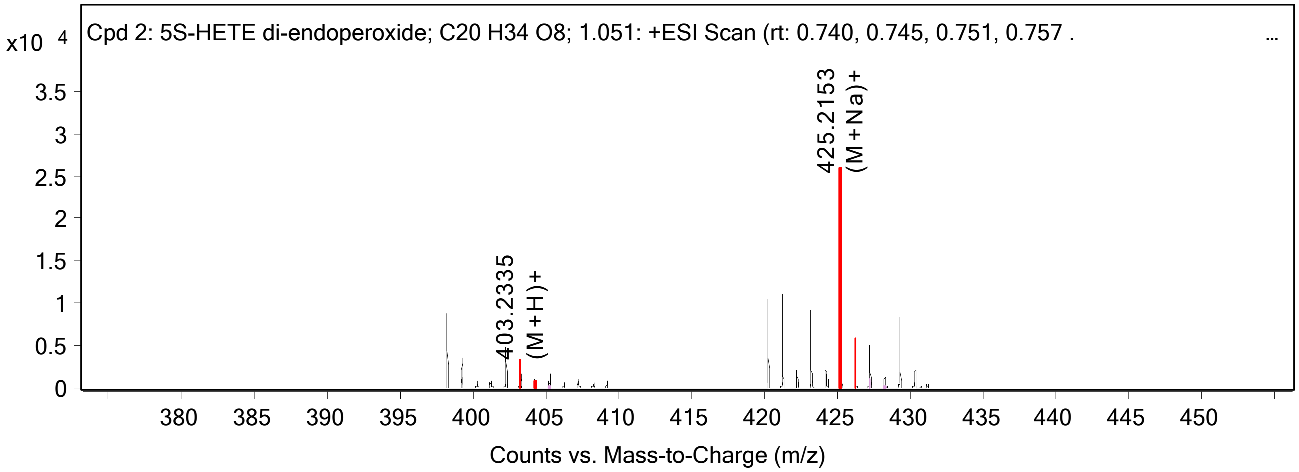

MS Spectrum Peak List

Obs. *m/z*	Charge	Abund	Ion/Isotope	Tgt Mass Error (ppm)
403.2335	1	2864.07	(M+H)+	-2.03
404.2393	1	1186.12	(M+H)+	-8.01
425.2153	1	25849.69	(M+Na)+	-1.62
425.2153		25841.95		
426.2184	1	5663.76	(M+Na)+	-0.81

Name	Obs. *m/z*	Obs. RT	Obs. Mass	Tgt Formula	Tgt Mass	Tgt Mass Error (ppm)	Find Cpds Algorithm
Didrovaltratum	425.2156	1.051	424.2083	C22 H32 O8	424.2097	-3.36	Find by Formula

Compound Chromatograms

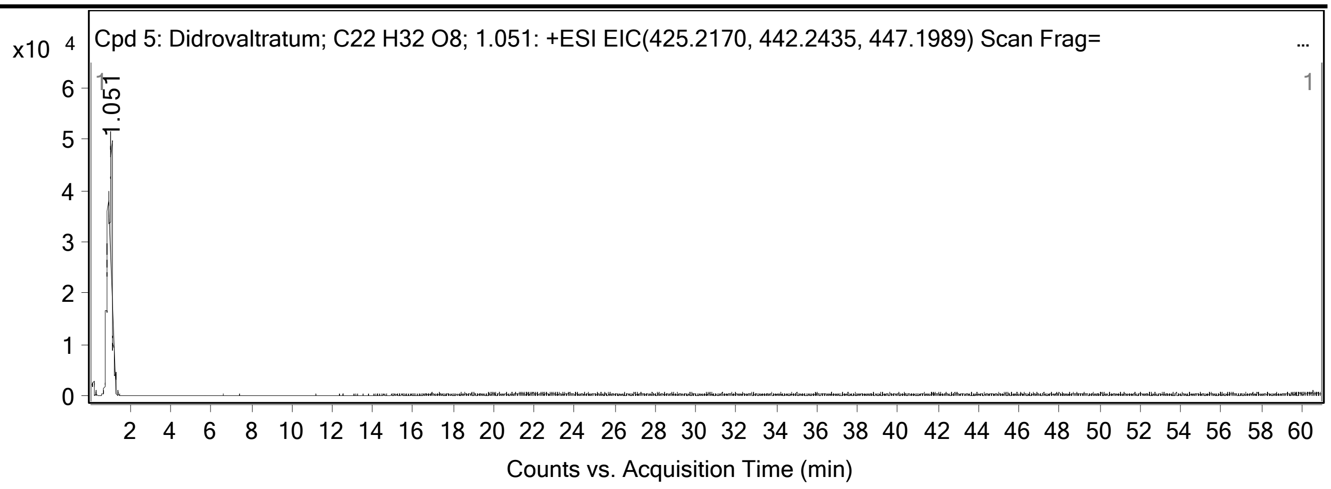

MS Zoomed Spectrum

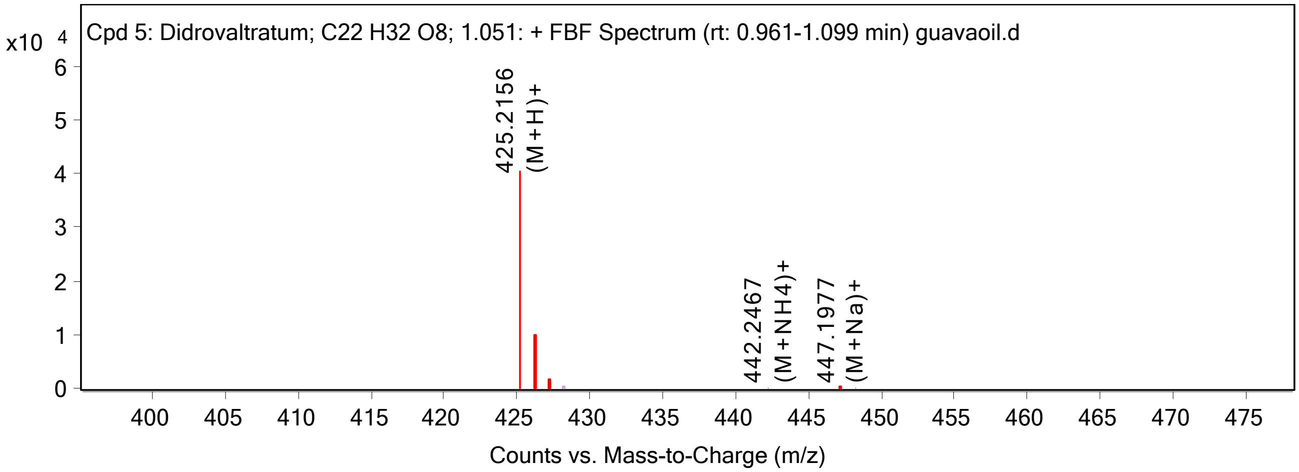

MS Spectrum Peak List

Obs. *m/z*	Charge	Abund	Ion/Isotope
425.2156	1	40296.68	(M+H)+
426.2187	1	8606.83	(M+H)+
427.2216	1	91.42	(M+H)+
442.2467	1	92.27	(M+NH4)+
447.1977	1	226.13	(M+Na)+
448.2011	1	48.77	(M+Na)+

MS Zoomed Spectrum

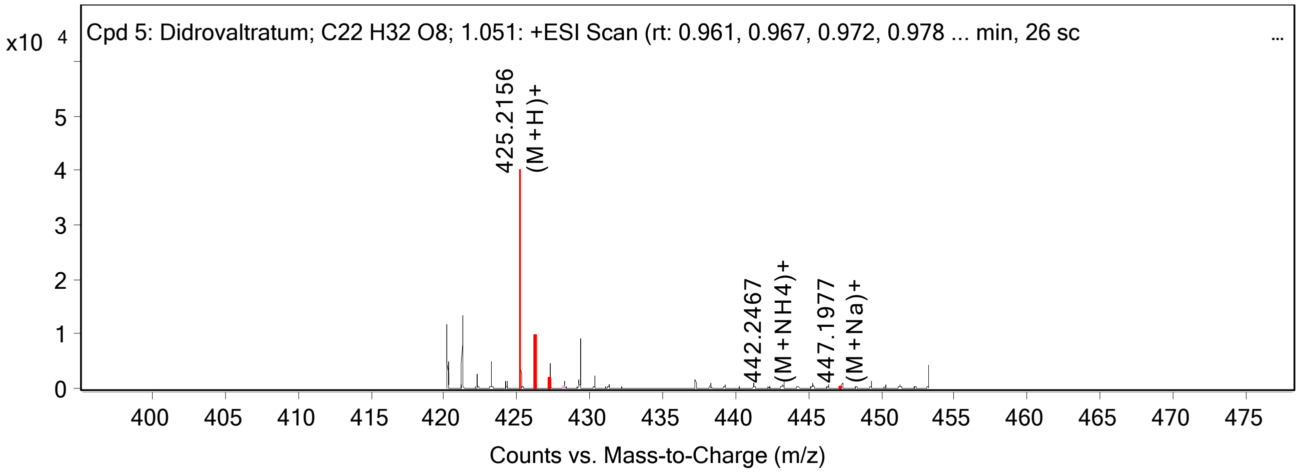

MS Spectrum Peak List

Obs. m/z	Charge	Abund	Ion/Isotope	Tgt Mass Error (ppm)
425.2156	1	40296.68	(M+H)+	3.24
425.2156		40290.14		
426.2187	1	8606.83	(M+H)+	3.99
427.2216	1	91.42	(M+H)+	2.93
442.2467	1	92.27	(M+NH4)+	-7.22
447.1977	1	226.13	(M+Na)+	2.82
448.2011	1	48.77	(M+Na)+	2.79

Name	Obs. m/z	Obs. RT	Obs. Mass	Tgt Formula	Tgt Mass	Tgt Mass Error (ppm)	Find Cpds Algorithm
Sphingofungin B	390.2858	1.066	389.2783	C20 H39 N O6	389.2777	1.45	Find by Formula

Compound Chromatograms

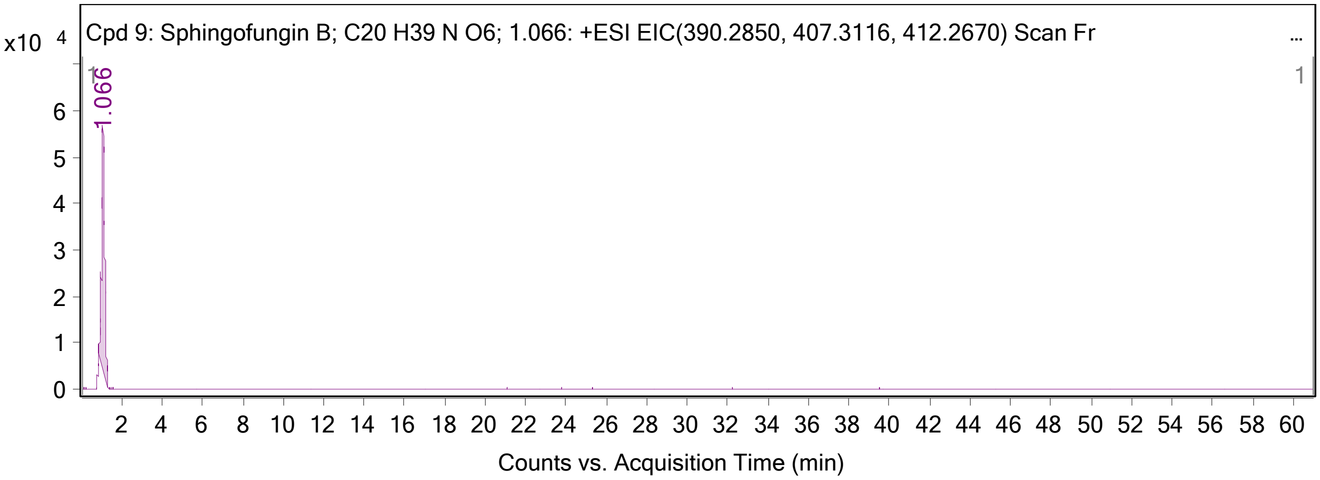

MS Zoomed Spectrum

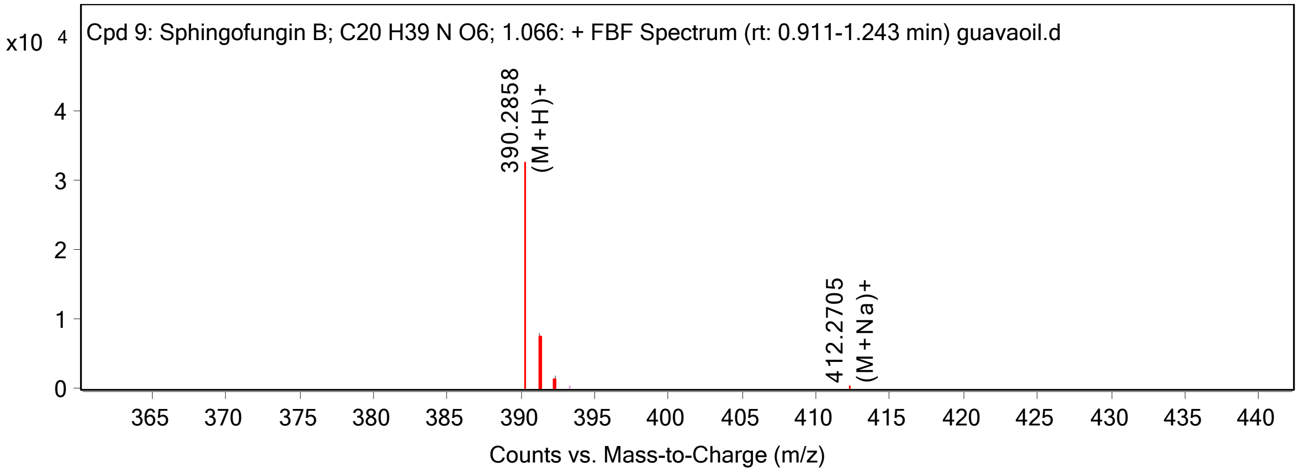

MS Spectrum Peak List

Obs. m/z	Charge	Abund	Ion/Isotope
390.2858	1	31257.39	(M+H)+
391.288	1	8067.59	(M+H)+
392.2908	1	1817.55	(M+H)+
412.2705	1	171.49	(M+Na)+

MS Zoomed Spectrum

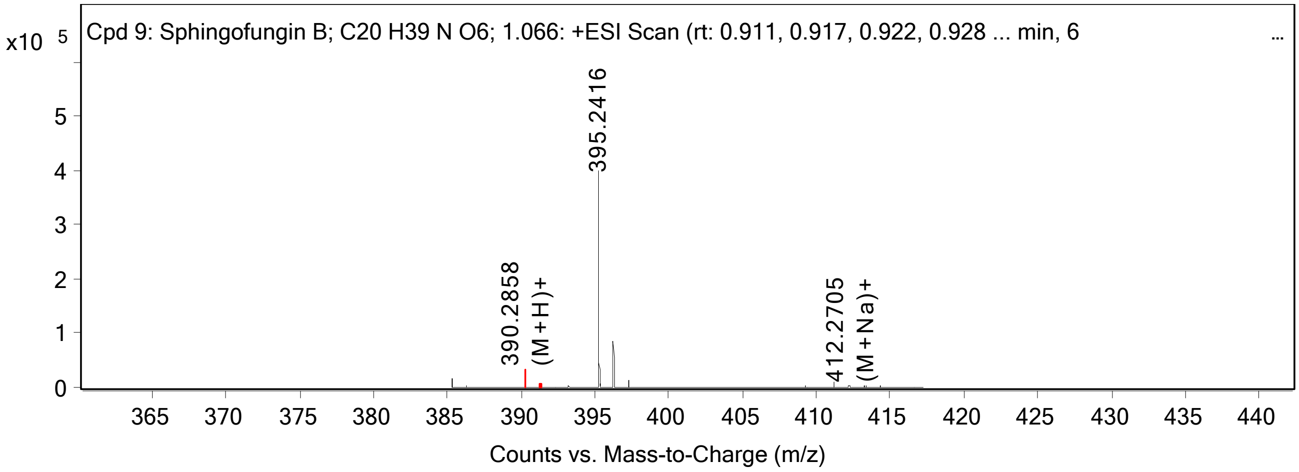

MS Spectrum Peak List

Obs. *m/z*	Charge	Abund	Ion/Isotope	Tgt Mass Error (ppm)
390.2858	1	31257.39	(M+H)+	-2.07
391.288	1	8067.59	(M+H)+	0.78
392.2908	1	1817.55	(M+H)+	0.13
395.2416		409137.32		
412.2705	1	171.49	(M+Na)+	-8.68

Name	Obs. *m/z*	Obs. RT	Obs. Mass	Tgt Formula	Tgt Mass	Tgt Mass Error (ppm)	Find Cpds Algorithm
13,14-dihydro-19(R)-hydroxyPGE1	395.2415	1.094	372.252	C20 H36 O6	372.2512	2.19	Find by Formula

Compound Chromatograms

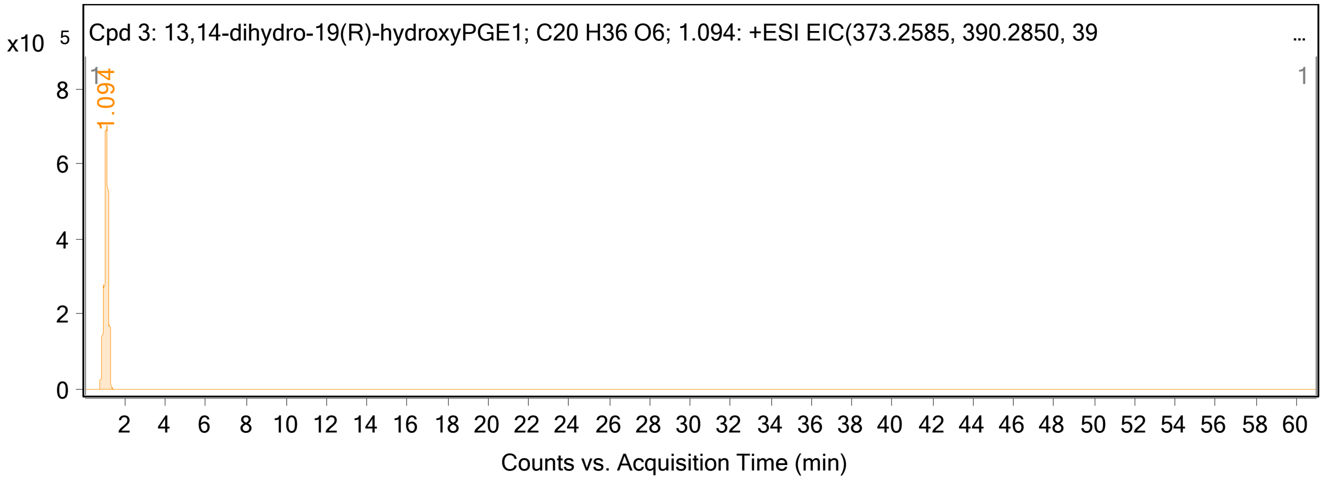

MS Zoomed Spectrum

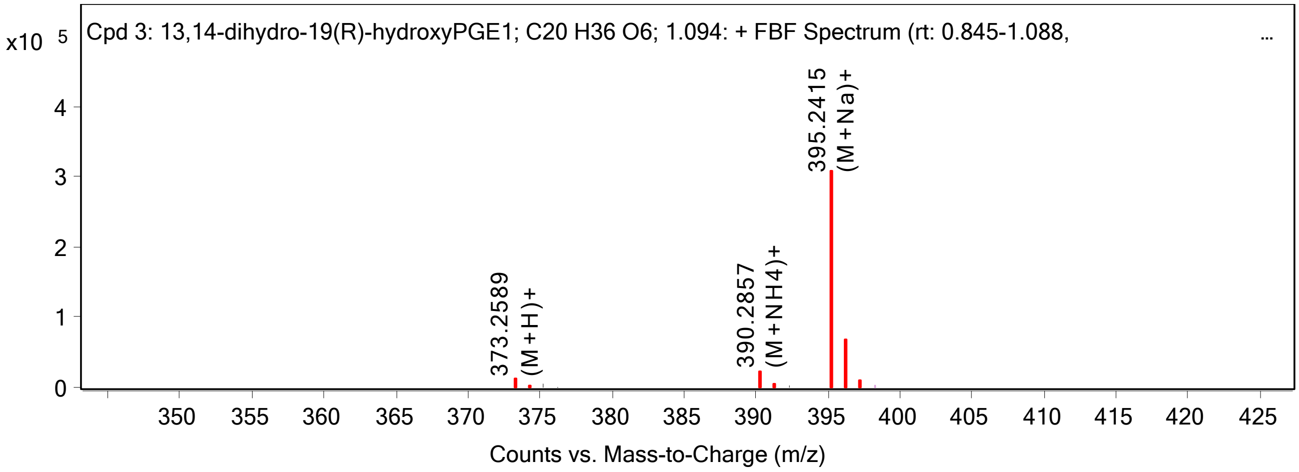

MS Spectrum Peak List

Obs. *m/z*	Charge	Abund	Ion/Isotope
373.2589	1	8921.8	(M+H)+
374.2622	1	1950.85	(M+H)+
375.2514	1	4412.21	(M+H)+
376.2545	1	1115.43	(M+H)+
390.2857	1	22566.39	(M+NH4)+
391.2877	1	6238.88	(M+NH4)+
392.2905	1	1453.82	(M+NH4)+
395.2415	1	308023.94	(M+Na)+
396.2448	1	66137.84	(M+Na)+
397.247	1	10238.69	(M+Na)+

MS Zoomed Spectrum

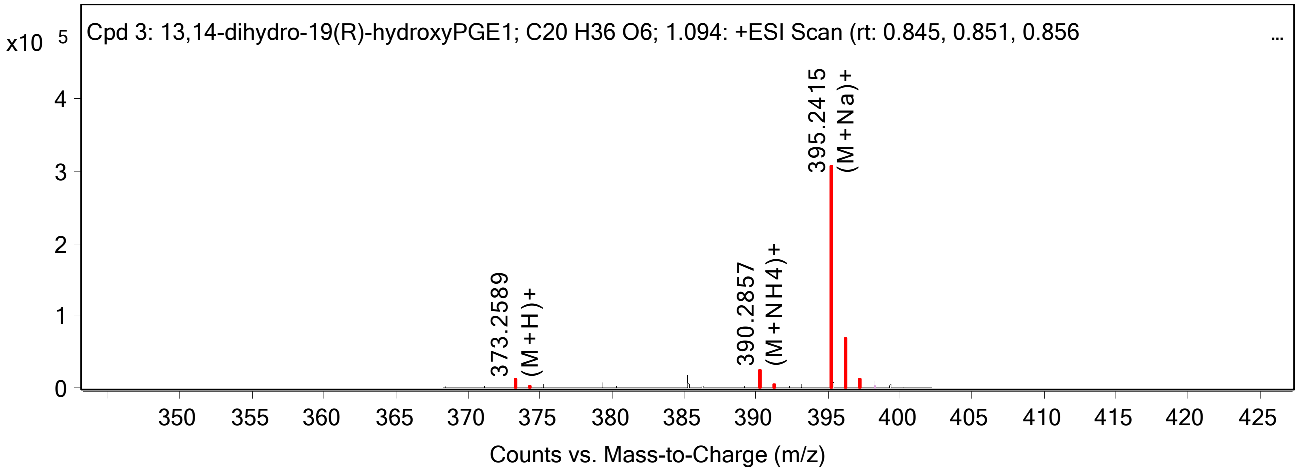

MS Spectrum Peak List

Obs. *m/z*	Charge	Abund	Ion/Isotope	Tgt Mass Error (ppm)
373.2589	1	8921.8	(M+H)+	-1.07
374.2622	1	1950.85	(M+H)+	-0.93
375.2514	1	4412.21	(M+H)+	34.7
376.2545	1	1115.43	(M+H)+	33.52
390.2857	1	22566.39	(M+NH4)+	-1.76
391.2877	1	6238.88	(M+NH4)+	1.66
392.2905	1	1453.82	(M+NH4)+	0.86
395.2415	1	308023.94	(M+Na)+	-2.8
396.2448	1	66137.84	(M+Na)+	-2.34
397.247	1	10238.69	(M+Na)+	-1.58

Name	Obs. *m/z*	Obs. RT	Obs. Mass	Tgt Formula	Tgt Mass	Tgt Mass Error (ppm)	Find Cpds Algorithm

Target Compound Screening Report

Eschscholtzxanthin	567.4231	1.177	566.4117	C40 H54 O2	566.4124	-1.18	Find by Formula

Compound Chromatograms

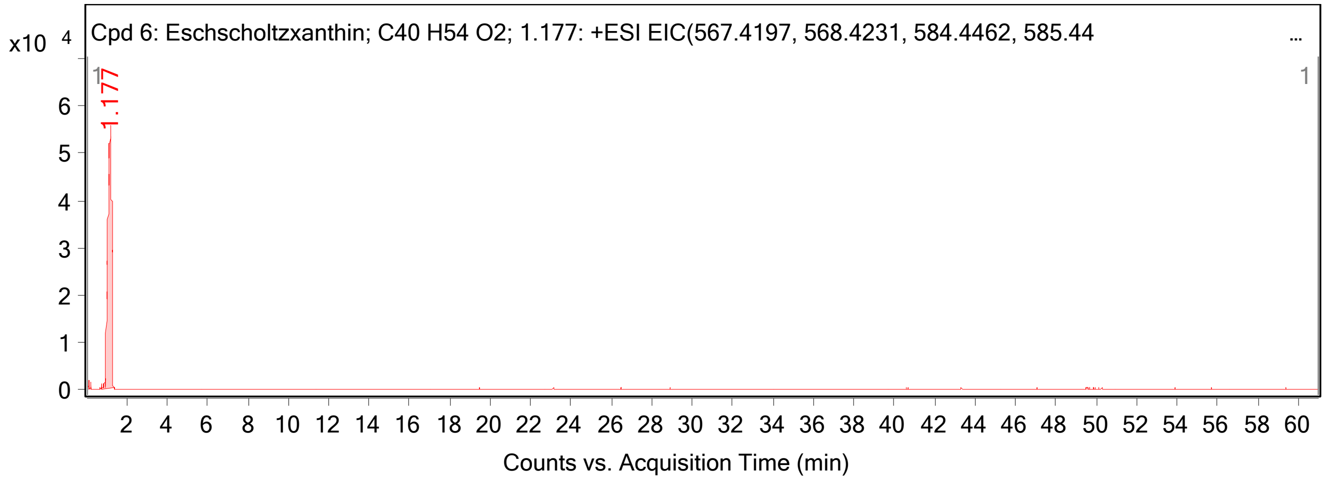

MS Zoomed Spectrum

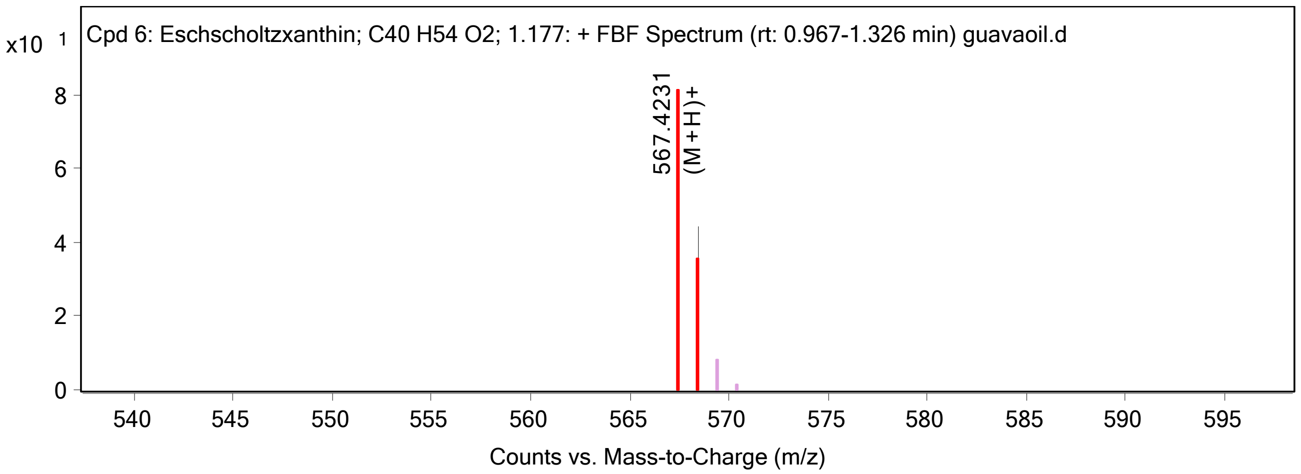

MS Spectrum Peak List

Obs. *m/z*	Charge	Abund	Ion/Isotope
567.4231	1	72.73	(M+H)+
568.4157	1	44.55	(M+H)+

MS Zoomed Spectrum

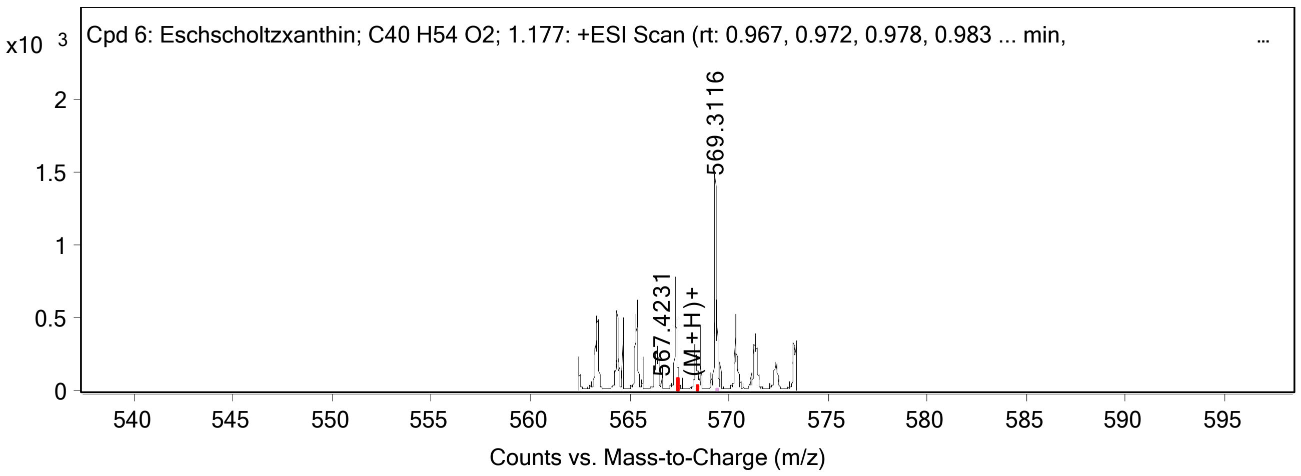

MS Spectrum Peak List

Obs. *m/z*	Charge	Abund	Ion/Isotope	Tgt Mass Error (ppm)
567.4231	1	72.73	(M+H)+	-6.08
568.4157	1	44.55	(M+H)+	12.99
569.3116		1542.45		

Name	Obs. *m/z*	Obs. RT	Obs. Mass	Tgt Formula	Tgt Mass	Tgt Mass Error (ppm)	Find Cpds Algorithm
Tetradecan-3-one	230.2483	21.926	212.2144	C14 H28 O	212.214	2.03	Find by Formula

Compound Chromatograms

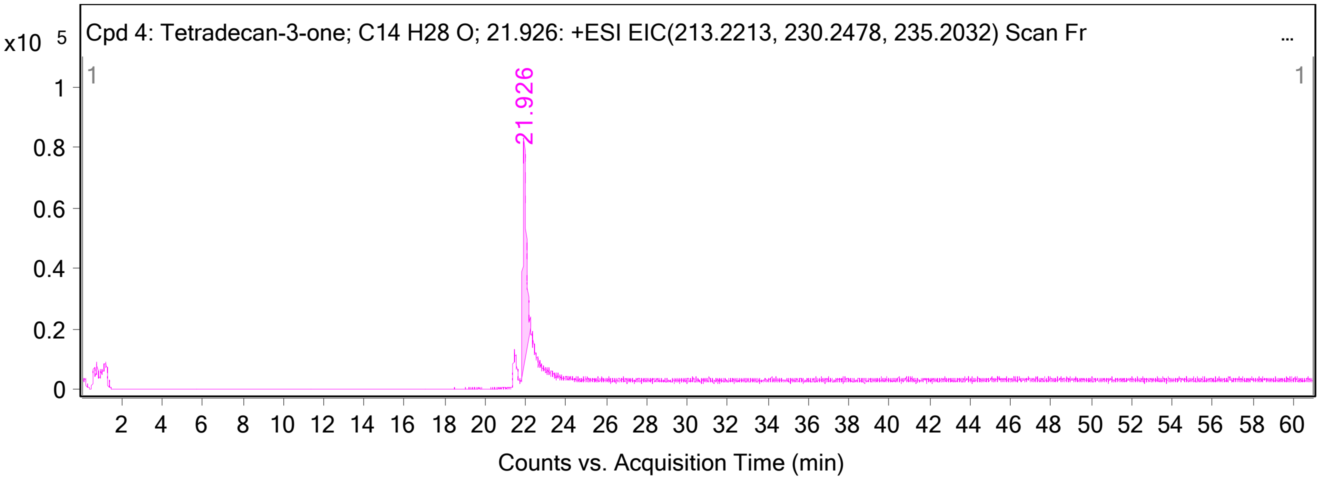

MS Zoomed Spectrum

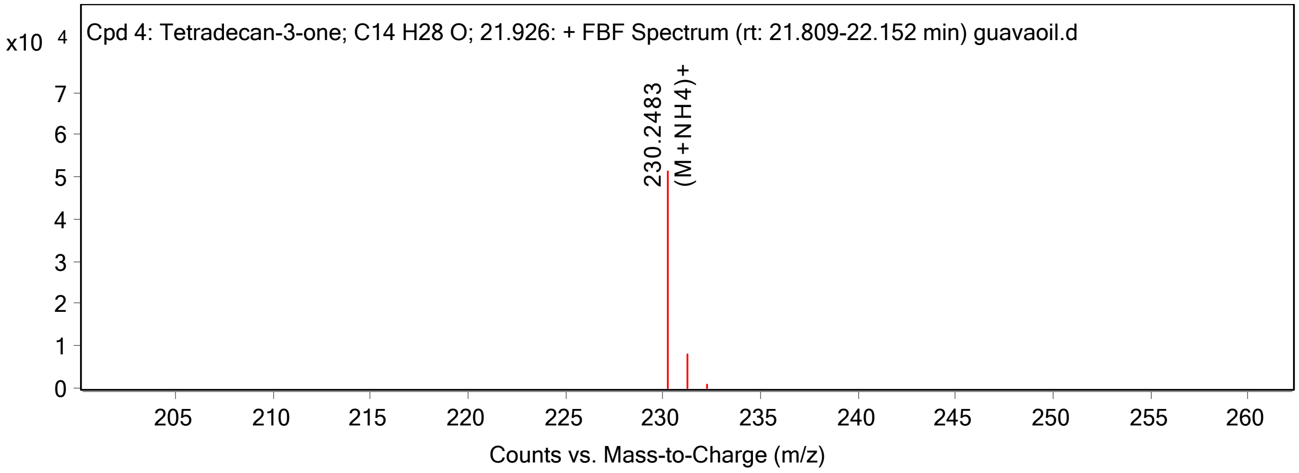

MS Spectrum Peak List

Obs. *m/z*	Charge	Abund	Ion/Isotope
230.2483	1	51444.05	(M+NH4)+
231.2515	1	7592.68	(M+NH4)+
232.2543	1	664.83	(M+NH4)+

MS Zoomed Spectrum

Target Compound Screening Report

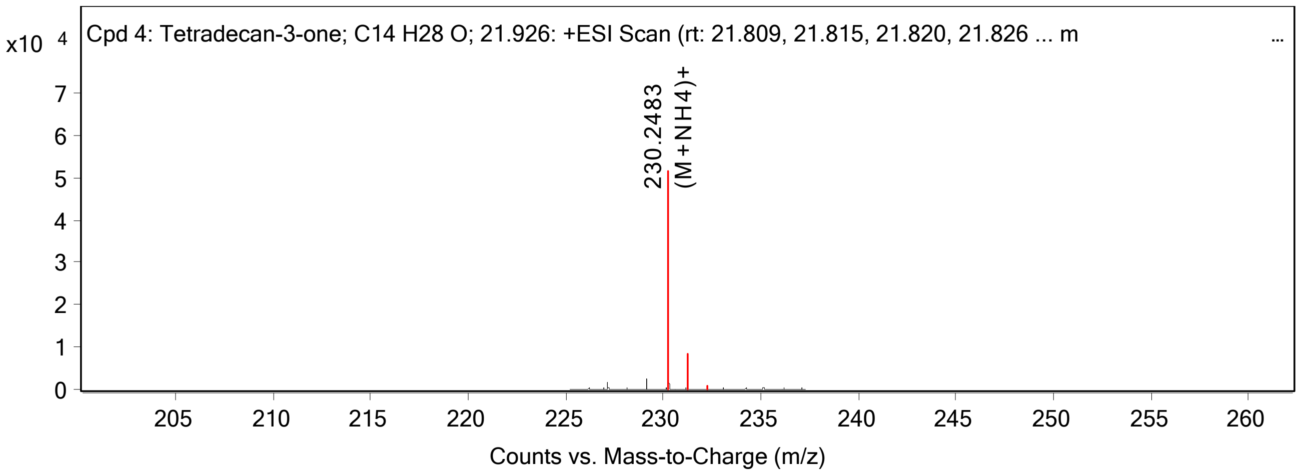

MS Spectrum Peak List

Obs. *m/z*	Charge	Abund	Ion/Isotope	Tgt Mass Error (ppm)
230.2483		51441.07		
230.2483	1	51444.05	(M+NH4)+	-1.91
231.2515	1	7592.68	(M+NH4)+	-1.68
232.2543	1	664.83	(M+NH4)+	-0.95

Name	Obs. *m/z*	Obs. RT	Obs. Mass	Tgt Formula	Tgt Mass	Tgt Mass Error (ppm)	Find Cpds Algorithm
Xestoaminol C	230.2483	21.926	229.241	C14 H31 N O	229.2406	1.88	Find by Formula

Compound Chromatograms

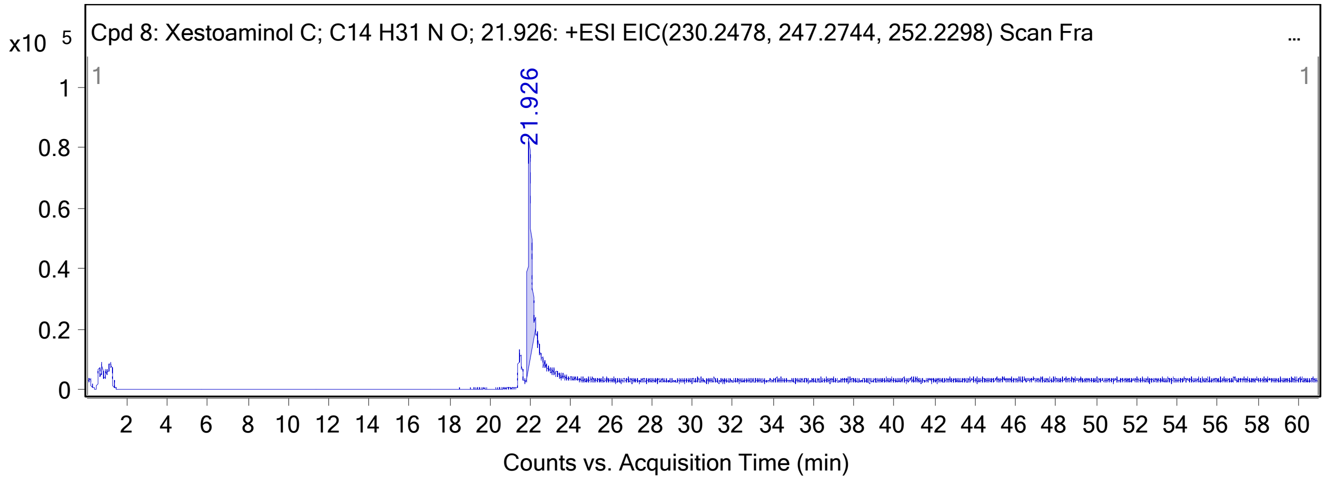

MS Zoomed Spectrum

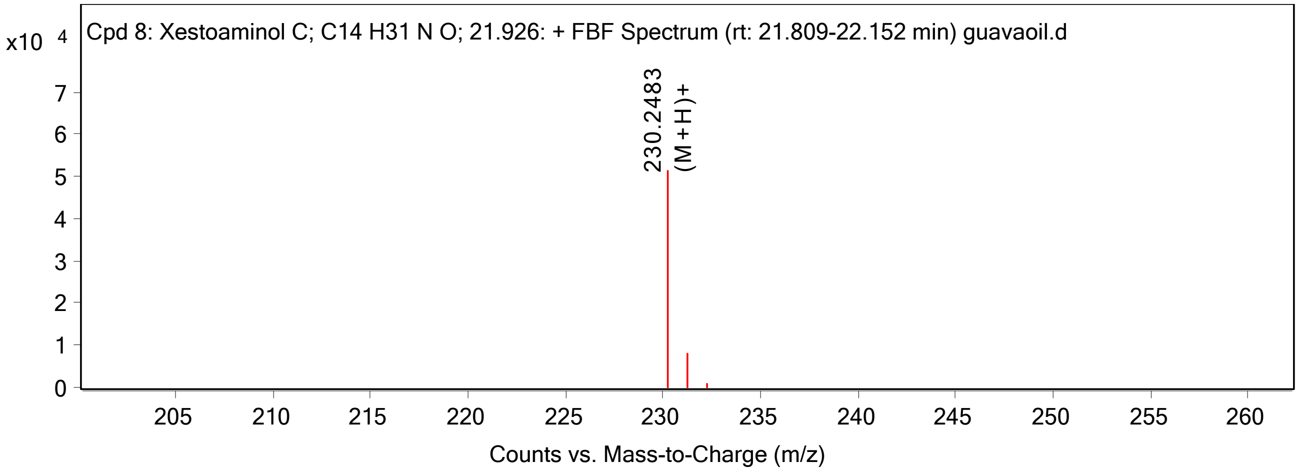

MS Spectrum Peak List

Obs. *m/z*	Charge	Abund	Ion/Isotope
230.2483	1	51444.05	(M+H)+
231.2515	1	7592.68	(M+H)+
232.2543	1	664.83	(M+H)+

MS Zoomed Spectrum

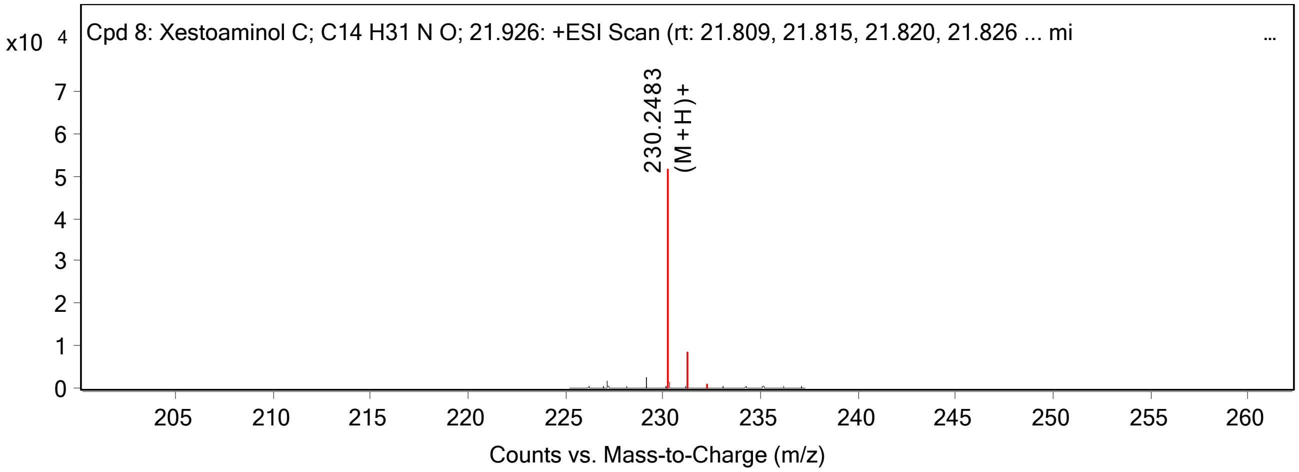

MS Spectrum Peak List

Obs. *m/z*	Charge	Abund	Ion/Isotope	Tgt Mass Error (ppm)
230.2483		51441.07		
230.2483	1	51444.05	(M+H)+	-1.91
231.2515	1	7592.68	(M+H)+	-1.68
232.2543	1	664.83	(M+H)+	-0.95

--- End Of Report ---
