# Supplementary material for: Phytosterol, Lipid and Phenolic Composition, and Biological Activities of Guava Seed Oil
Source: Molecules. 2020 May 27;25(11):2474. doi: 10.3390/molecules25112474 (PMC7321134; doi:10.3390/molecules25112474)

File :
Operator :
Acquired :
Instrument :
Sample Name:
Misc Info :
Vial Number: 6

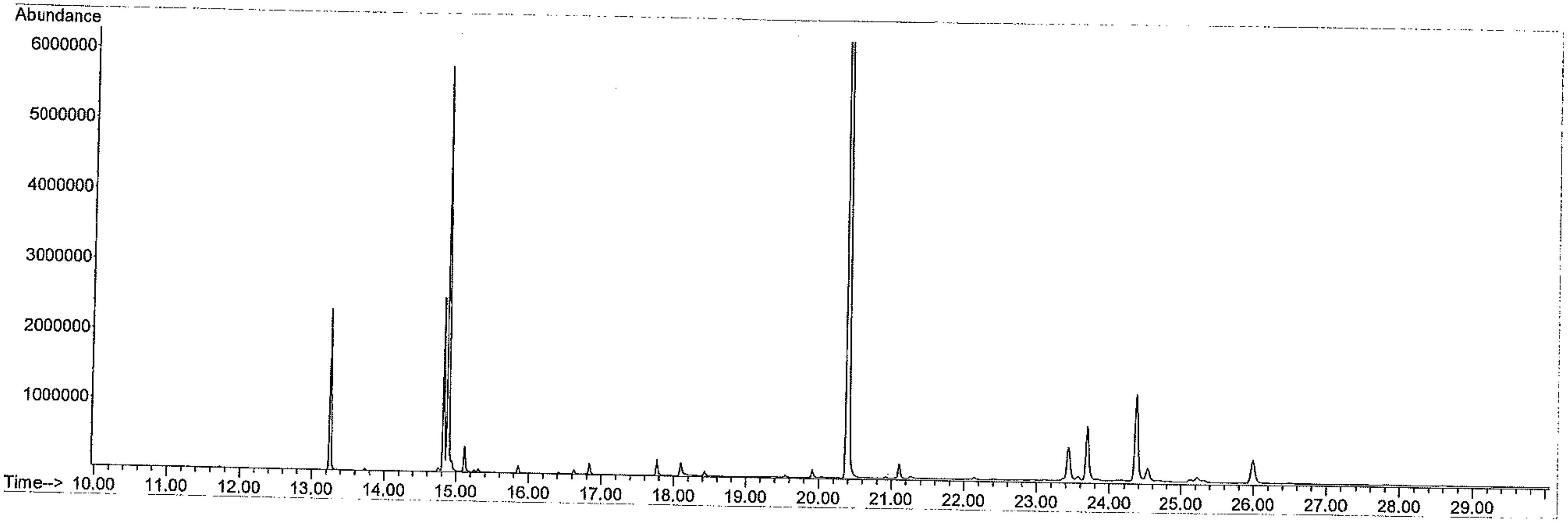

File :
Operator :
Acquired :
Instrument :
Sample Name:
Misc Info :
Vial Number: 6

m/z 13.25 min

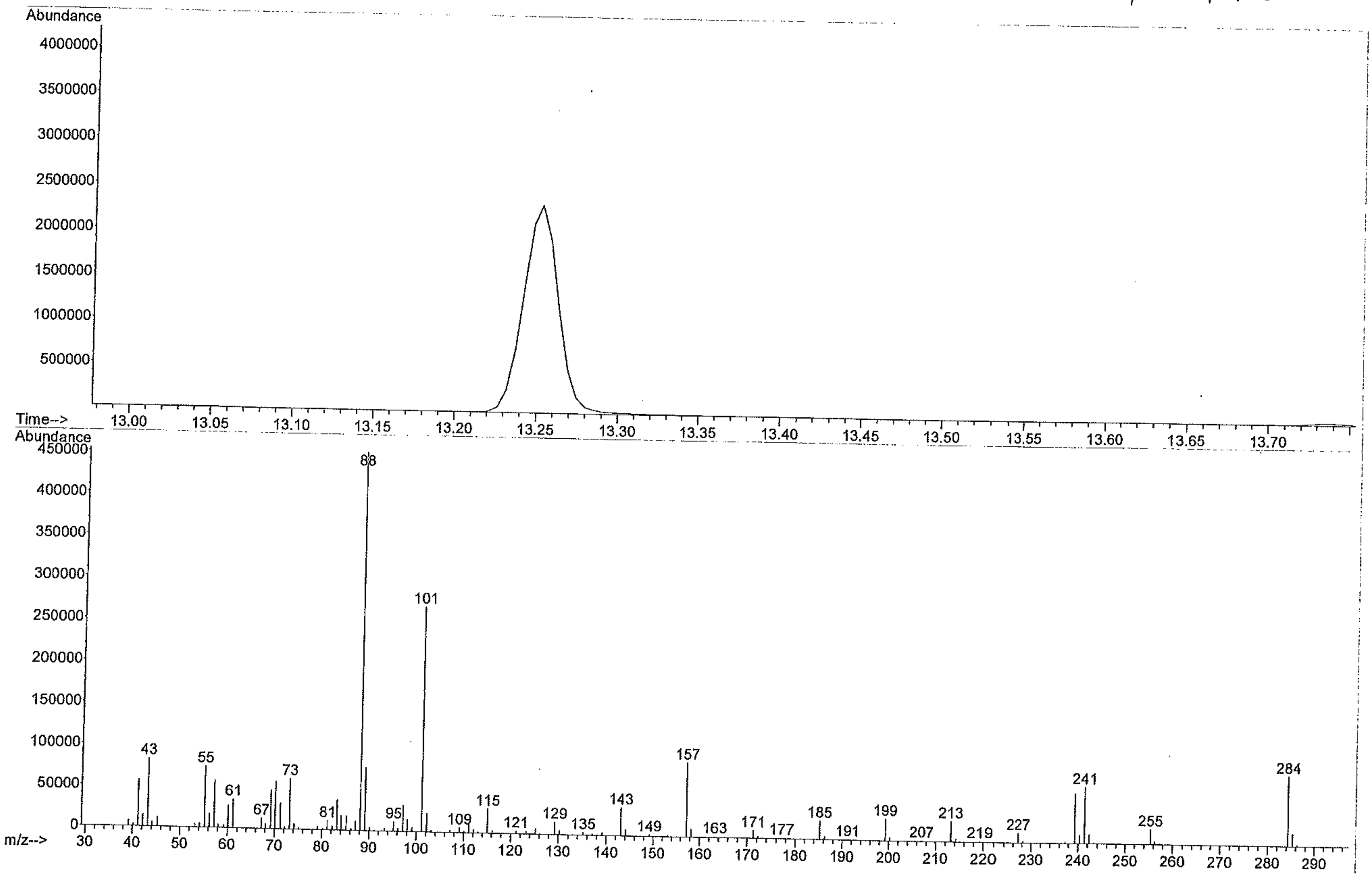

File :
Operator :
Acquired :
Instrument :
Sample Name:
Misc Info :
Vial Number: 6

M/Z 14.83 min

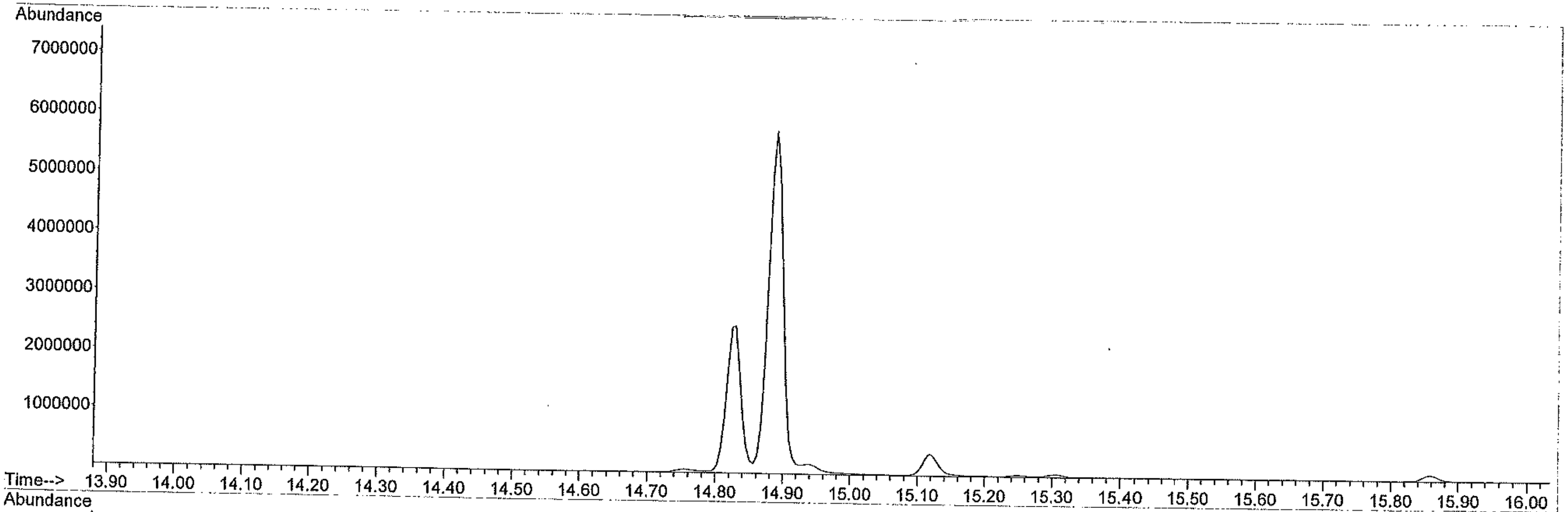

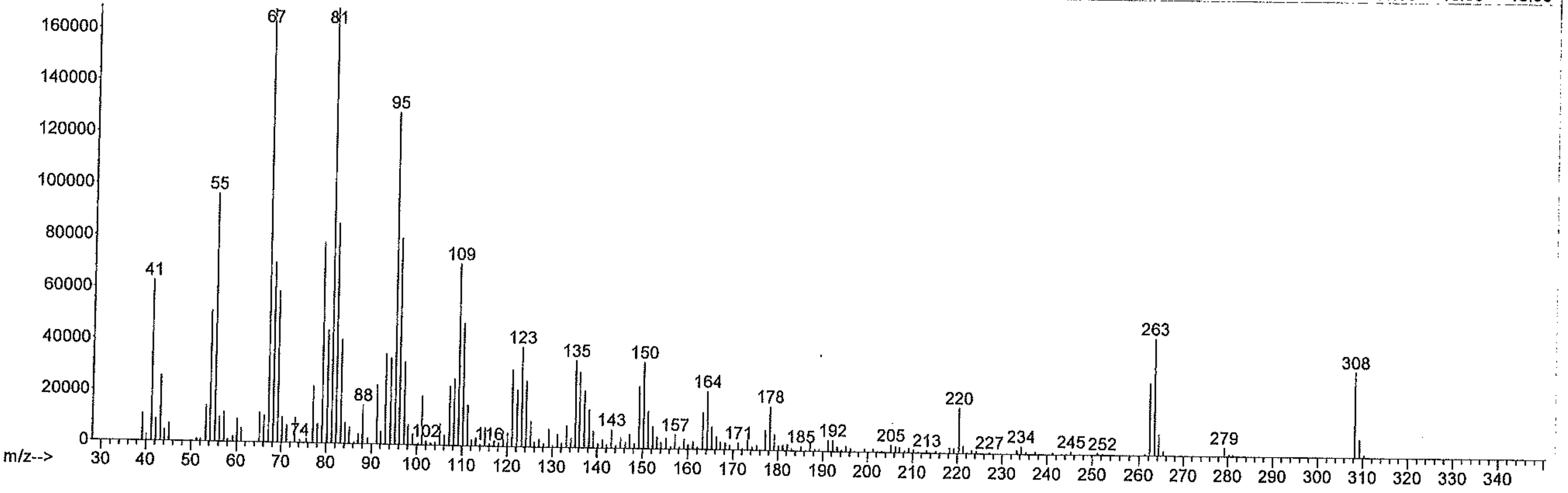

File :
Operator :
Acquired :
Instrument :
Sample Name:
Misc Info :
Vial Number: 6

m/z 14,89 min

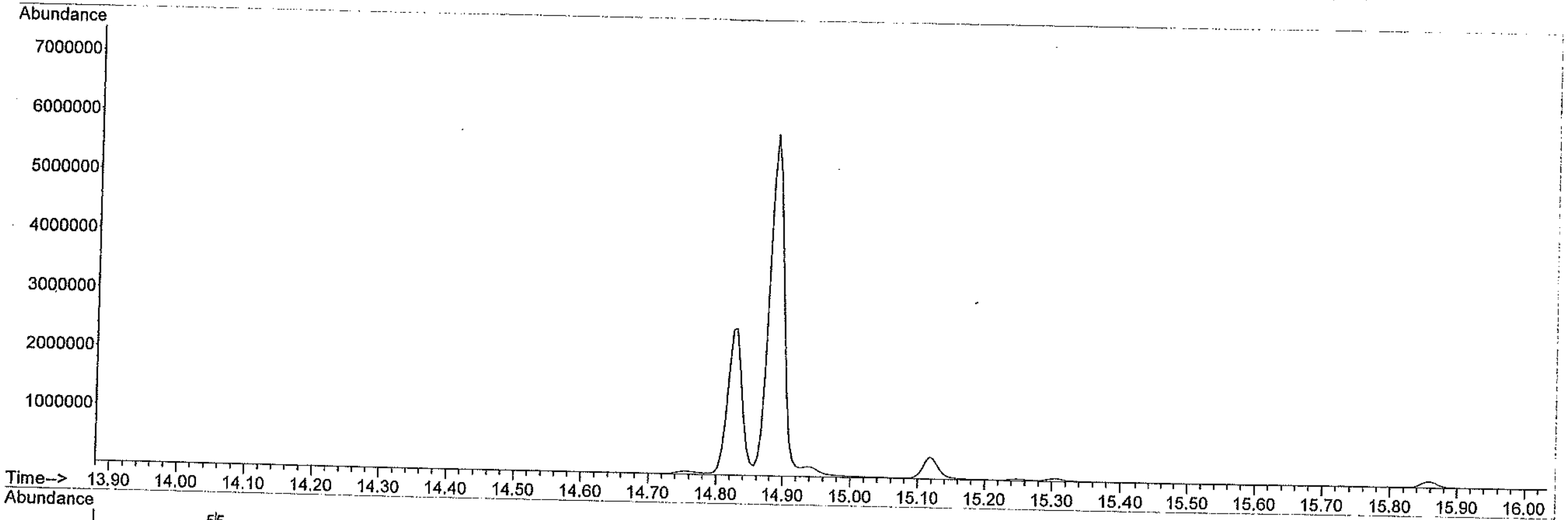

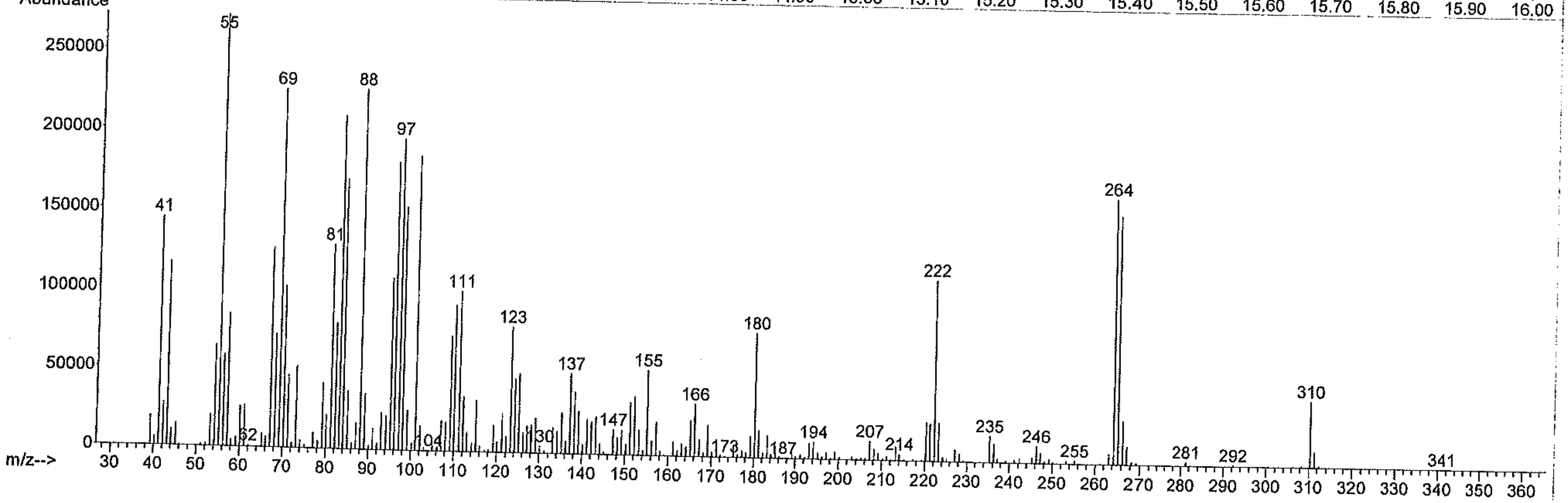

File :
Operator :
Acquired :
Instrument :
Sample Name:
Misc Info :
Vial Number: 6

M/z 15.12 min.

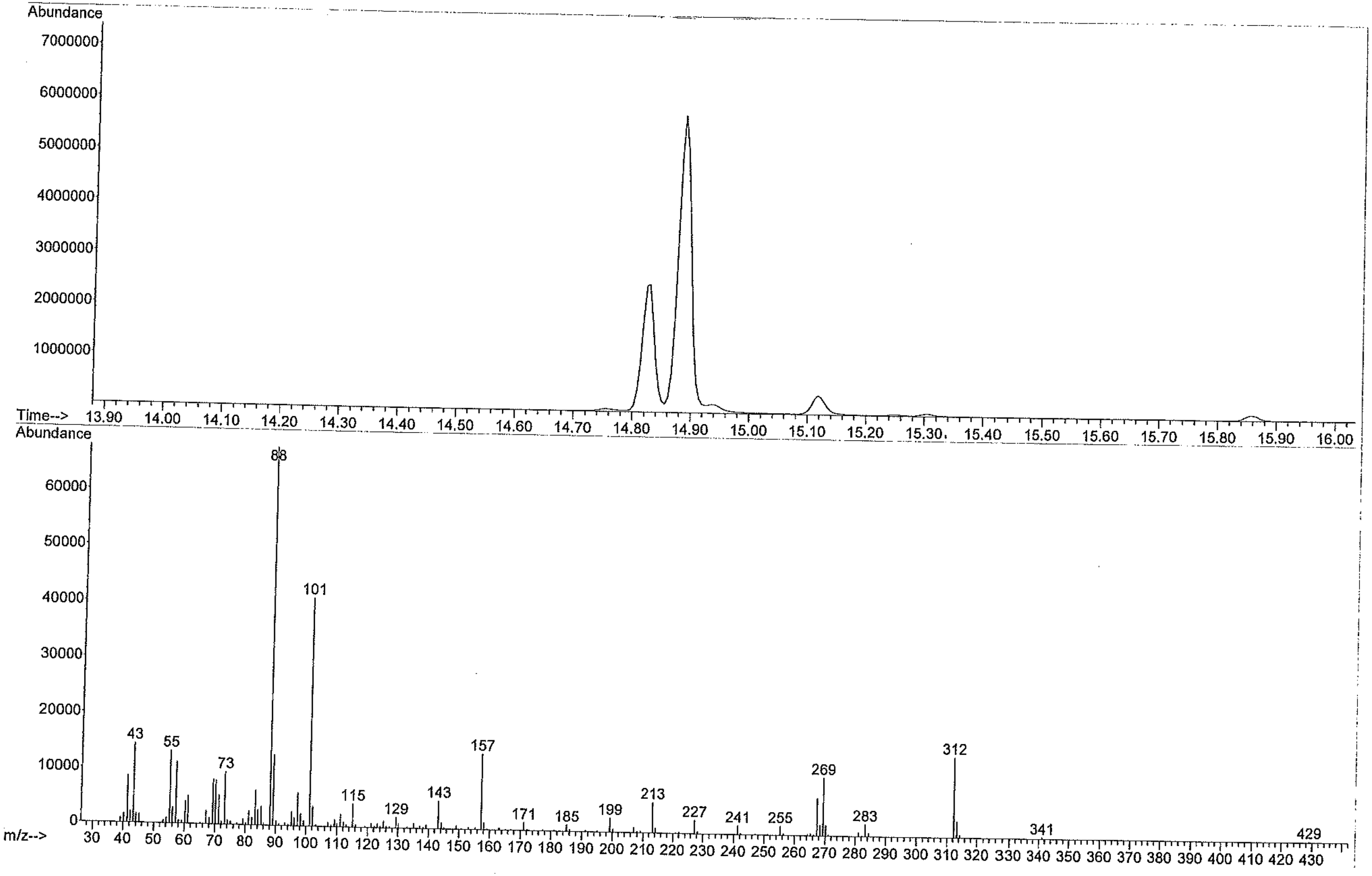

File :
Operator :
Acquired :
Instrument :
Sample Name:
Misc Info :
Vial Number: 6

M/z 17.77 min

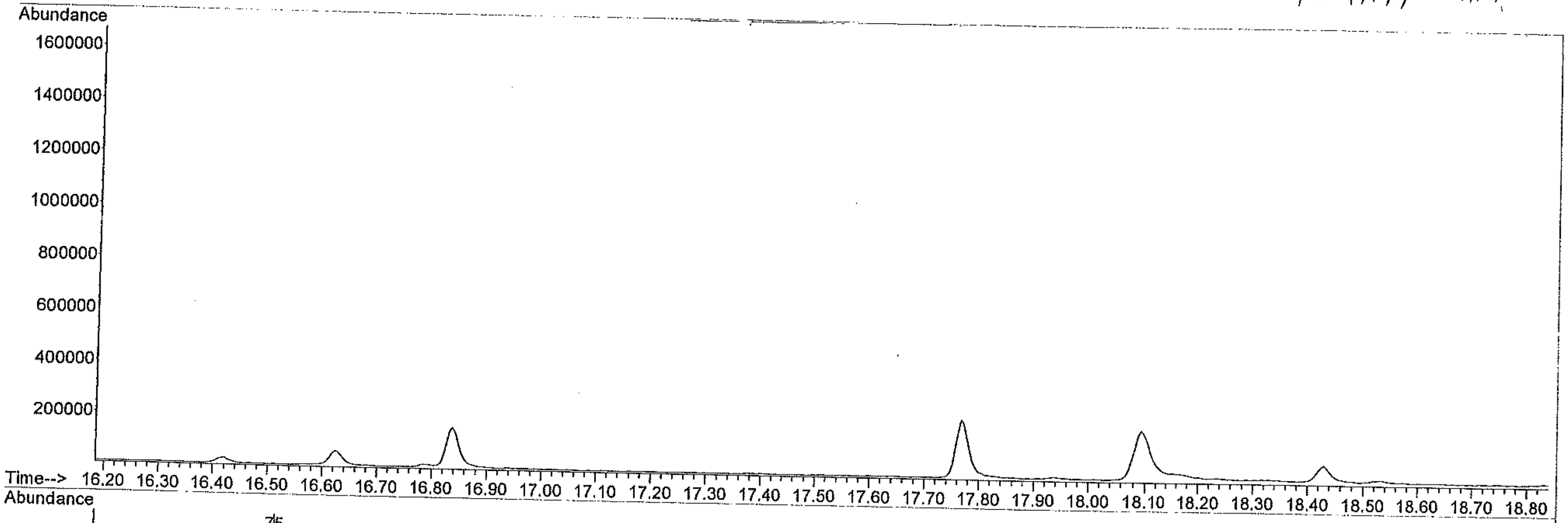

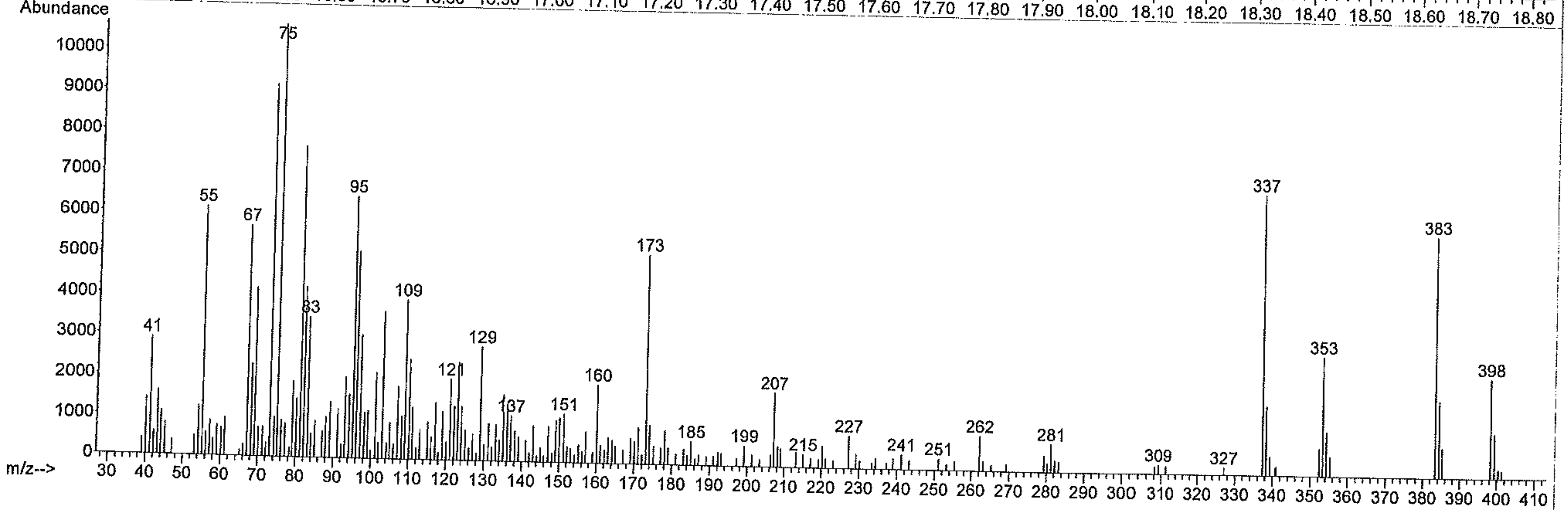

File :(
Operator :
Acquired :
Instrument :
Sample Name:
Misc Info :
Vial Number: 6

M/A 18.10 min.

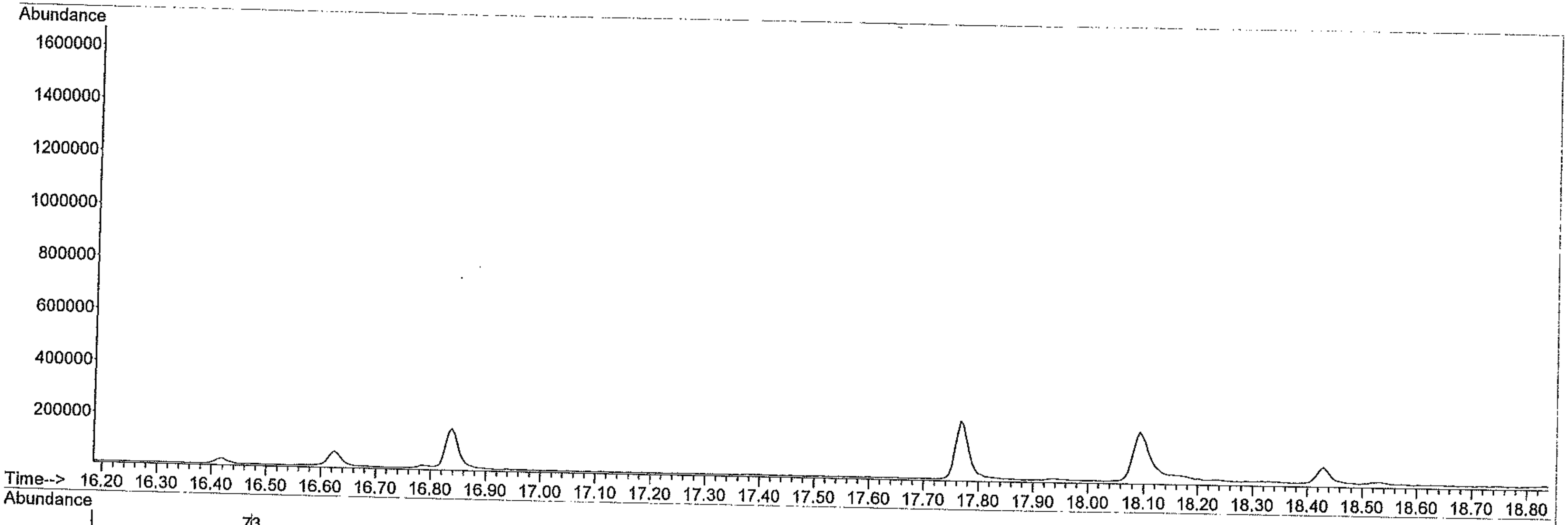

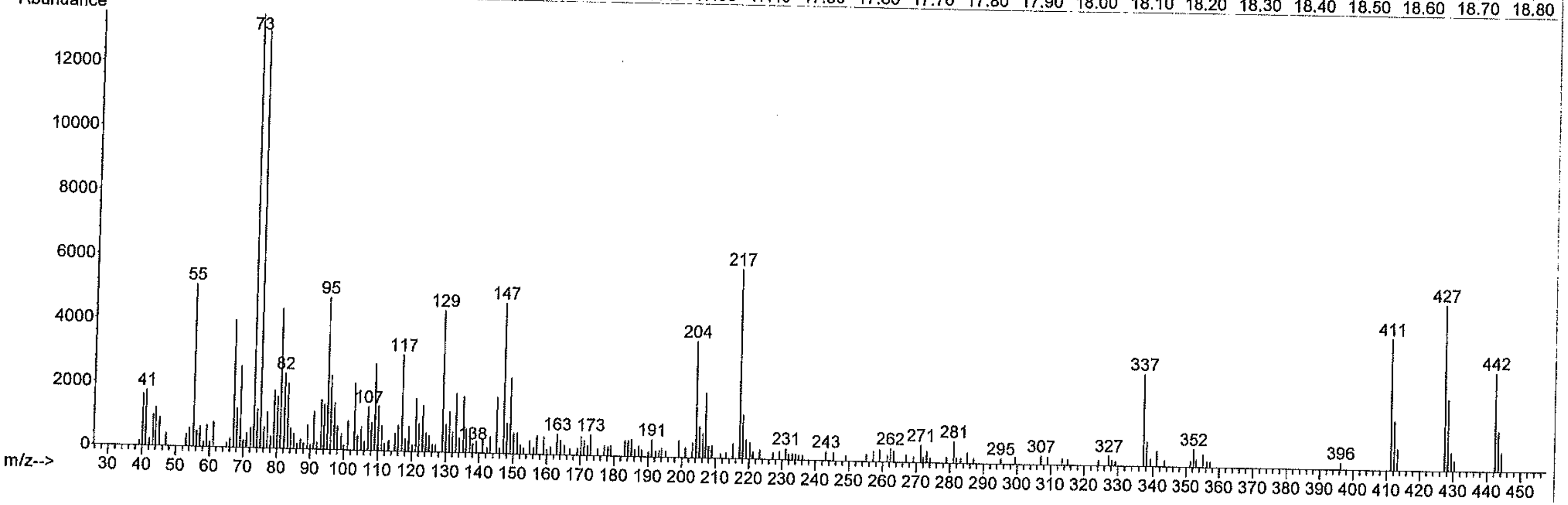

File :
Operator :
Acquired :
Instrument :
Sample Name:
Misc Info :
Vial Number: 6

(Internal standard)
m/z 20.41 min

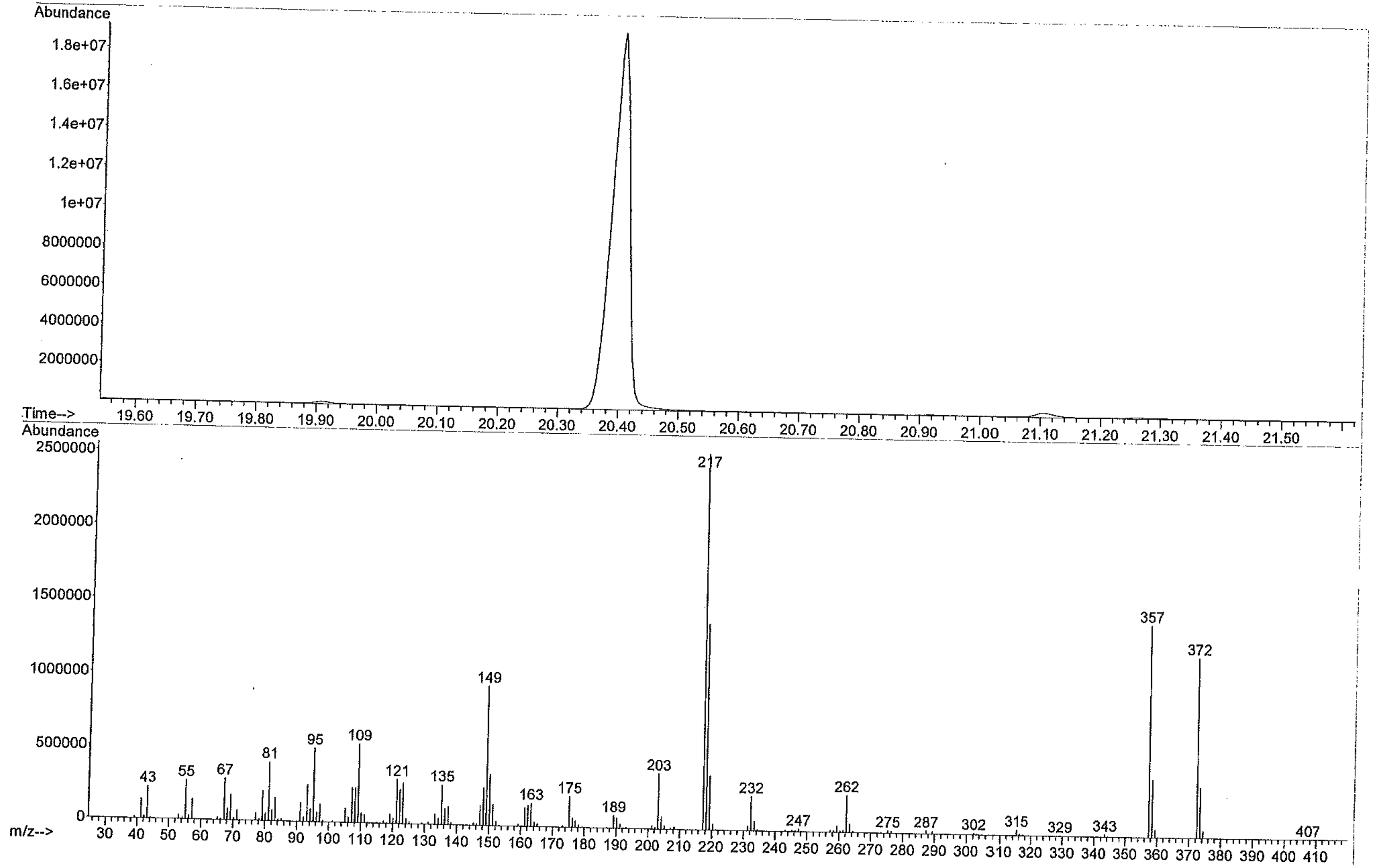

File
Operator :
Acquired :
Instrument :
Sample Name:
Misc Info :
Vial Number: 6

M/E 23.70 min.

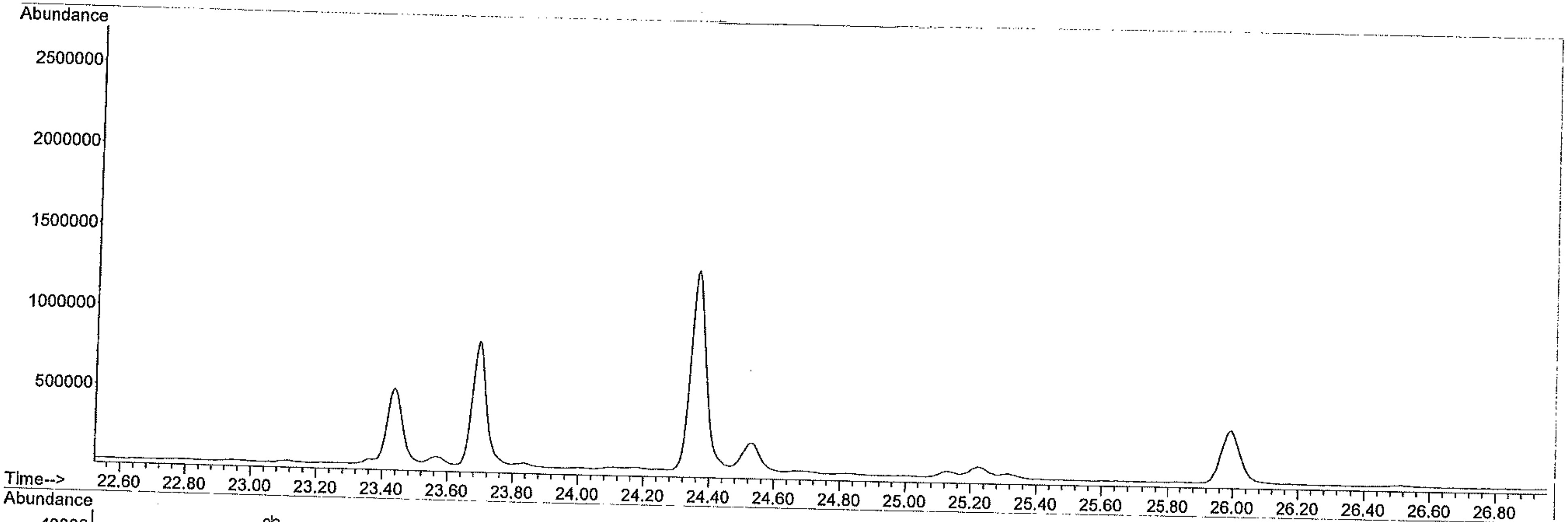

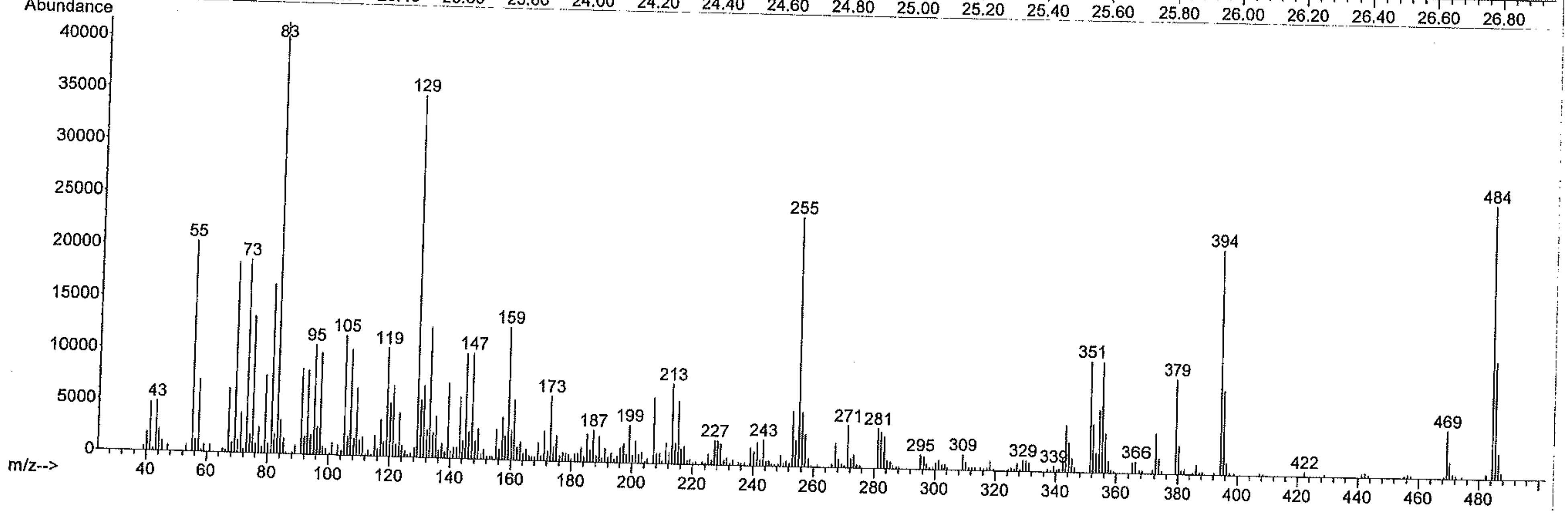

File :
Operator :
Acquired :
Instrument :
Sample Name:
Misc Info :
Vial Number: 6

M/z = 24.37 min.

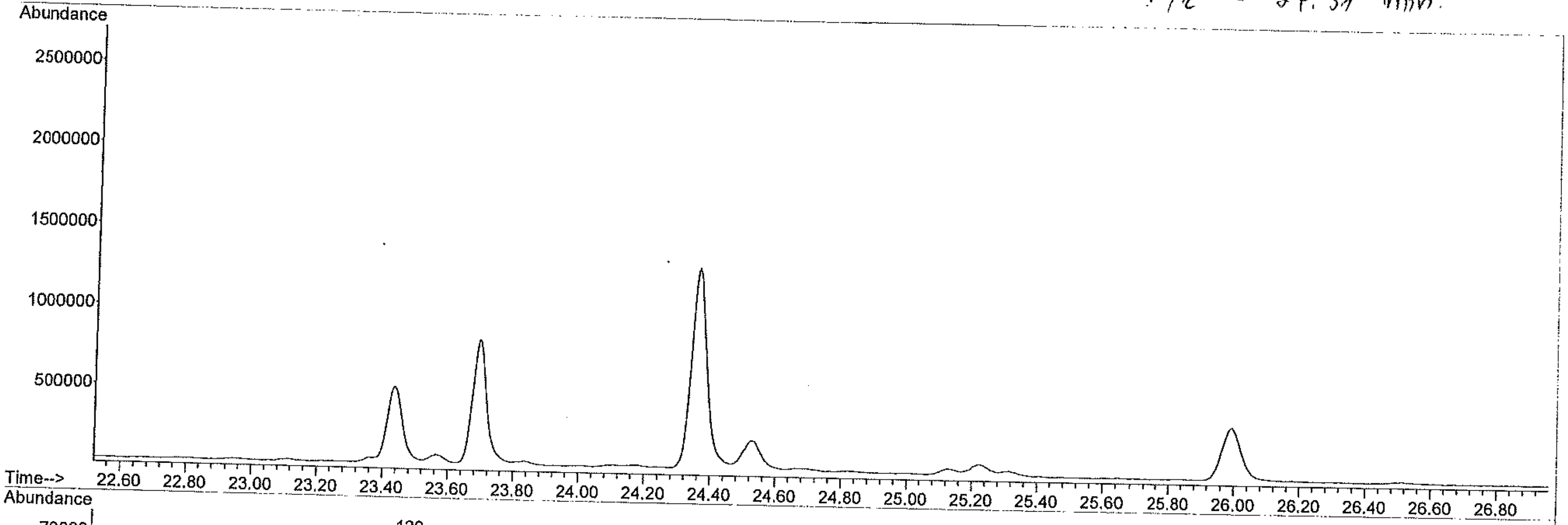

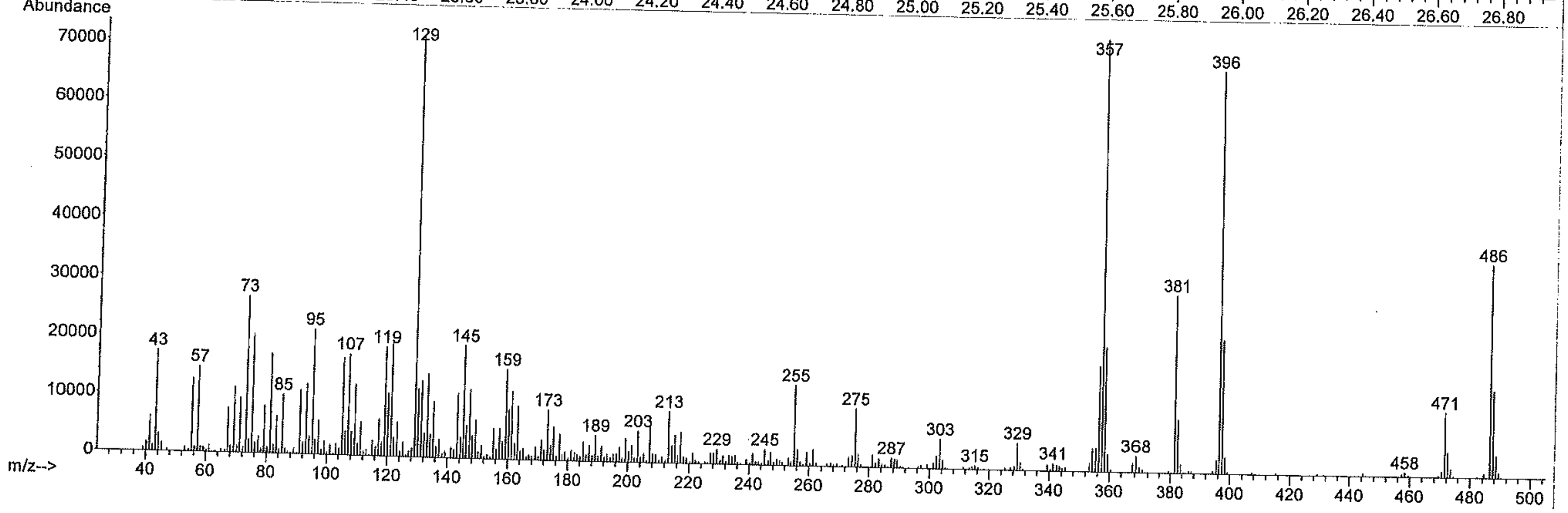

File :
Operator :
Acquired :
Instrument :
Sample Name:
Misc Info :
Vial Number: 6

Campesterol - TMS.
m/z 23,44 min,

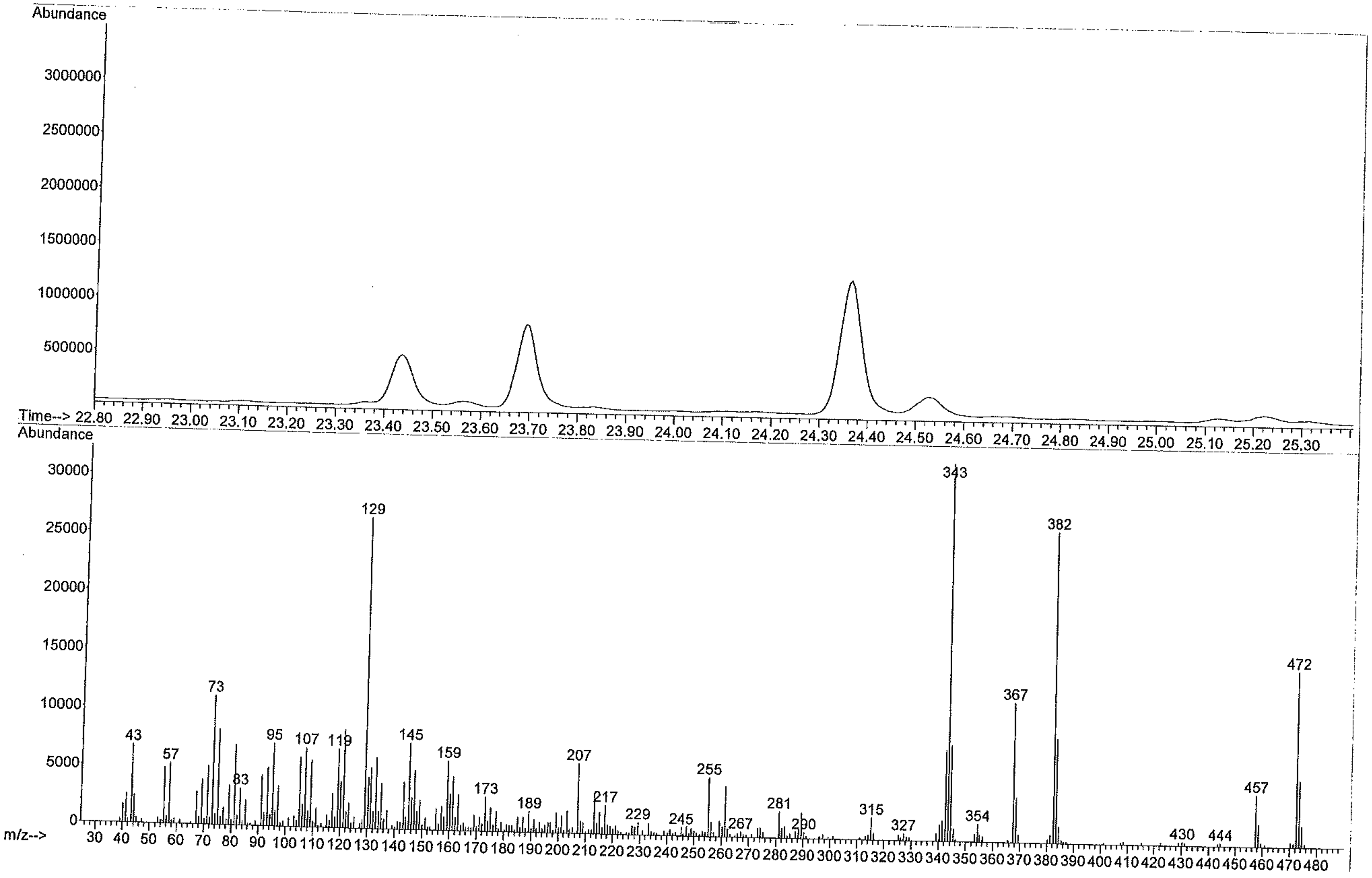

Supplement: Supplementary file 1 [file molecules-25-02474-s001.zip › molecules-804750-final-SM/Figure S2 GC MS for phytosterols of GSO.pdf]
