# Supplementary material for: Phytosterol, Lipid and Phenolic Composition, and Biological Activities of Guava Seed Oil
Source: Molecules. 2020 May 27;25(11):2474. doi: 10.3390/molecules25112474 (PMC7321134; doi:10.3390/molecules25112474)
Supplement: Supplementary file 1 [file molecules-25-02474-s001.zip › molecules-804750-final-SM/Figure S3a LC-ESI MS for phenolics of GSO.pdf]

Print of all graphic windows

Current Chromatogram(s)

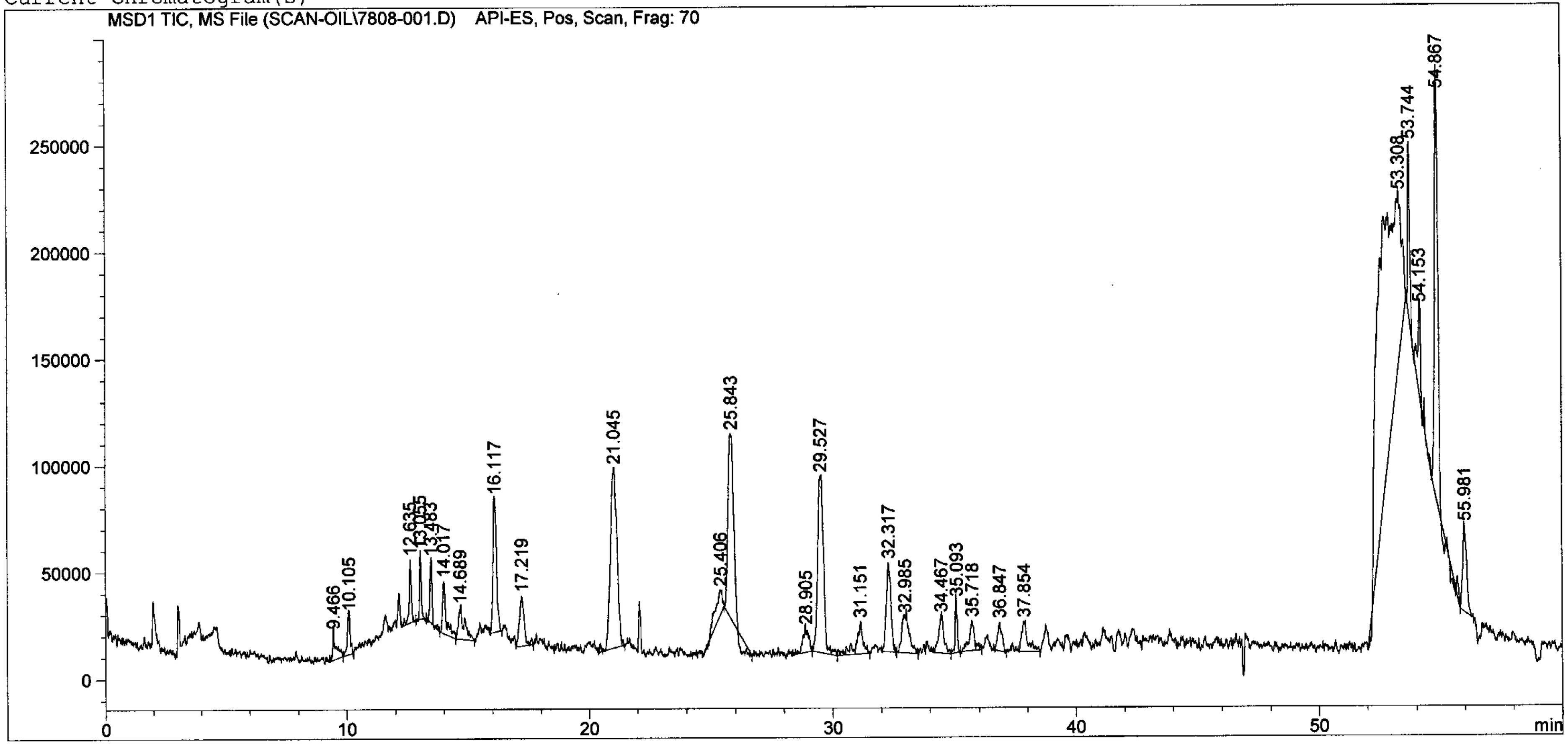

น้ำมันเมล็ดปริ้ง CM56/07802-001

(Hexane extract)

Instrument 1 4/25/2014 2:15:30 PM Somsak

Data File C:\HPCHEM\1\DATA\SCAN-OIL\7808-001.D

Sample Name: CM57/078082
56

Integration Results

Signal 1: MSD1 TIC, MS File

Peak #	Time [min]	Type	Area	Height	Width [min]	Start [min]	End [min]
1	9.466	BB	1.53318e5	1.21966e4	0.1725	9.288	9.860
2	10.105	BB	1.74050e5	1.79398e4	0.1605	9.860	10.286
3	12.635	BB	1.84713e5	2.90229e4	0.1104	12.268	12.859
4	13.055	BB	1.51539e5	3.04724e4	0.0869	12.859	13.286
5	13.483	BB	1.80435e5	2.89744e4	0.1016	13.286	13.842
6	14.017	BB	2.54445e5	2.40059e4	0.1598	13.842	14.503
7	14.689	BB	2.54204e5	1.43411e4	0.2430	14.503	15.265
8	16.117	BB	6.49376e5	6.21532e4	0.1653	15.889	16.395
9	17.219	BB	3.18340e5	2.29515e4	0.2197	16.978	17.692
10	21.045	BB	1.59604e6	8.38974e4	0.3015	20.375	21.479
11	25.843	BB	1.28623e6	8.63099e4	0.2463	25.553	26.680
12	28.905	BB	1.99554e5	1.08278e4	0.3164	28.359	29.161
13	29.527	BB	1.37631e6	8.23881e4	0.2673	29.161	30.184
14	31.151	BB	3.05829e5	1.45256e4	0.2818	30.184	31.523
15	32.317	BB	5.42999e5	4.13174e4	0.2113	32.016	32.643
16	32.985	BB	3.79396e5	1.63159e4	0.3365	32.643	33.514
17	34.467	BB	2.60752e5	1.68893e4	0.2236	34.139	34.859
18	35.093	BB	1.80178e5	2.39055e4	0.1233	34.859	35.251
19	35.718	BB	2.18514e5	1.33906e4	0.2338	35.251	36.125
20	36.847	BB	1.60442e5	1.28862e4	0.2103	36.580	37.115
21	37.854	BB	2.43069e5	1.35835e4	0.2666	37.601	38.502
22	53.308	BB	7.25794e6	7.97063e4	1.1202	52.023	53.621
23	53.744	BB	4.50582e5	8.19036e4	0.0931	53.621	53.944
24	54.153	BB	3.65551e5	4.01932e4	0.1423	53.944	54.638
25	54.867	BB	1.81503e6	2.03478e5	0.1475	54.638	55.791
26	55.981	BBA	4.66310e5	4.00731e4	0.1864	55.791	56.344

Print of all graphic windows

Current Chromatogram(s)

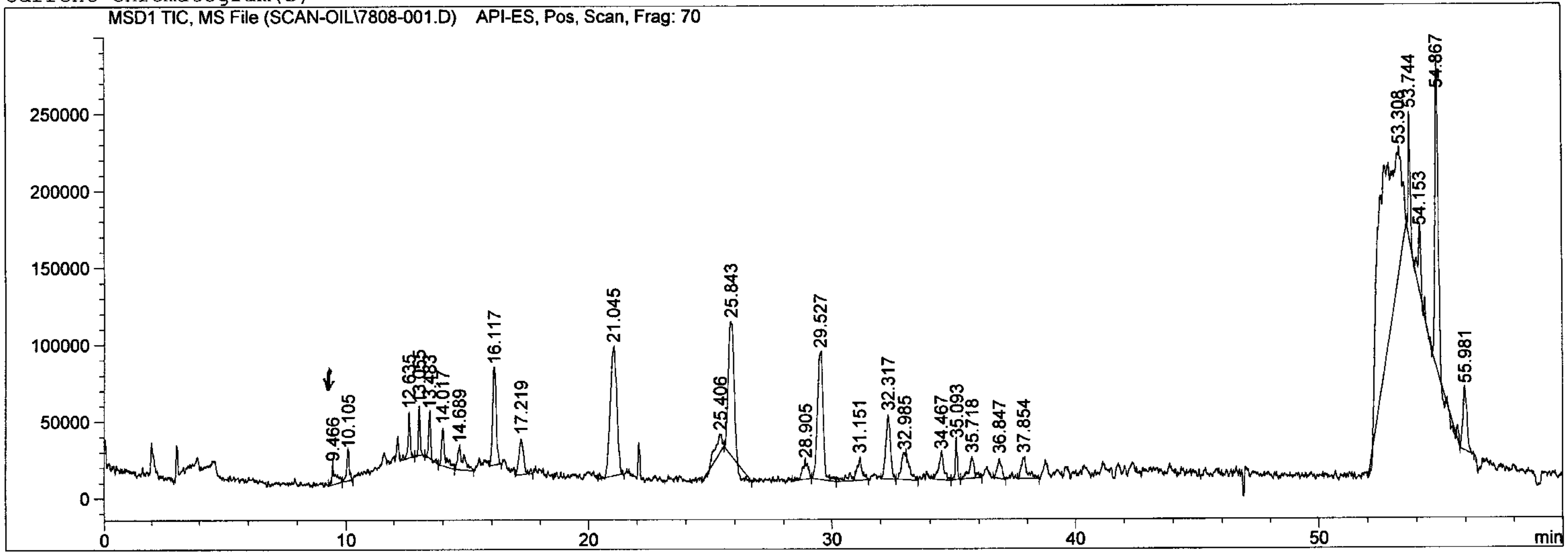

MS Spectrum

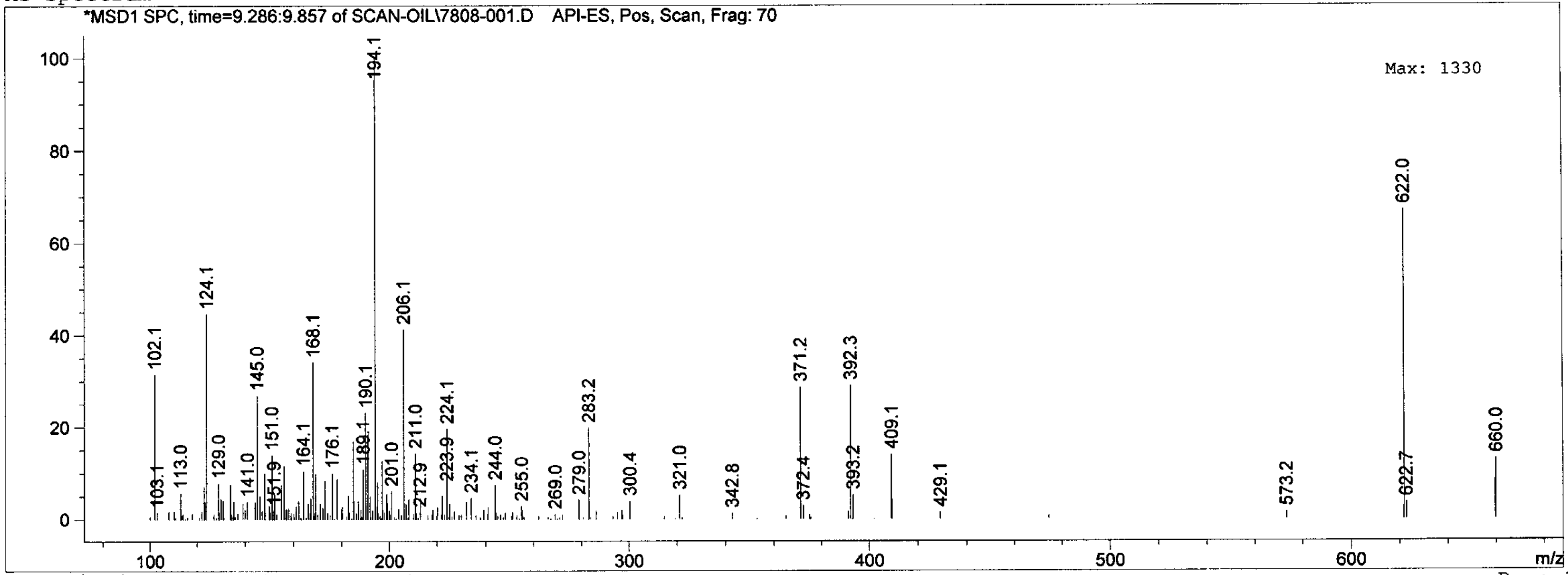

Print of all graphic windows

Current Chromatogram(s)

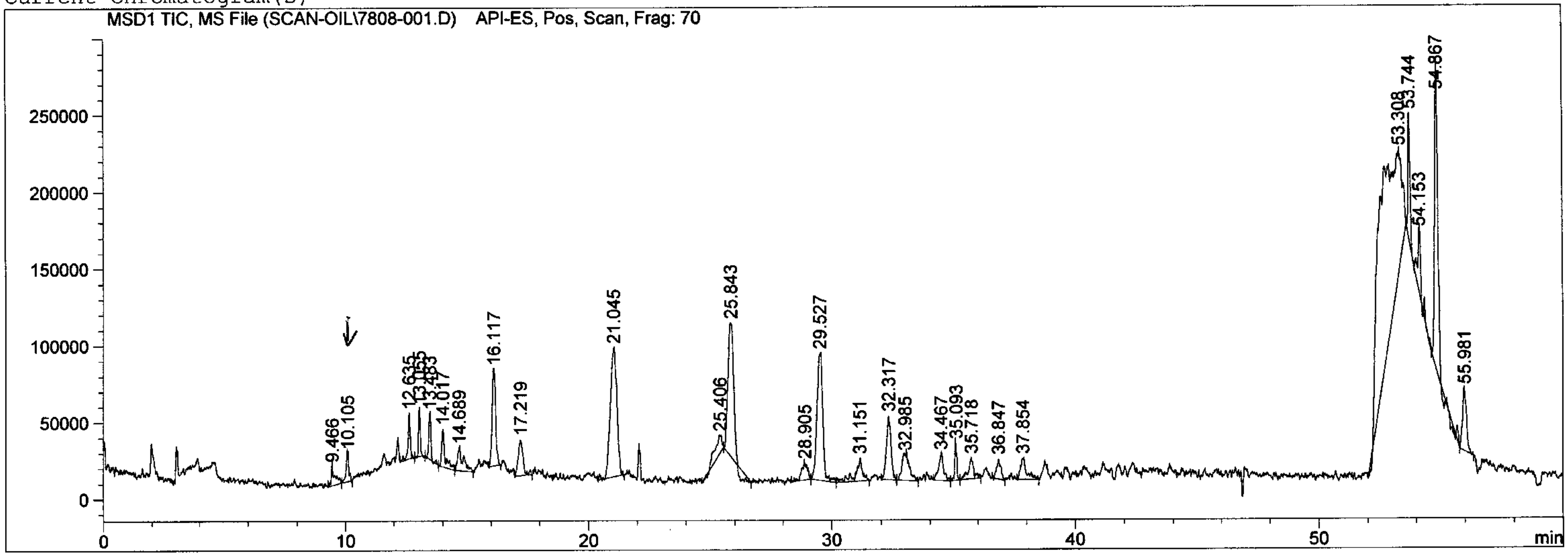

MS Spectrum

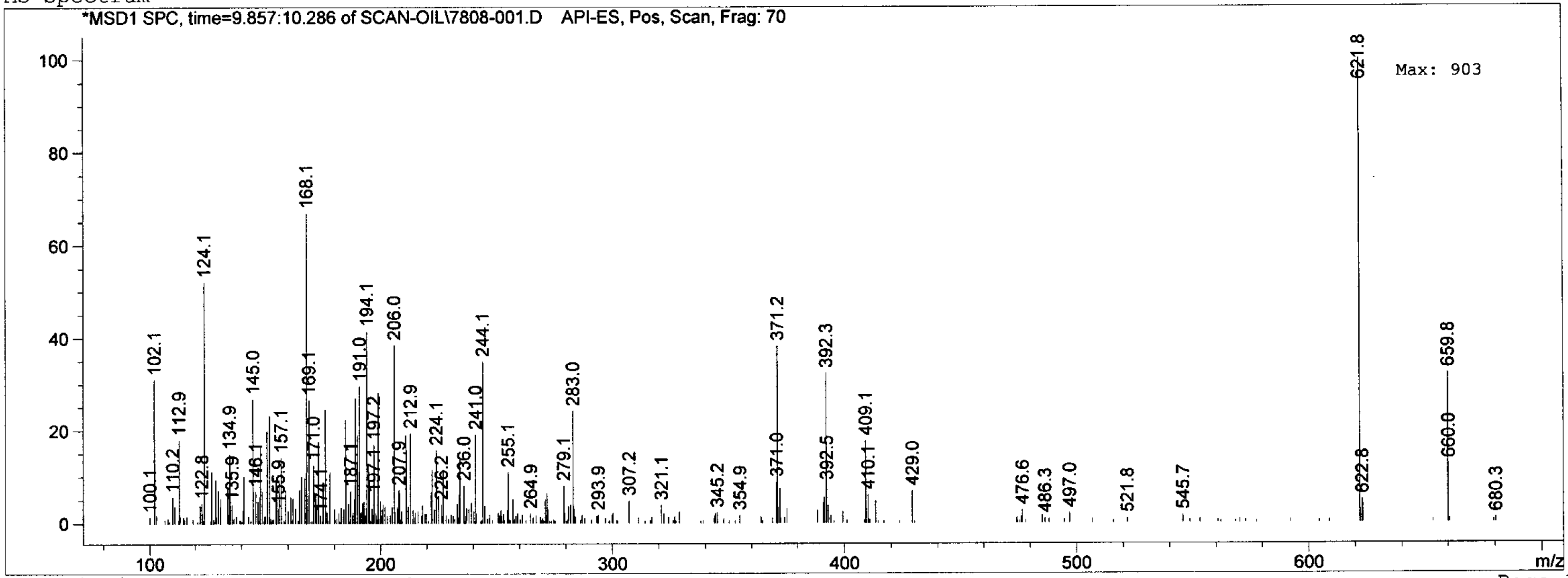

Instrument 1 4/25/2014 2:17:45 PM Somsak

Current Chromatogram(s)

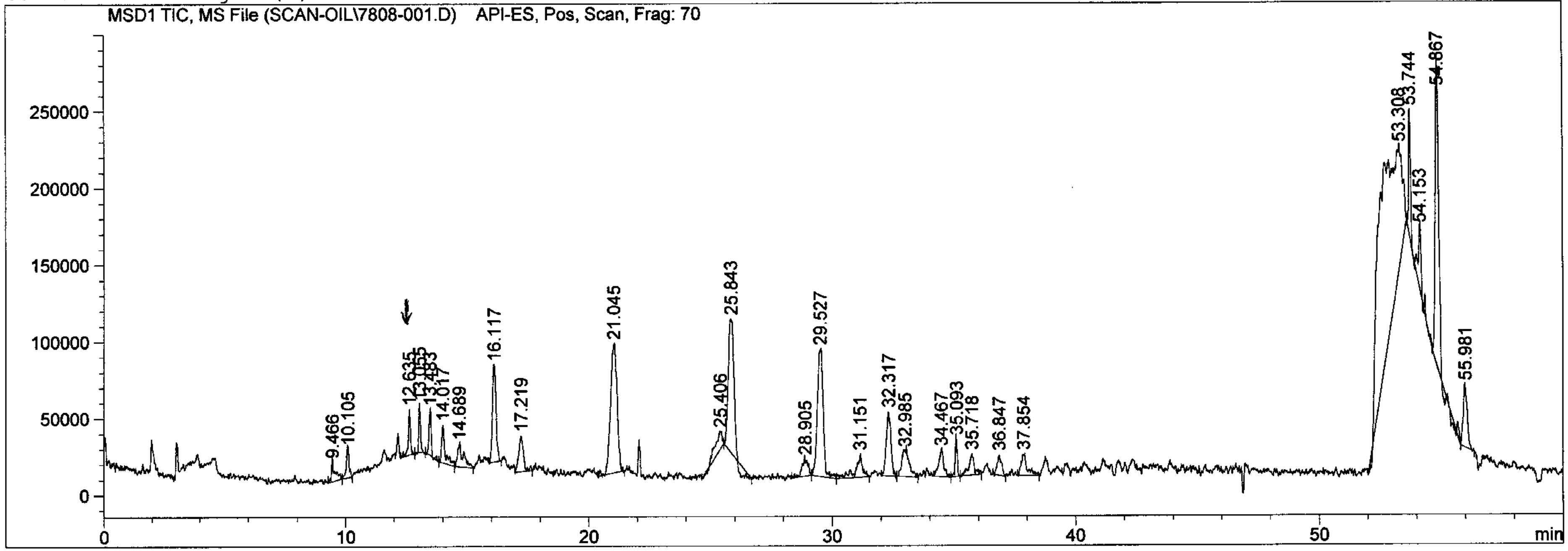

MS Spectrum

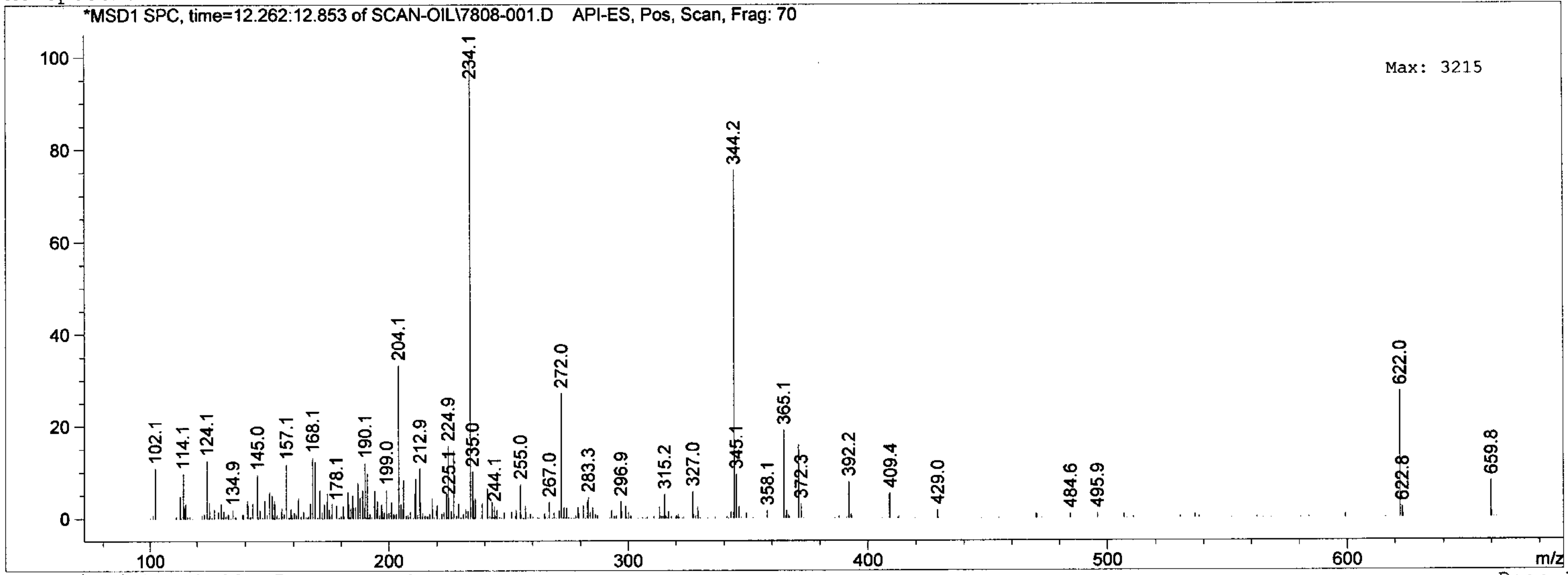

Print of all graphic windows

Current Chromatogram(s)

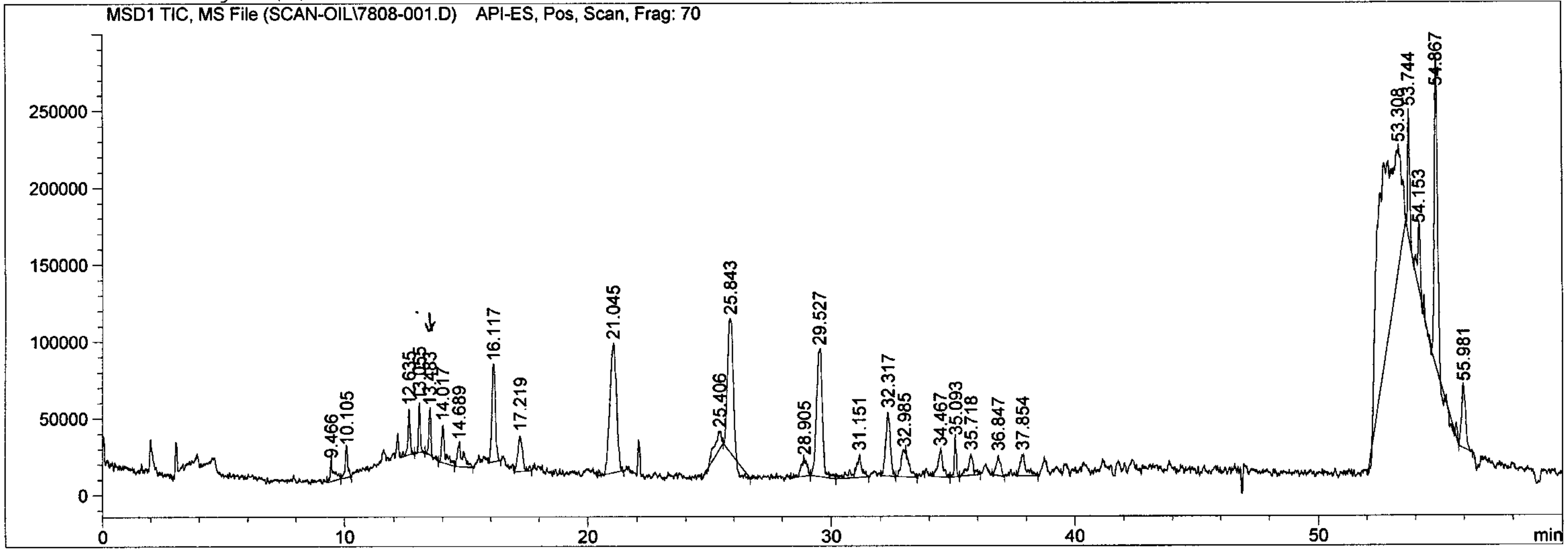

MS Spectrum

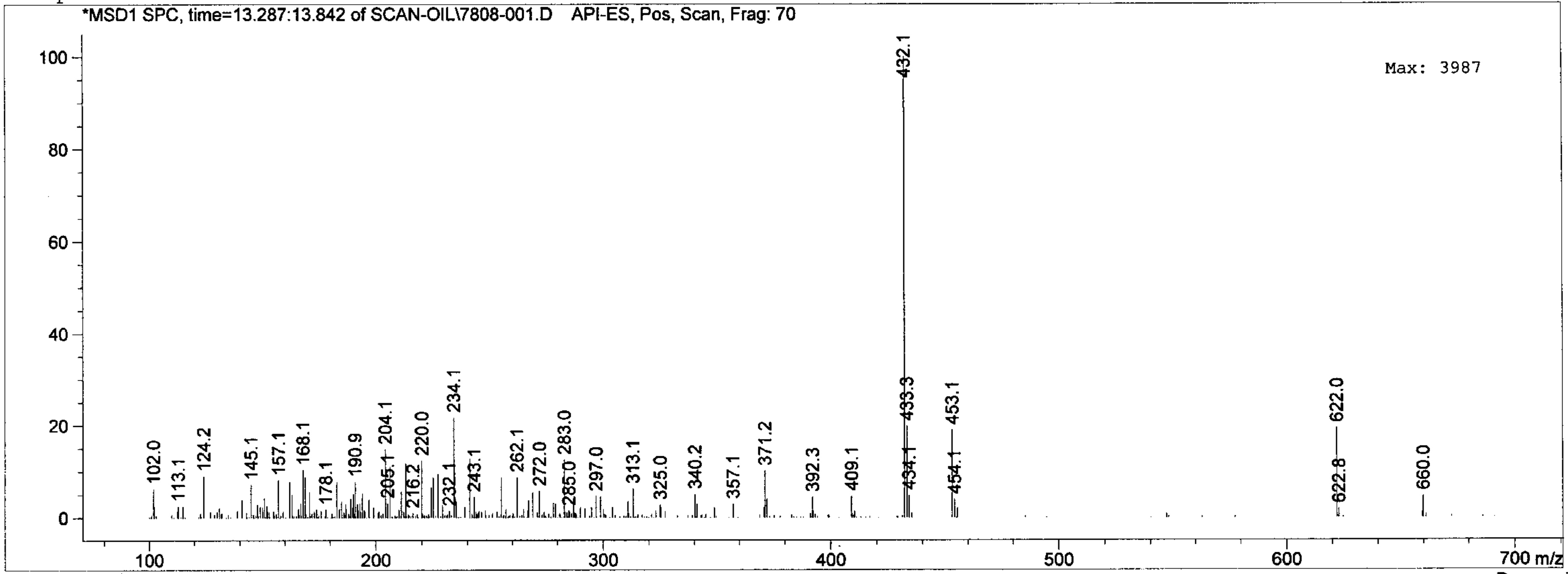

Current Chromatogram(s)

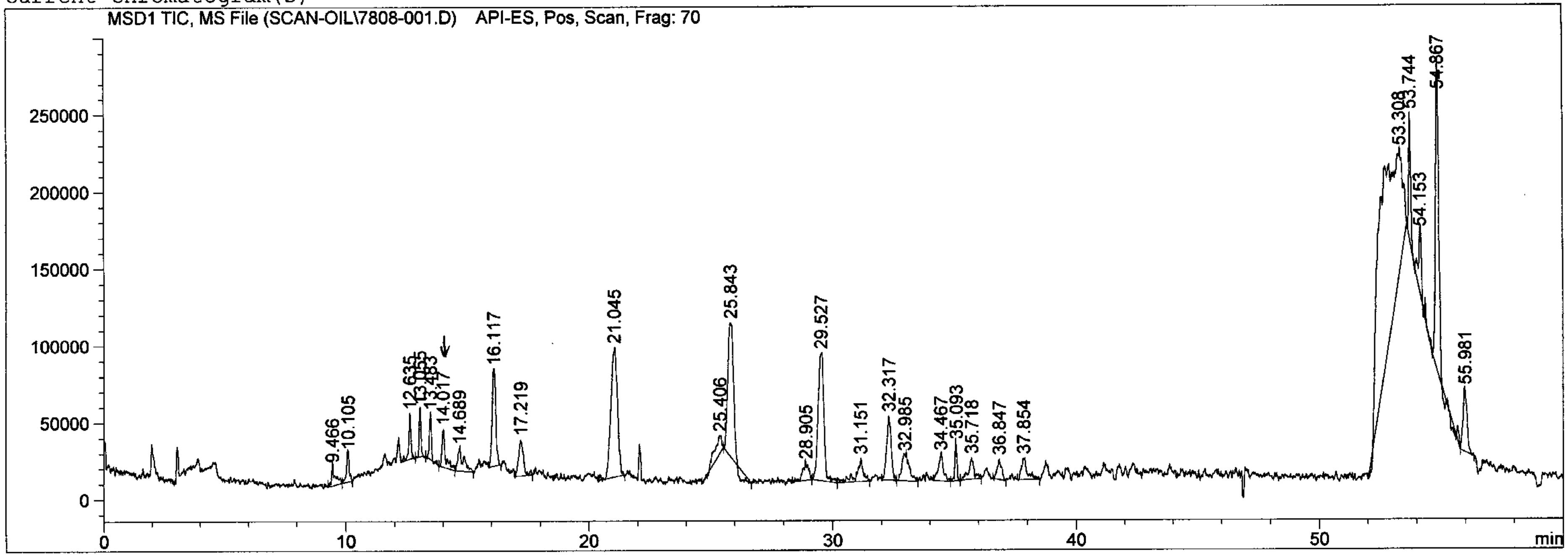

MS Spectrum

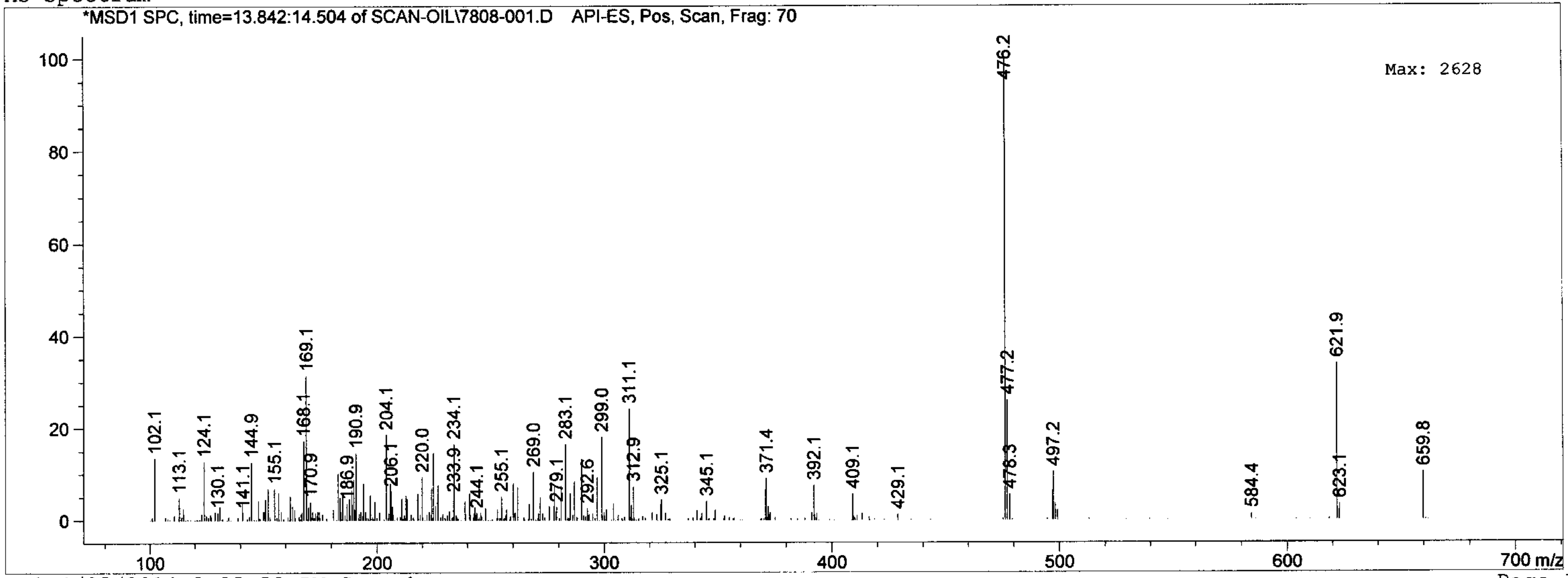

Current Chromatogram(s)

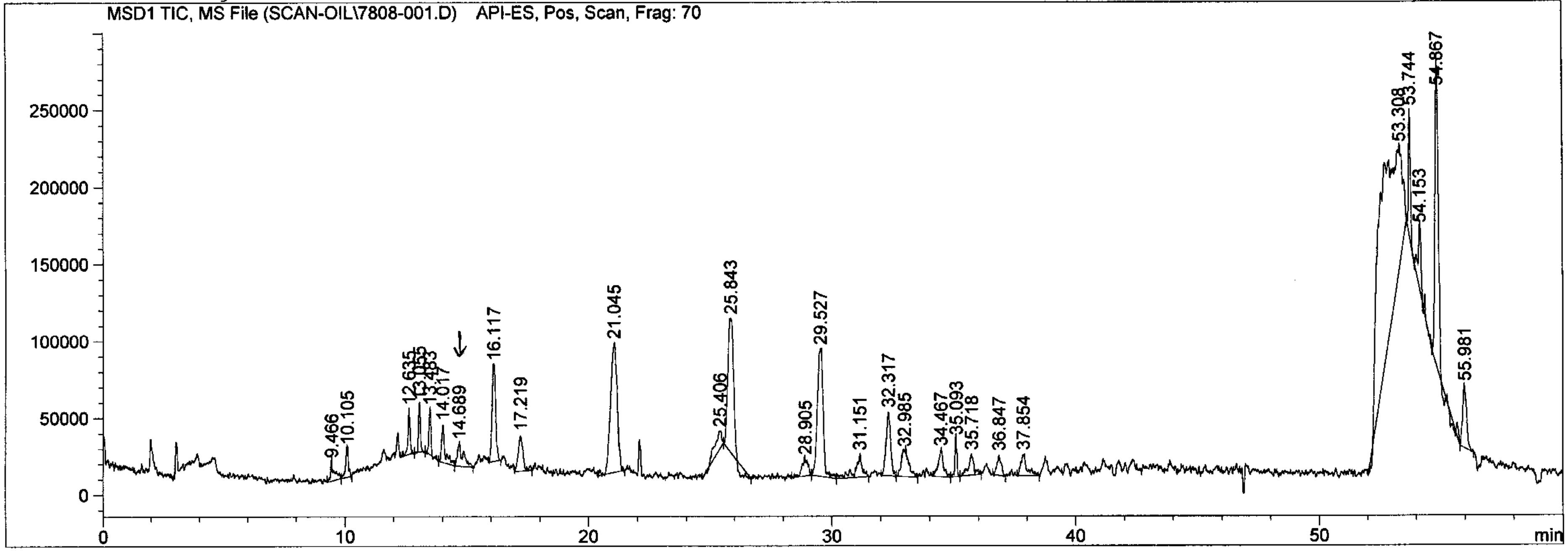

MS Spectrum

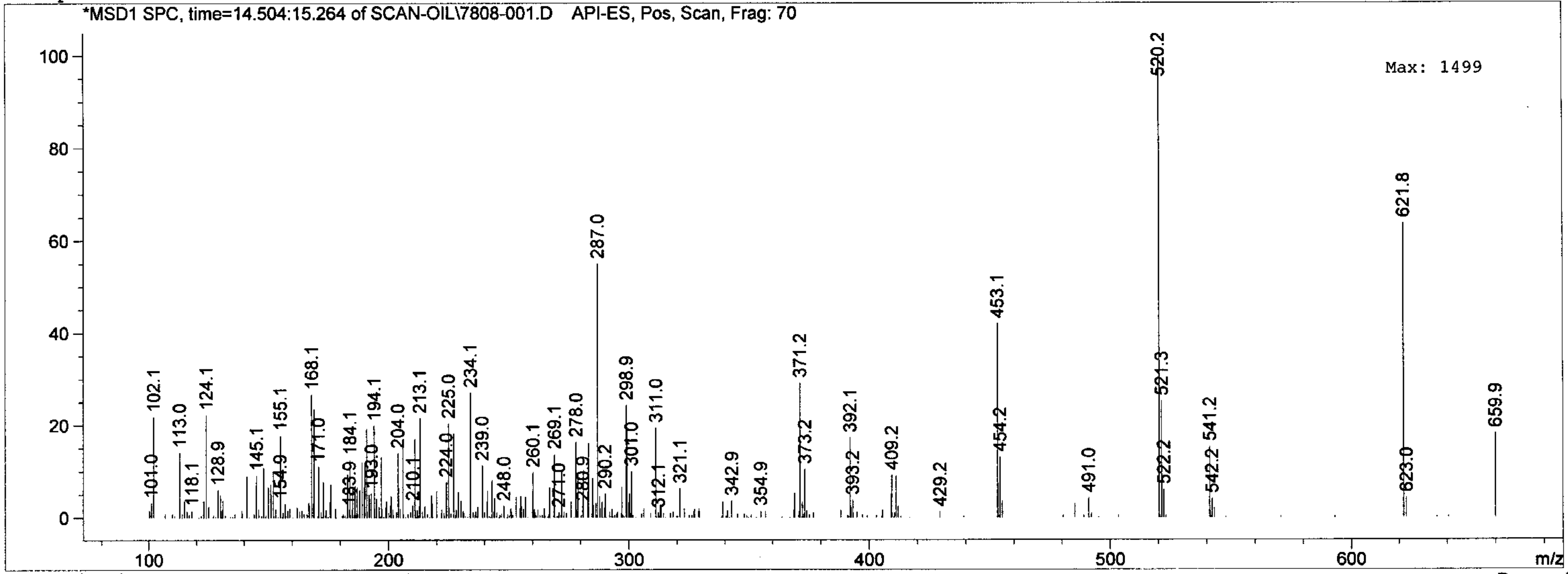

Print of all graphic windows

Current Chromatogram(s)

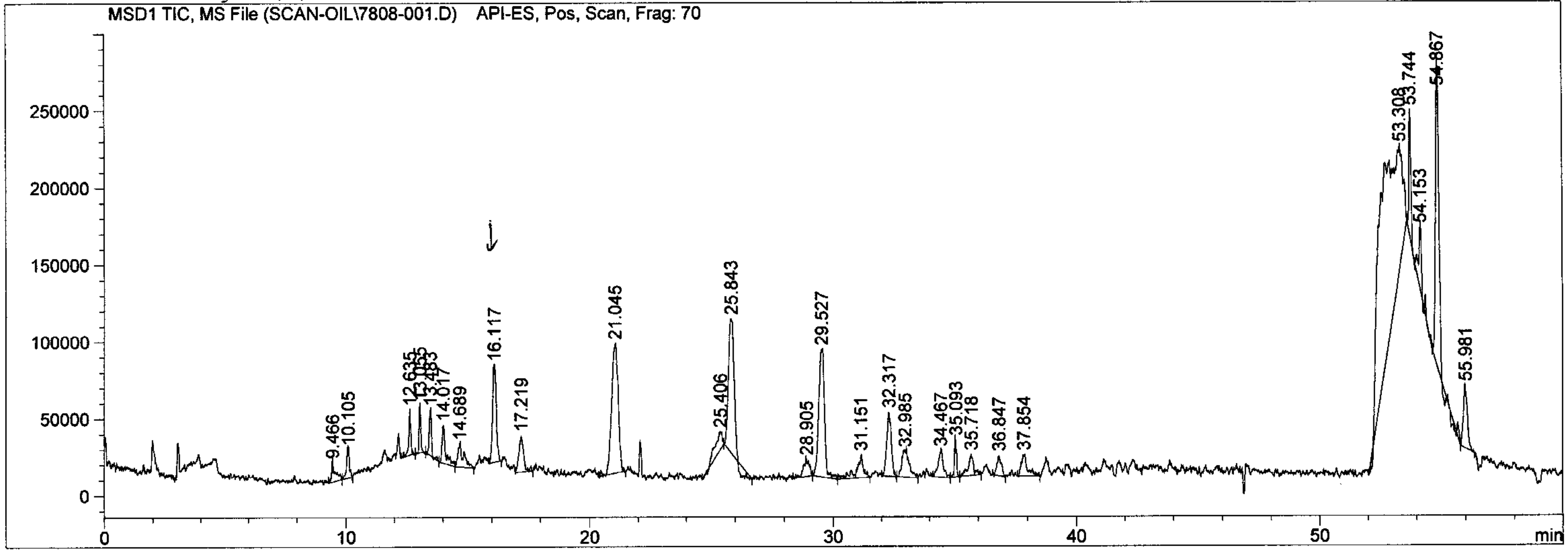

MS Spectrum

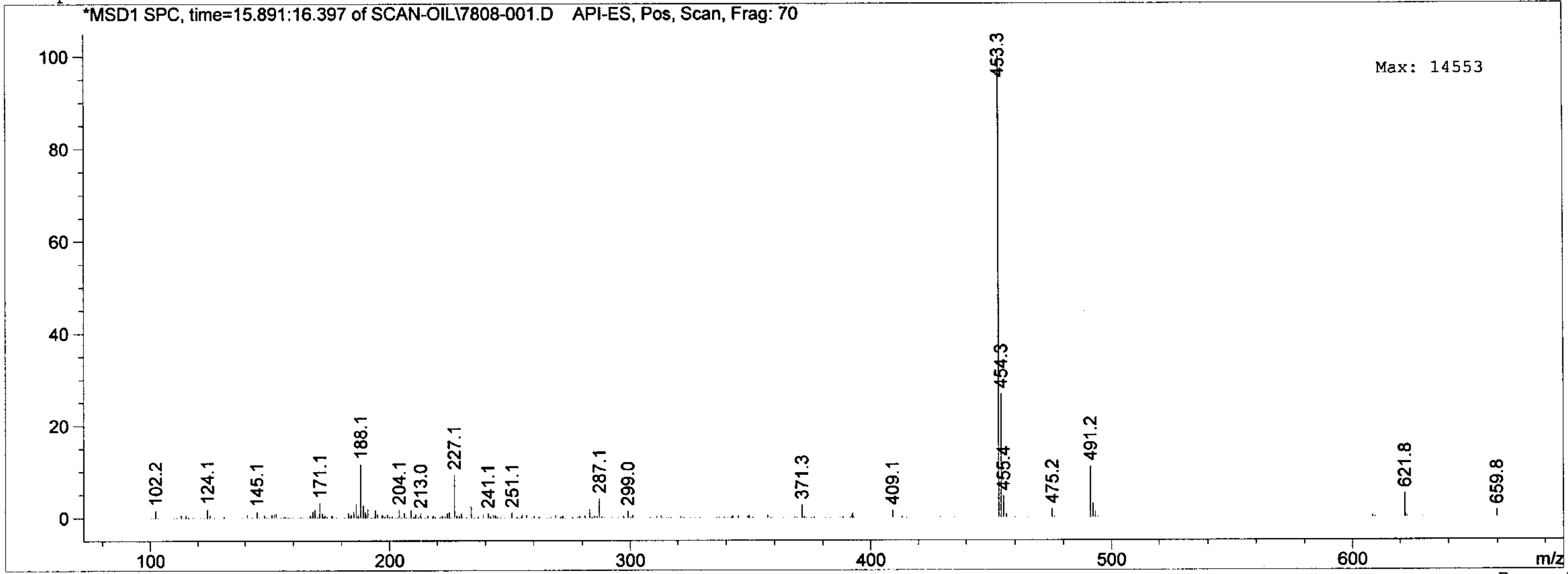

Current Chromatogram(s)

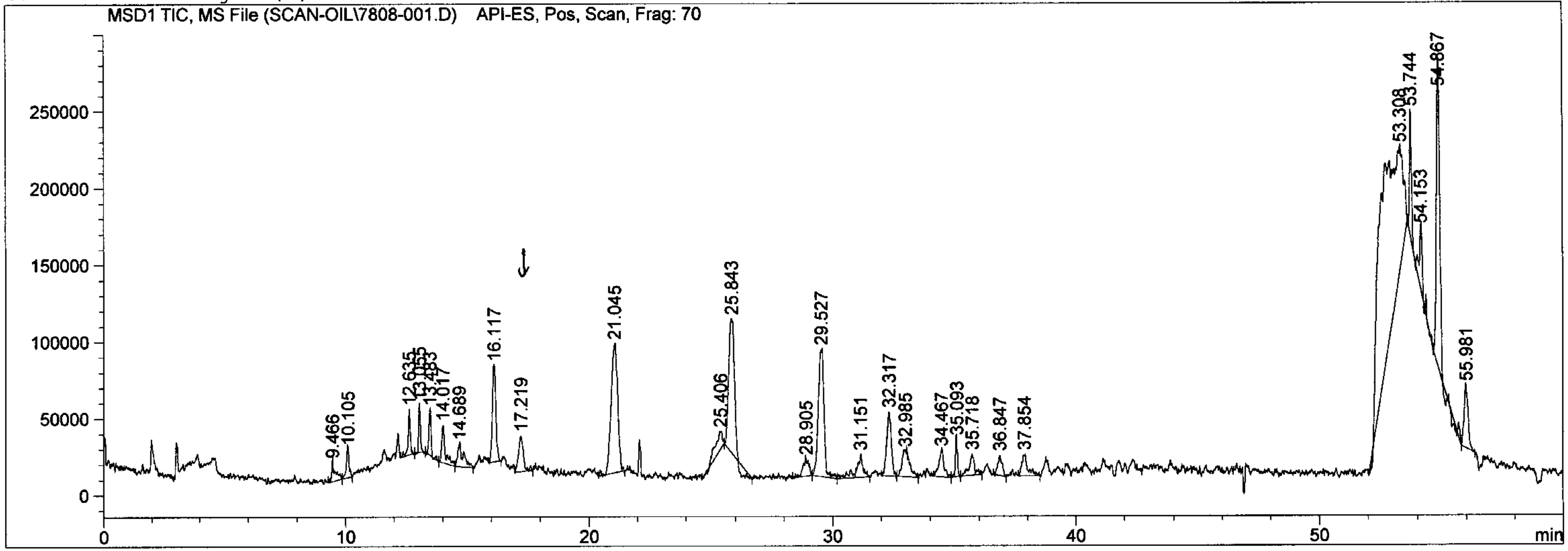

MS Spectrum

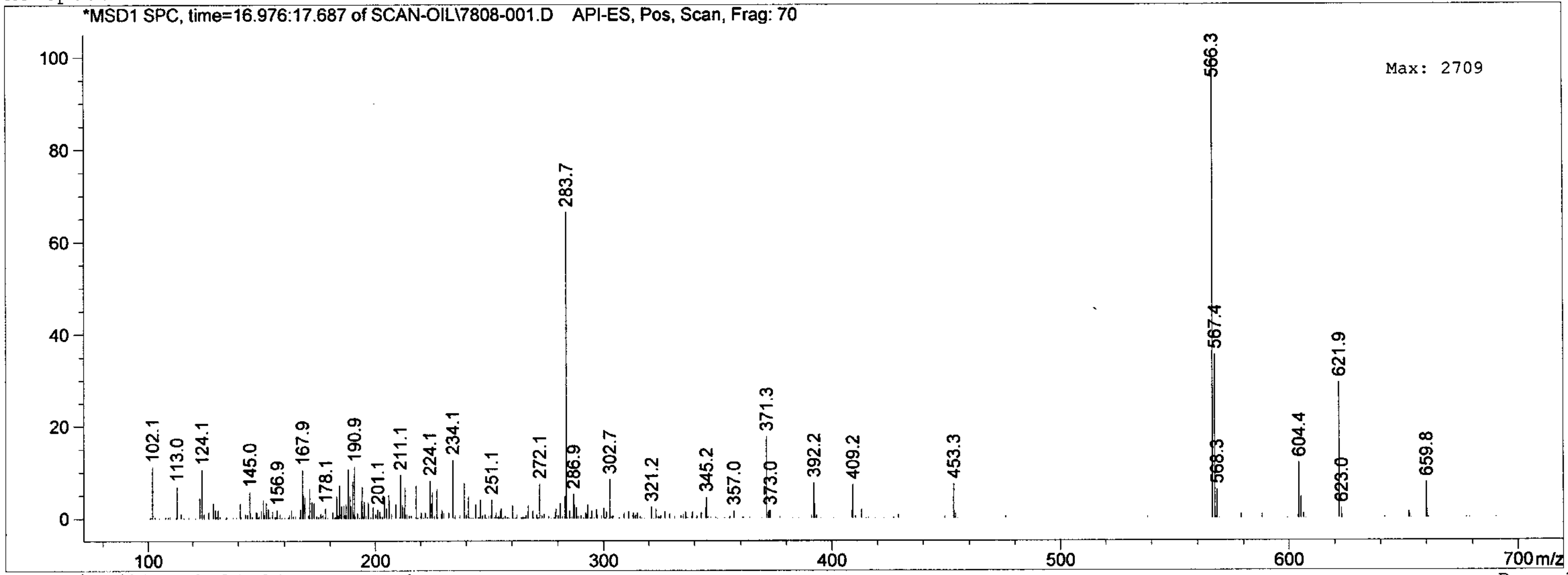

Print of all graphic windows

Current Chromatogram(s)

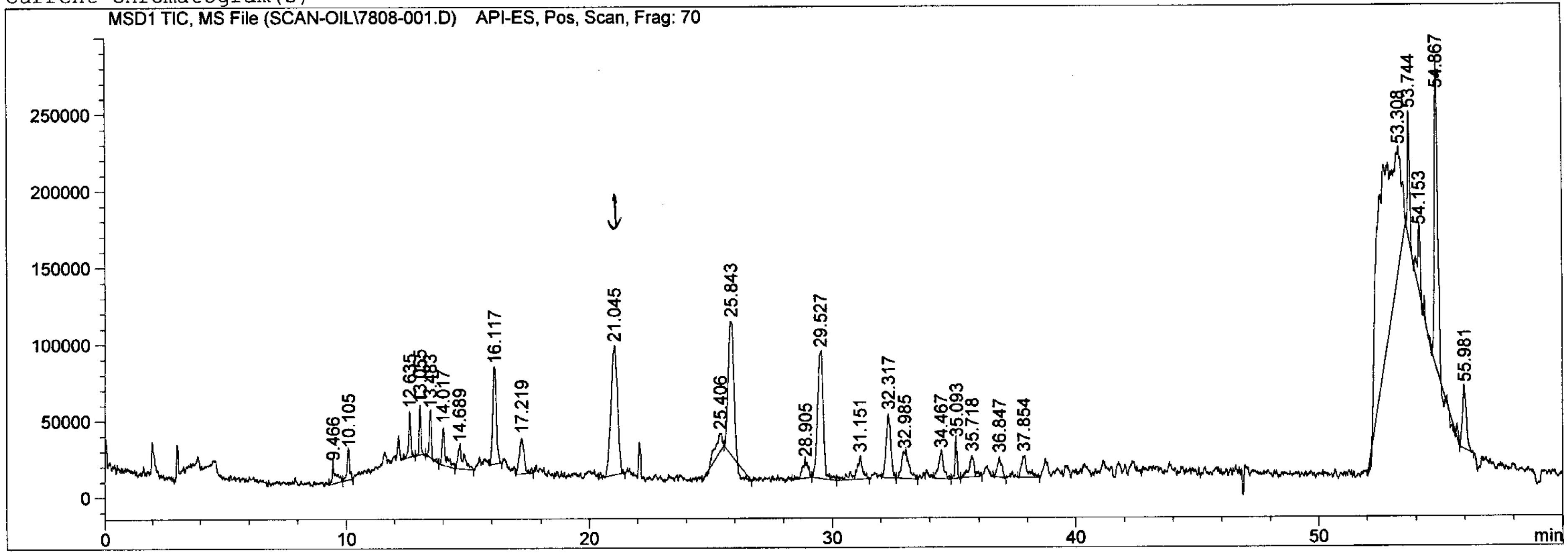

MS Spectrum

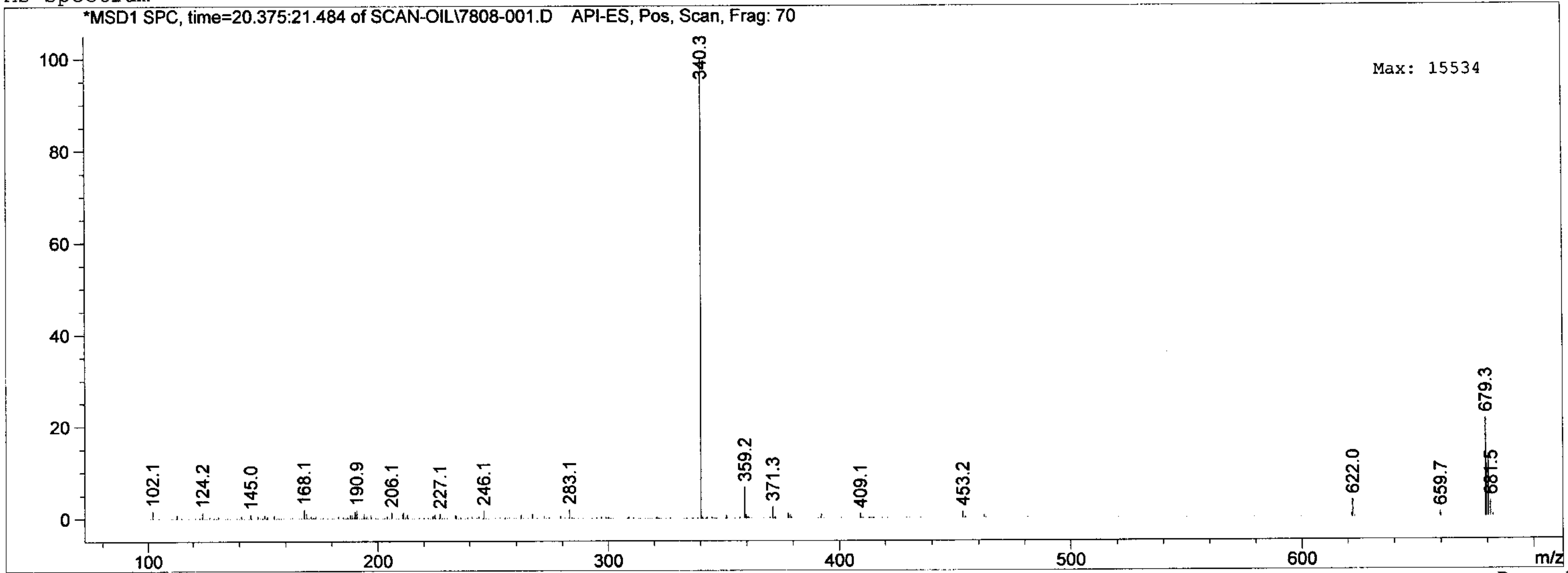

Print of all graphic windows

Current Chromatogram(s)

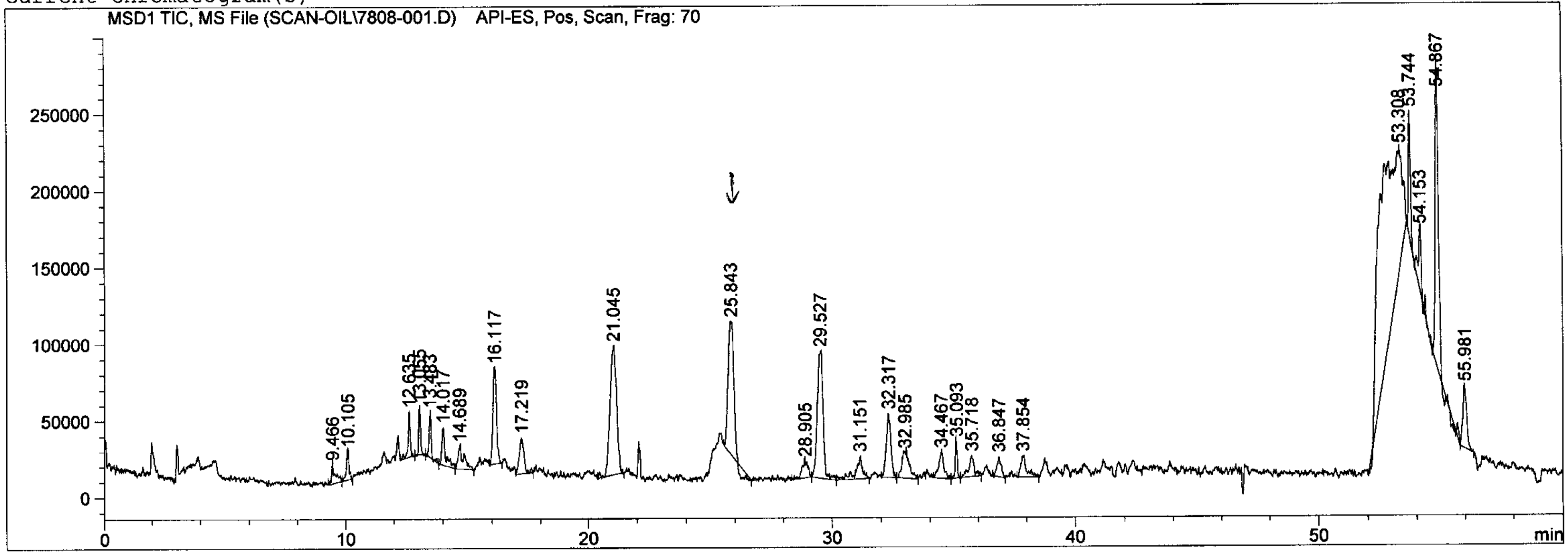

MS Spectrum

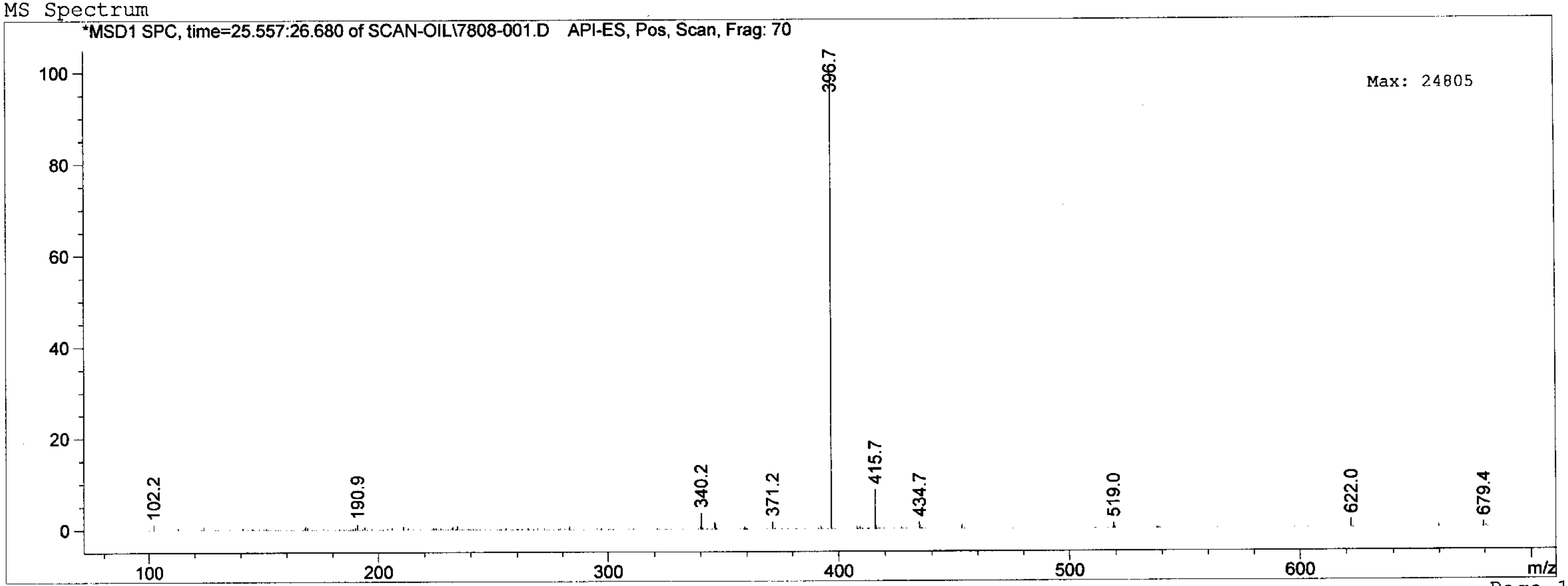

Print of all graphic windows

Current Chromatogram(s)

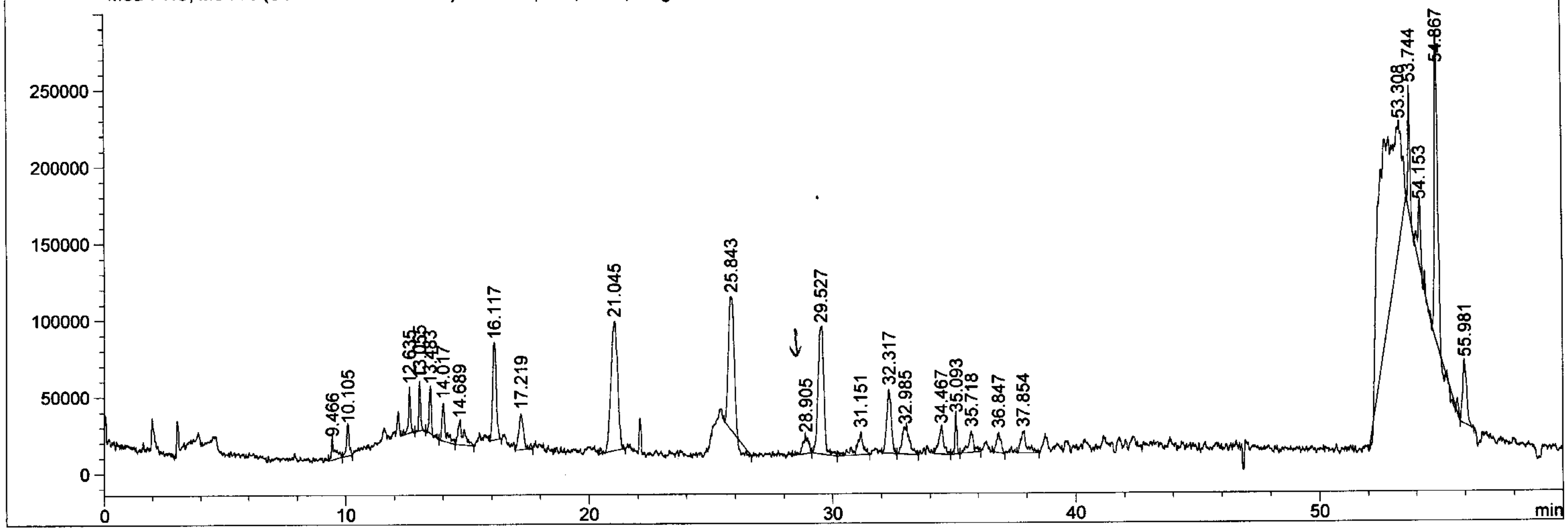

MS Spectrum

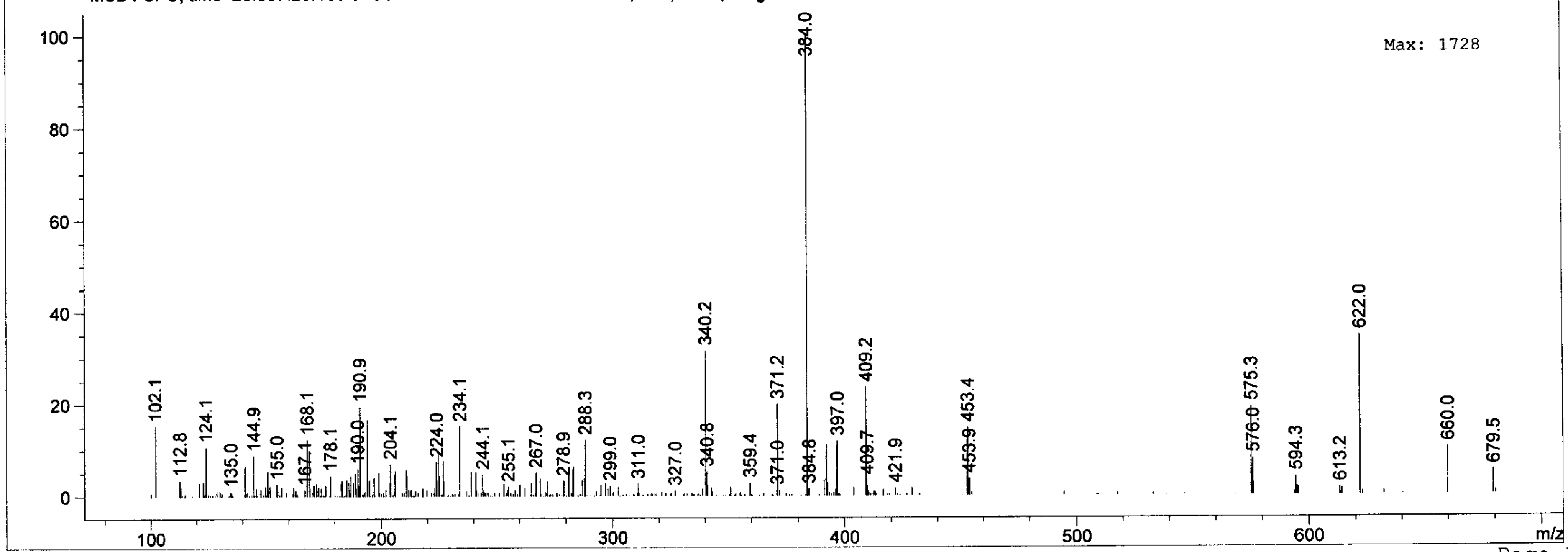

Current Chromatogram(s)

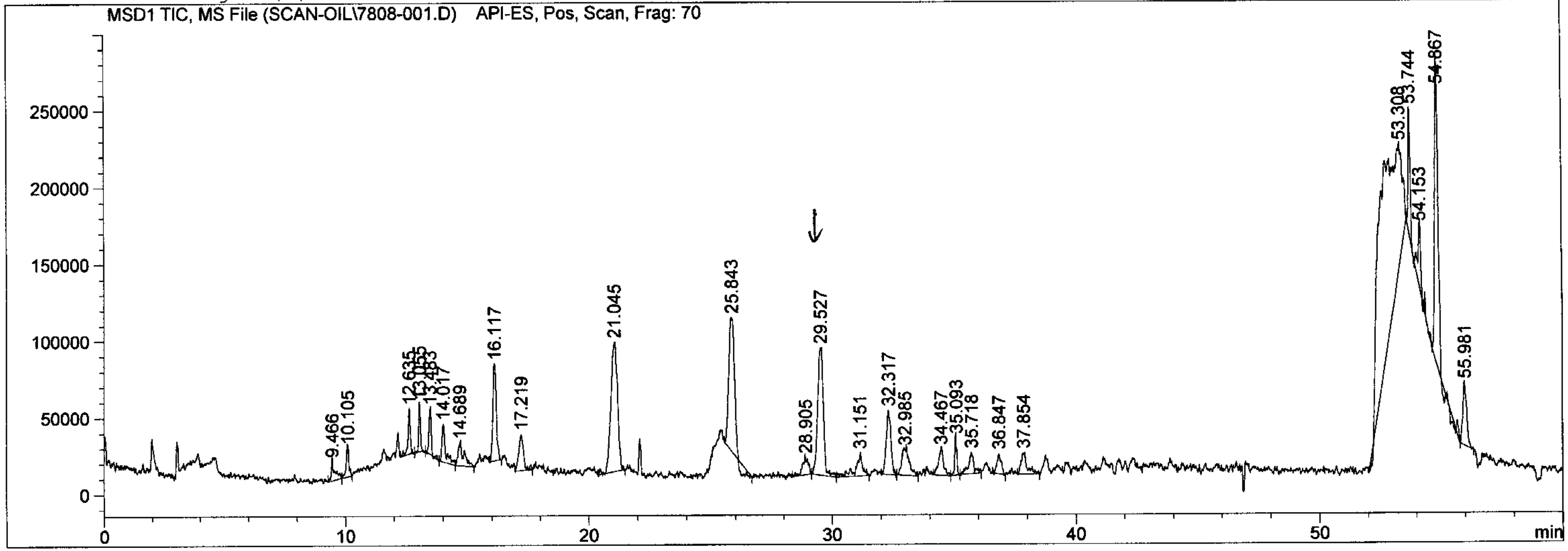

MS Spectrum

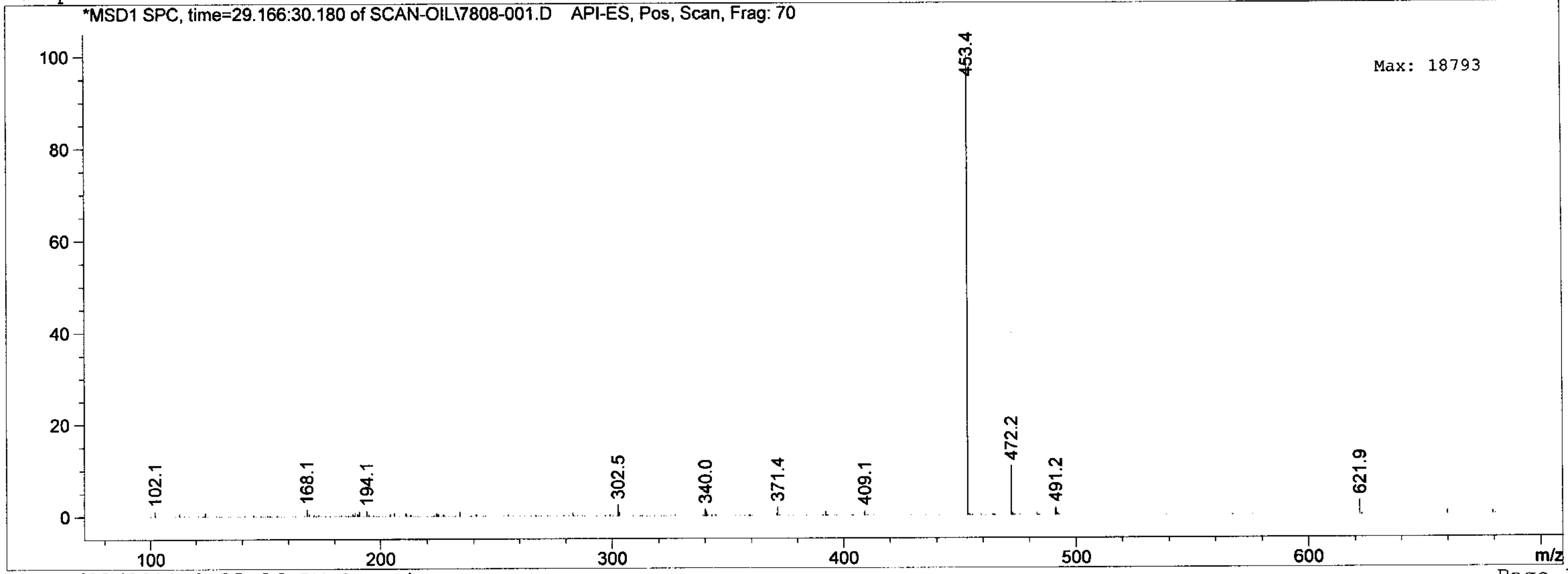

Current Chromatogram(s)

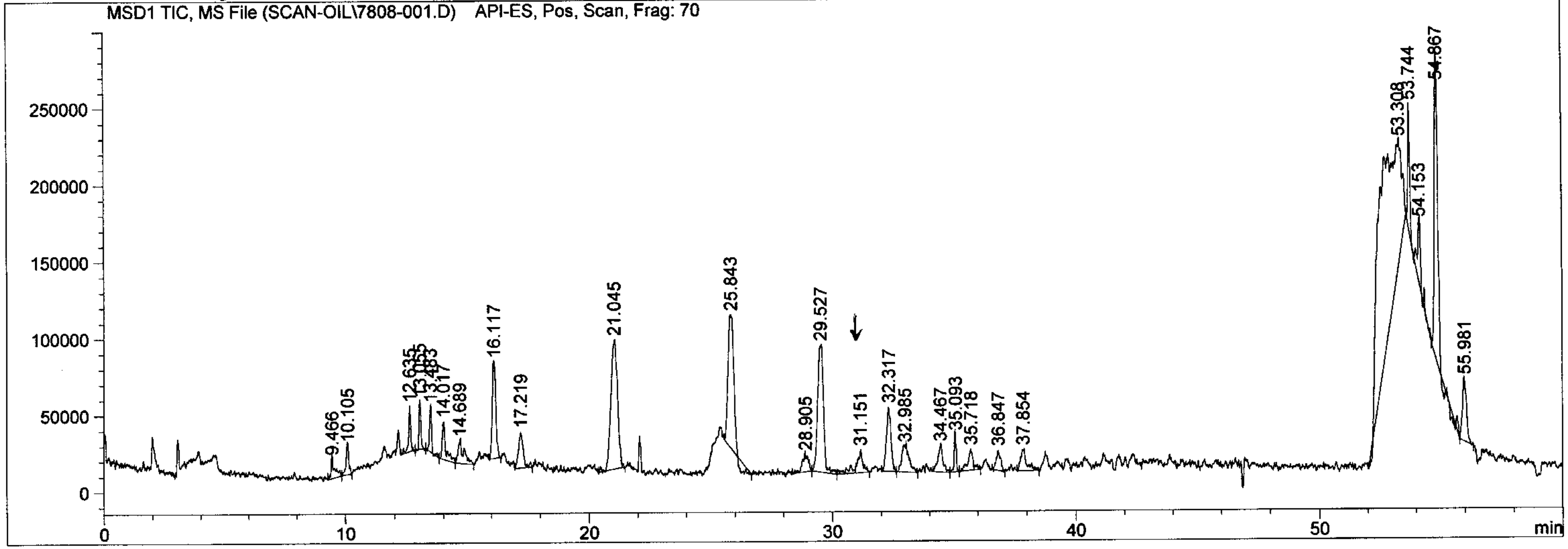

MS Spectrum

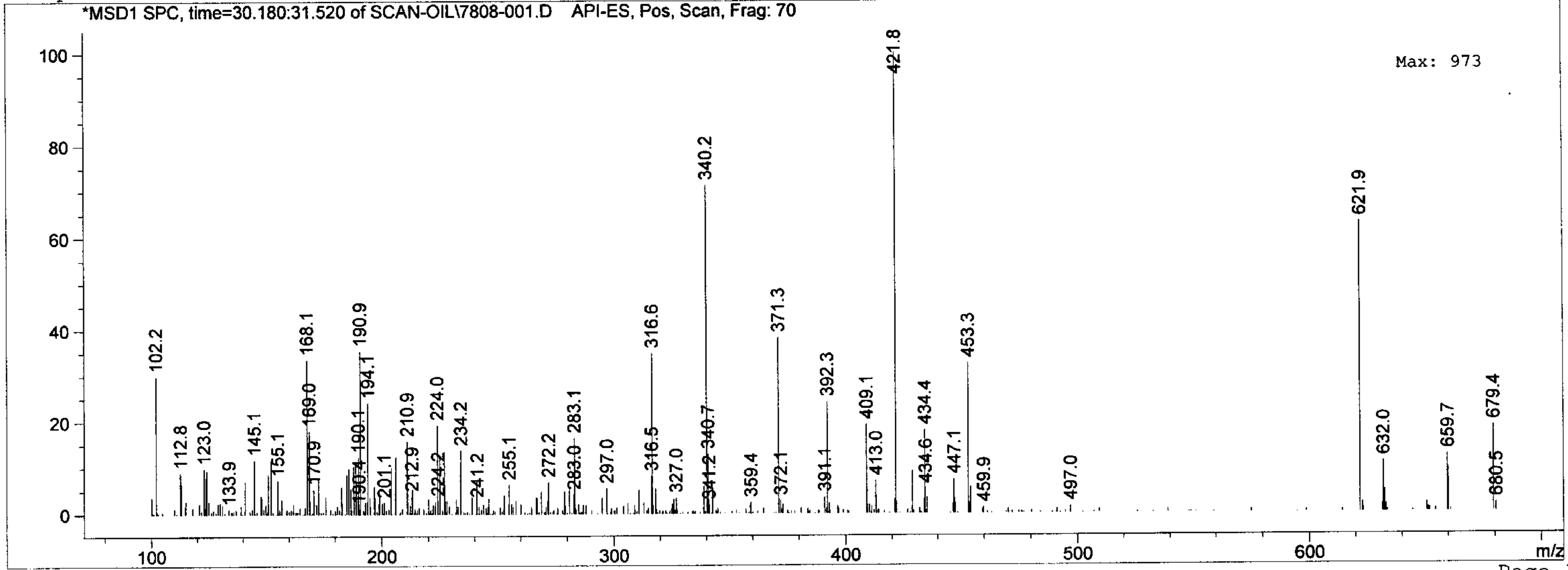

Print of all graphic windows

Current Chromatogram(s)

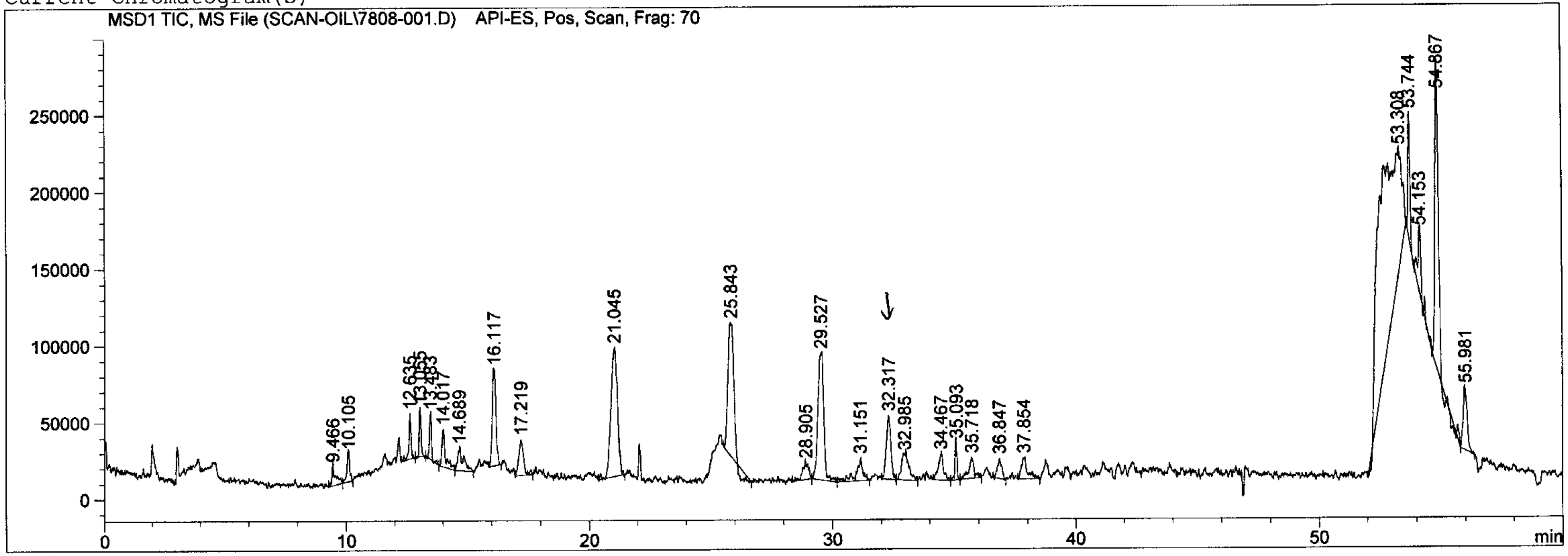

MS Spectrum

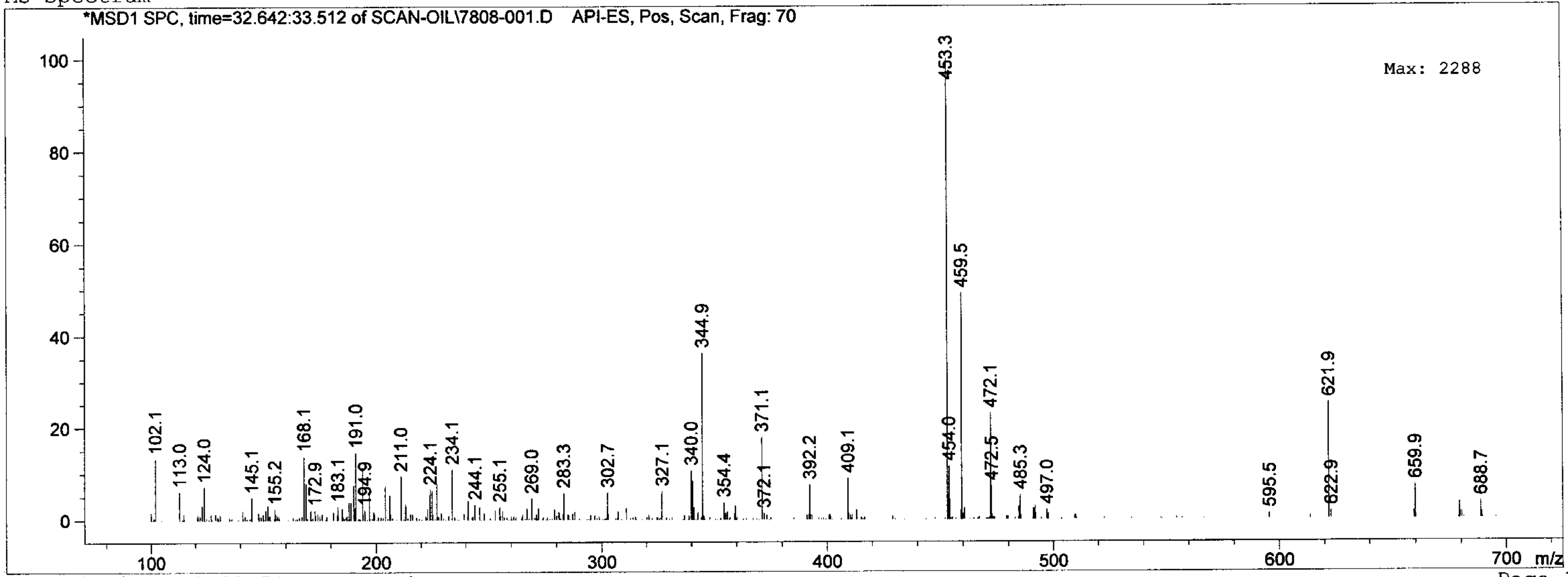

Print of all graphic windows

Current Chromatogram(s)

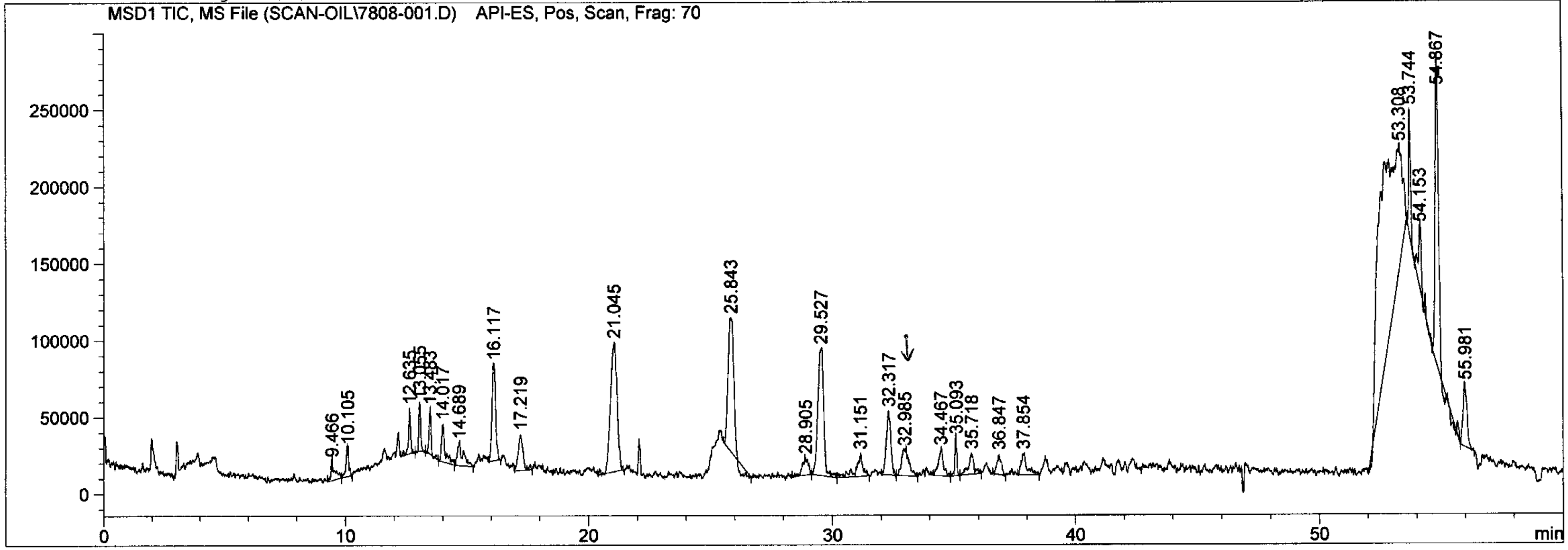

MS Spectrum

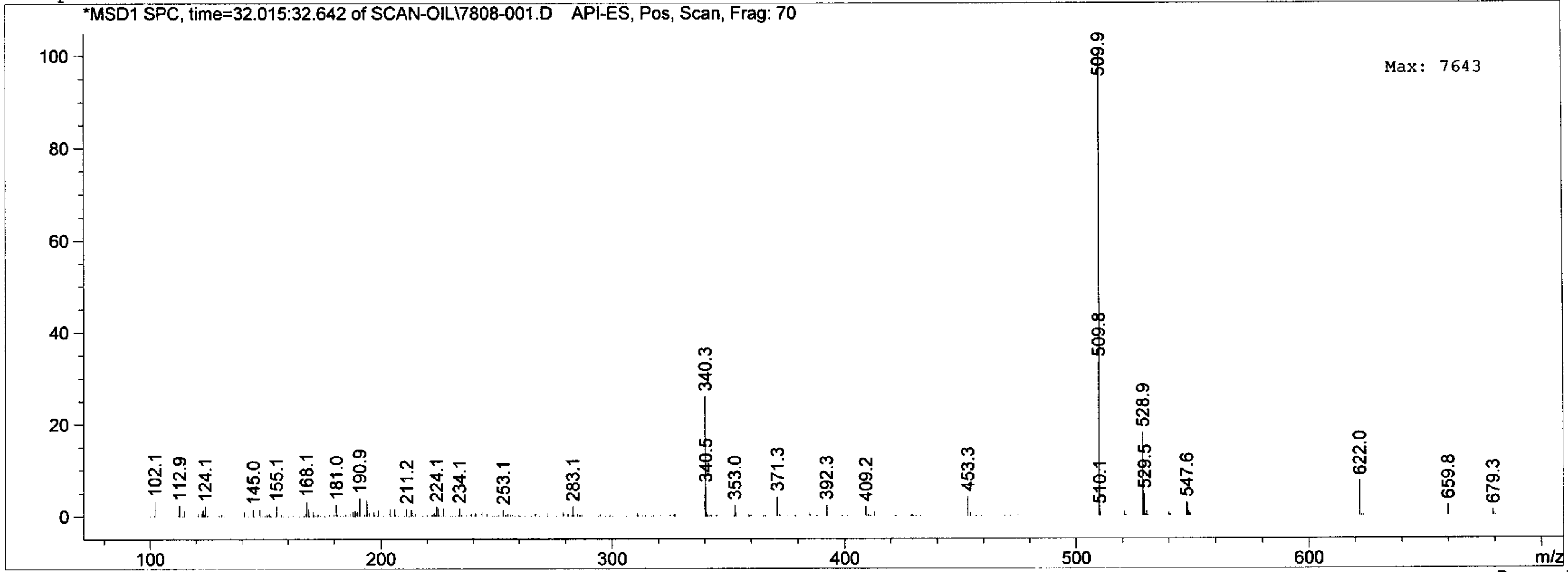

Print of all graphic windows

Current Chromatogram(s)

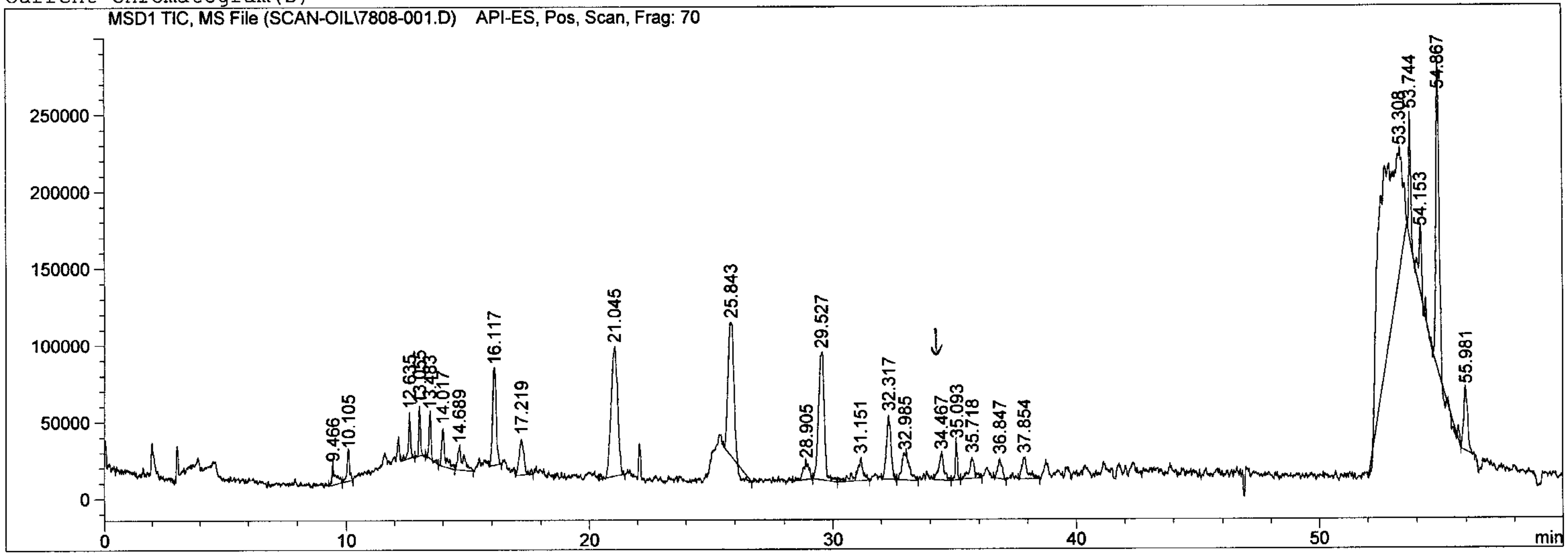

MS Spectrum

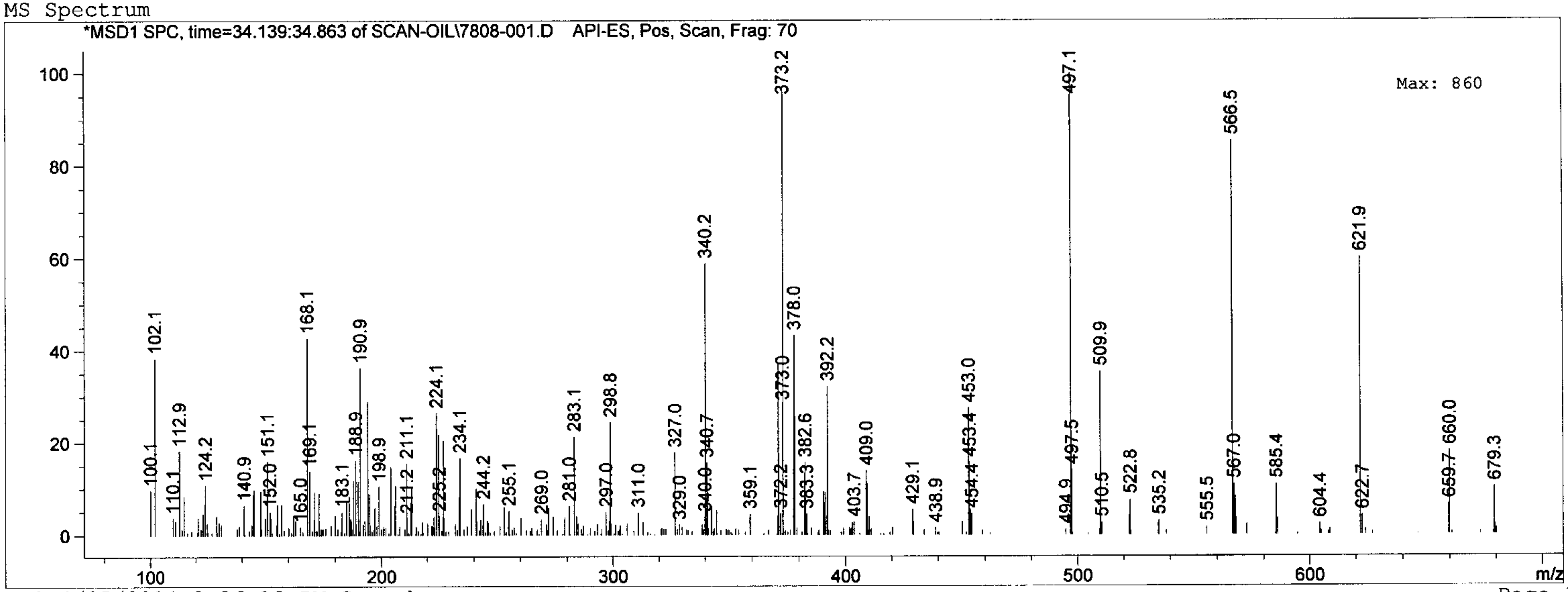

Instrument 1 4/25/2014 2:26:10 PM Somsak

Current Chromatogram(s)

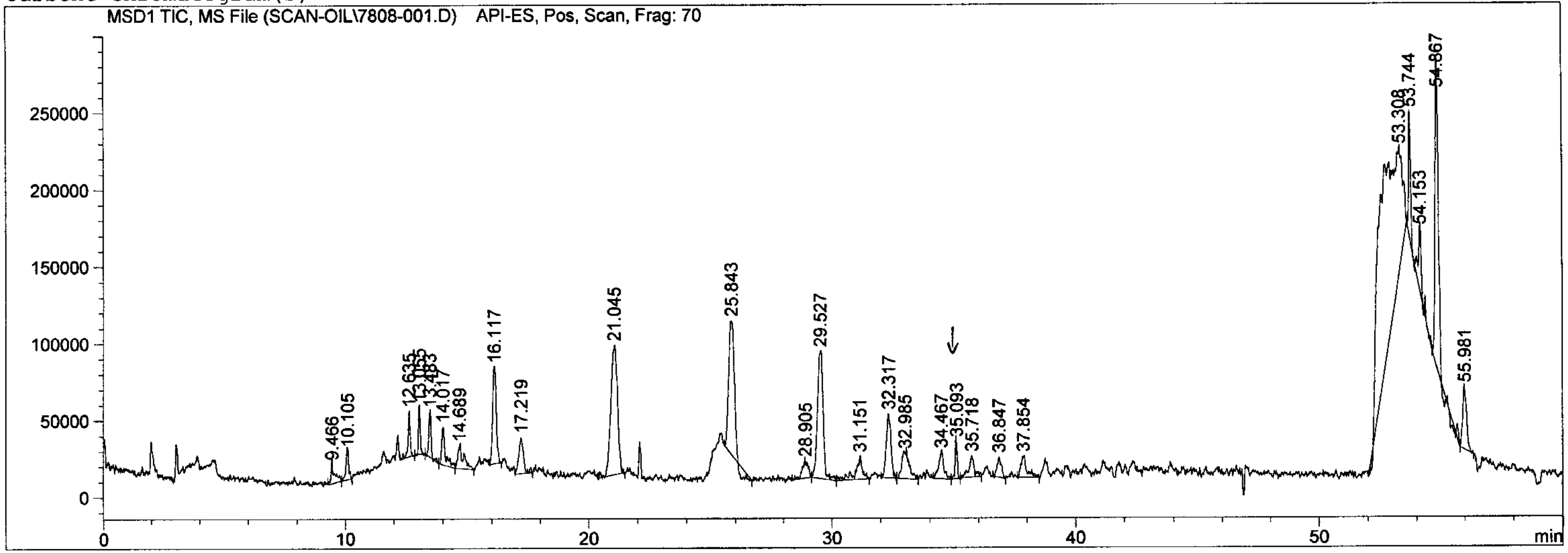

MS Spectrum

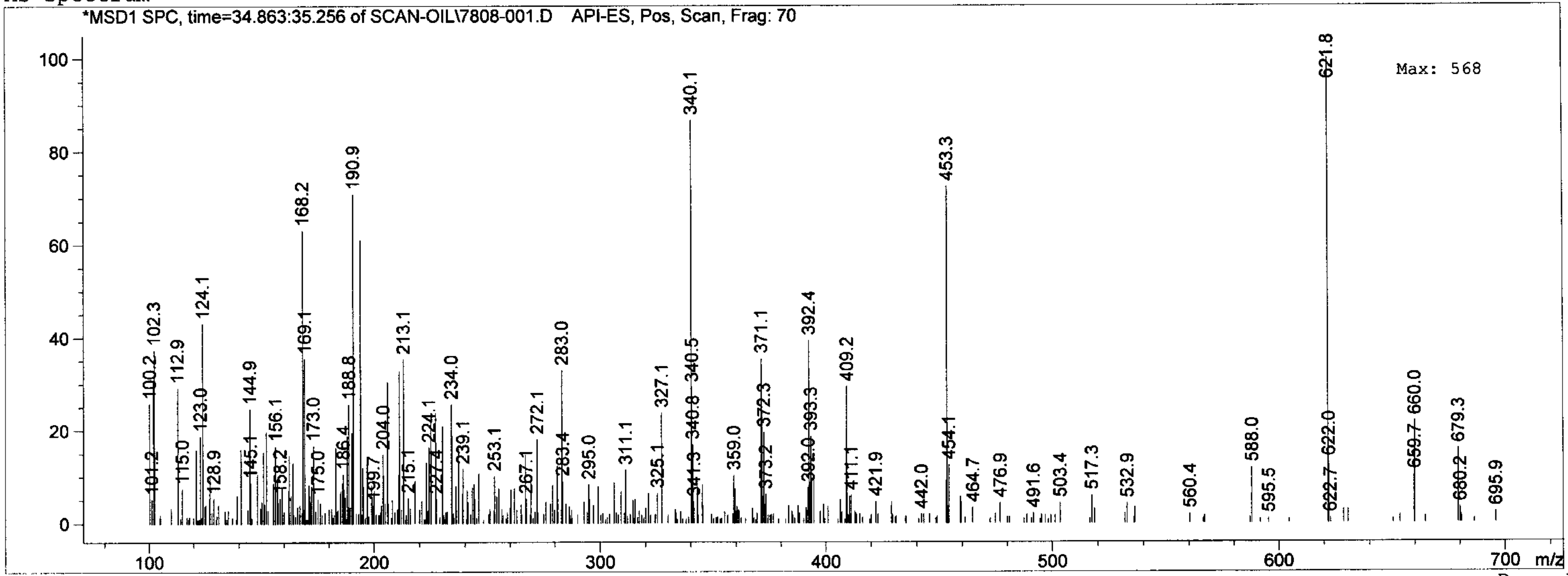

Print of all graphic windows

Current Chromatogram(s)

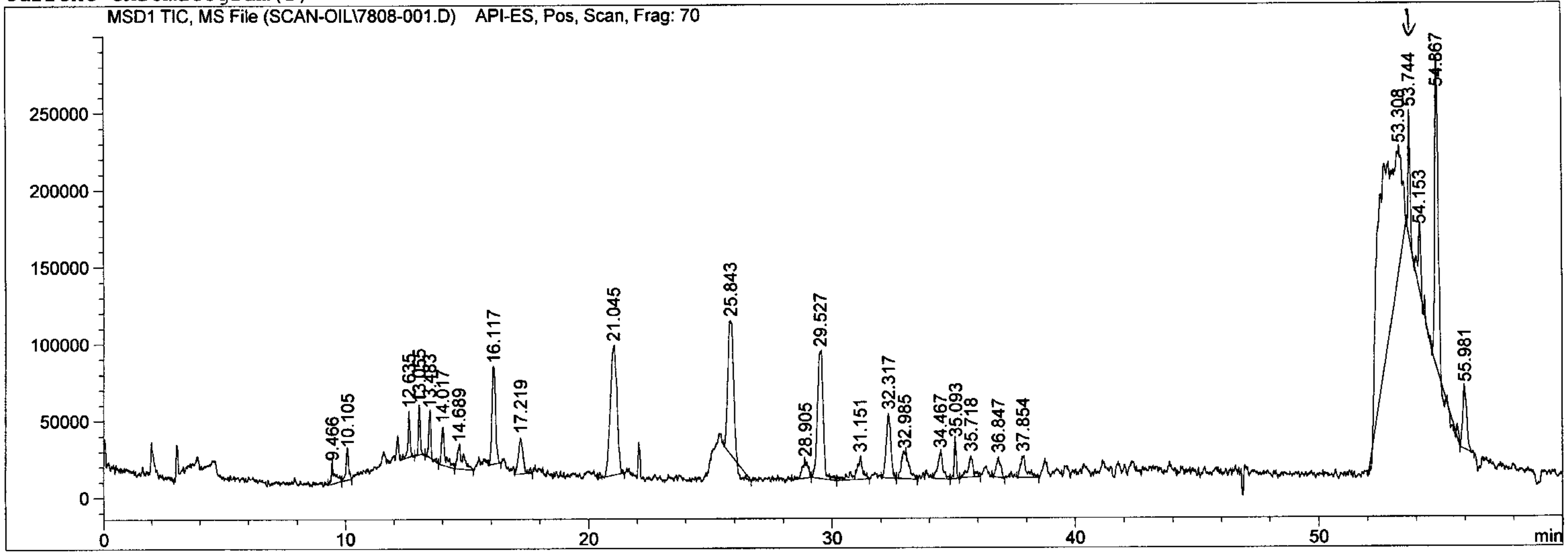

MS Spectrum

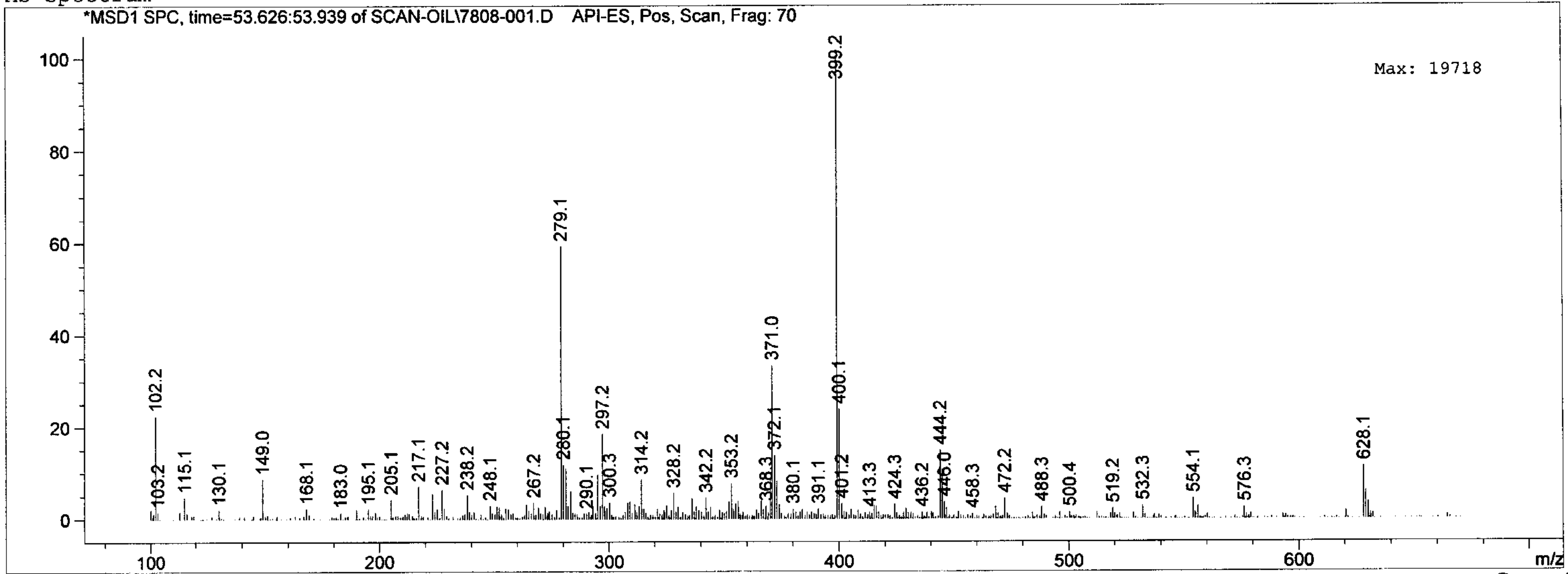

Instrument 1 4/25/2014 2:26:48 PM Somsak

Current Chromatogram(s)

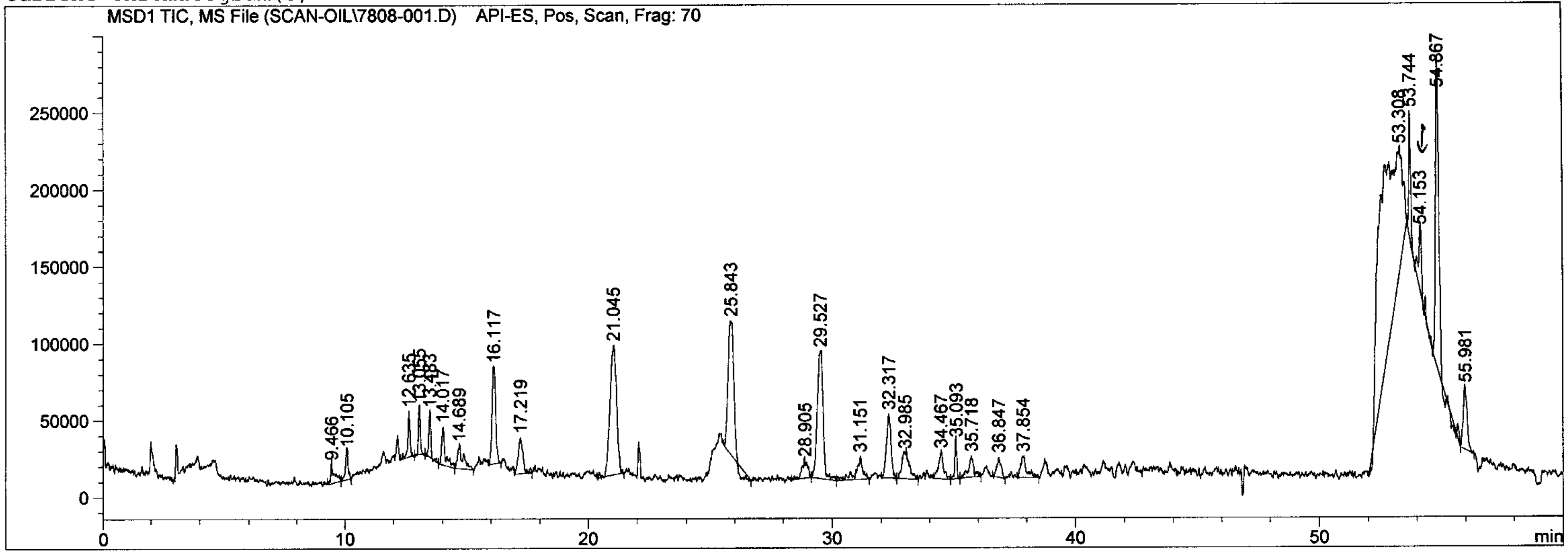

MS Spectrum

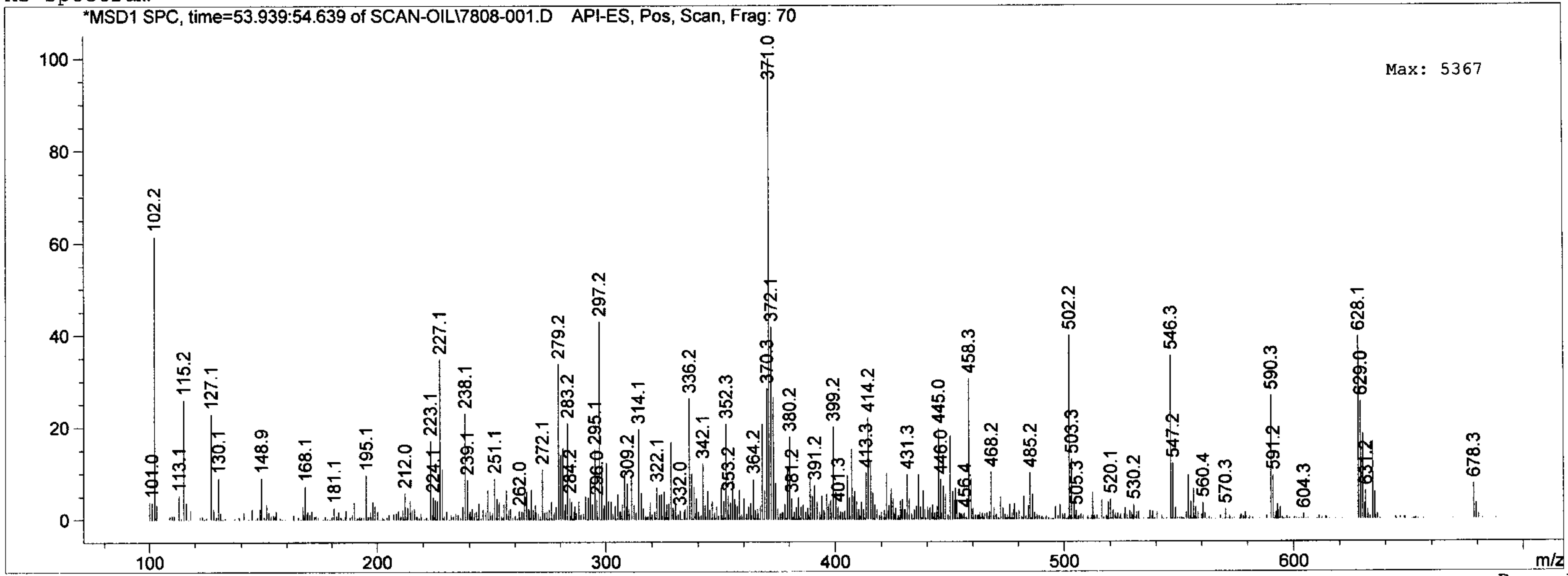

Print of all graphic windows

Current Chromatogram(s)

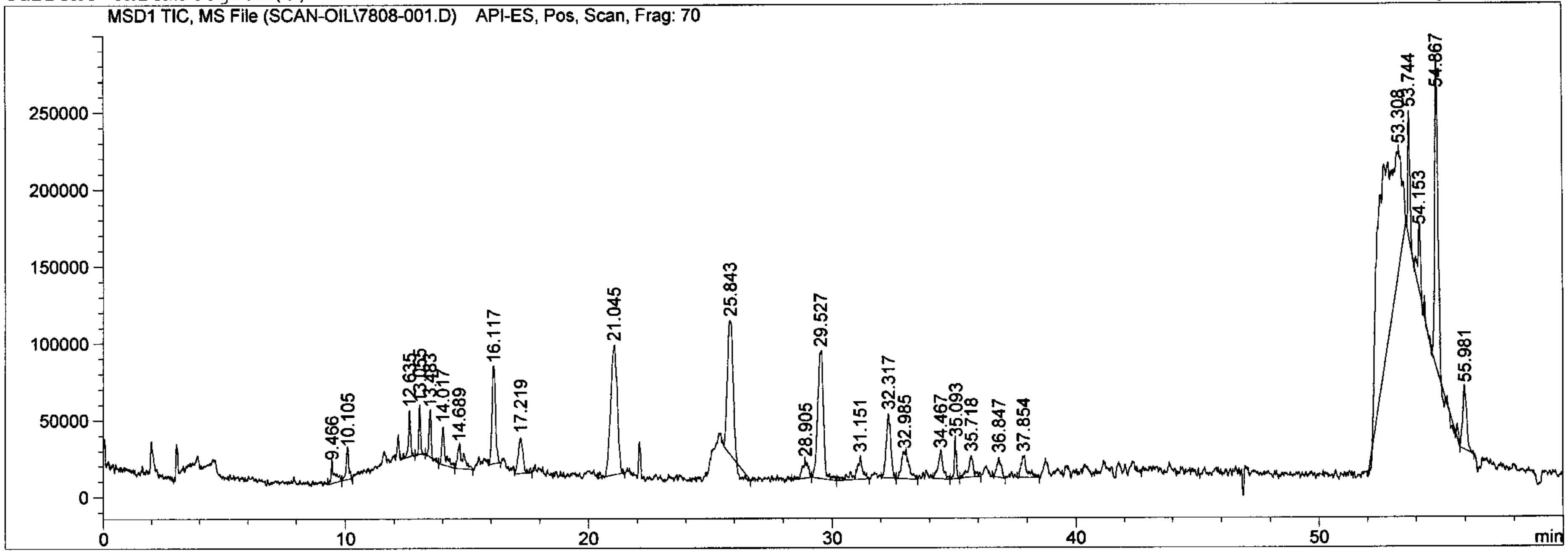

MS Spectrum

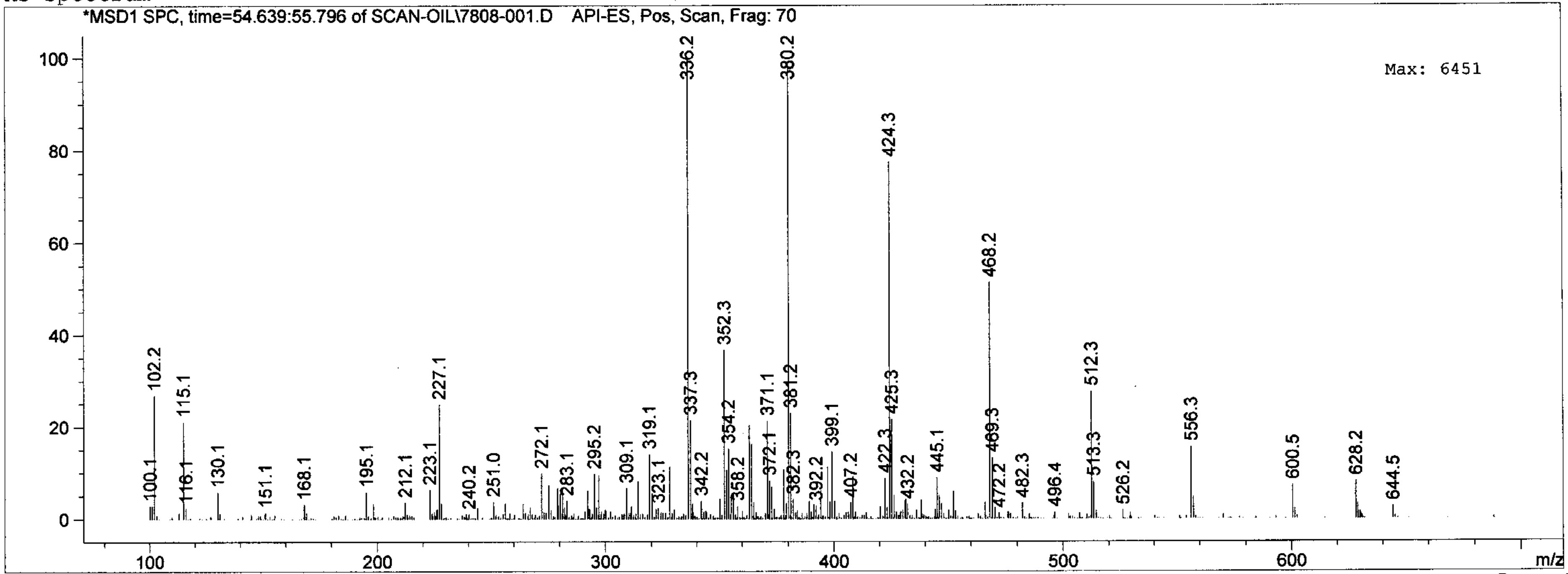

Print of all graphic windows

Current Chromatogram(s)

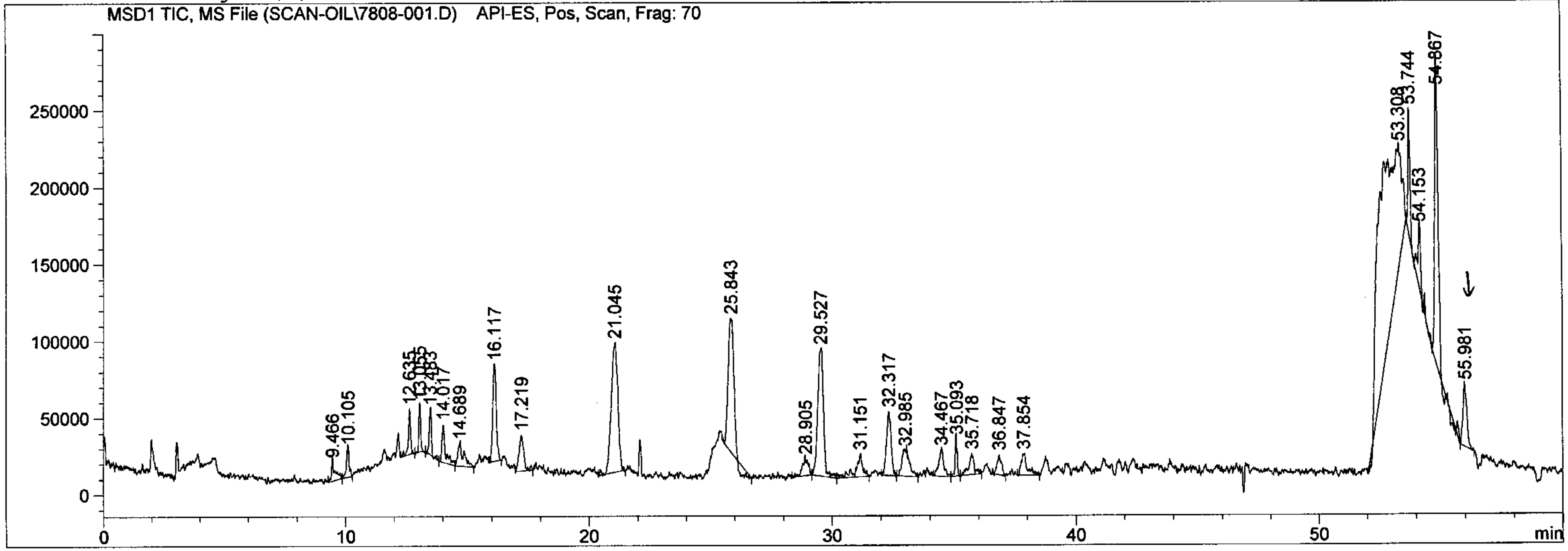

MS Spectrum

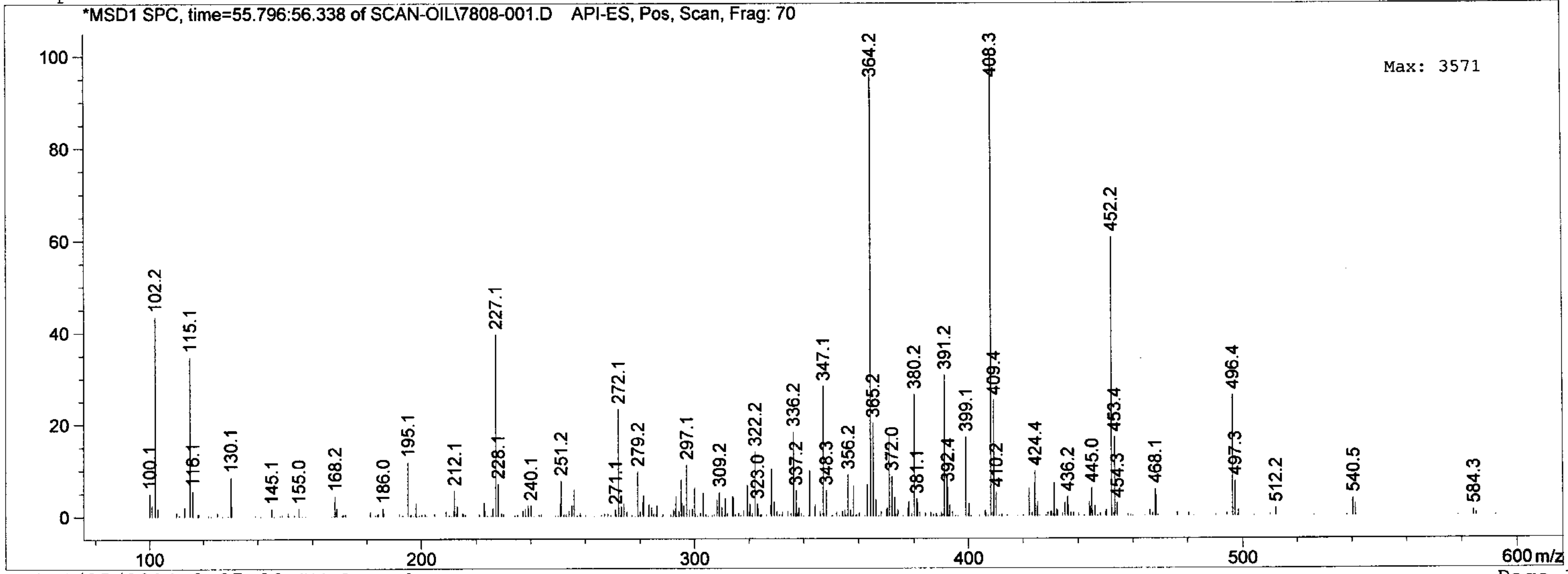

Instrument 1 4/25/2014 2:27:20 PM Somsak
