# Supplementary material for: Phytosterol, Lipid and Phenolic Composition, and Biological Activities of Guava Seed Oil
Source: Molecules. 2020 May 27;25(11):2474. doi: 10.3390/molecules25112474 (PMC7321134; doi:10.3390/molecules25112474)

Current Chromatogram(s)

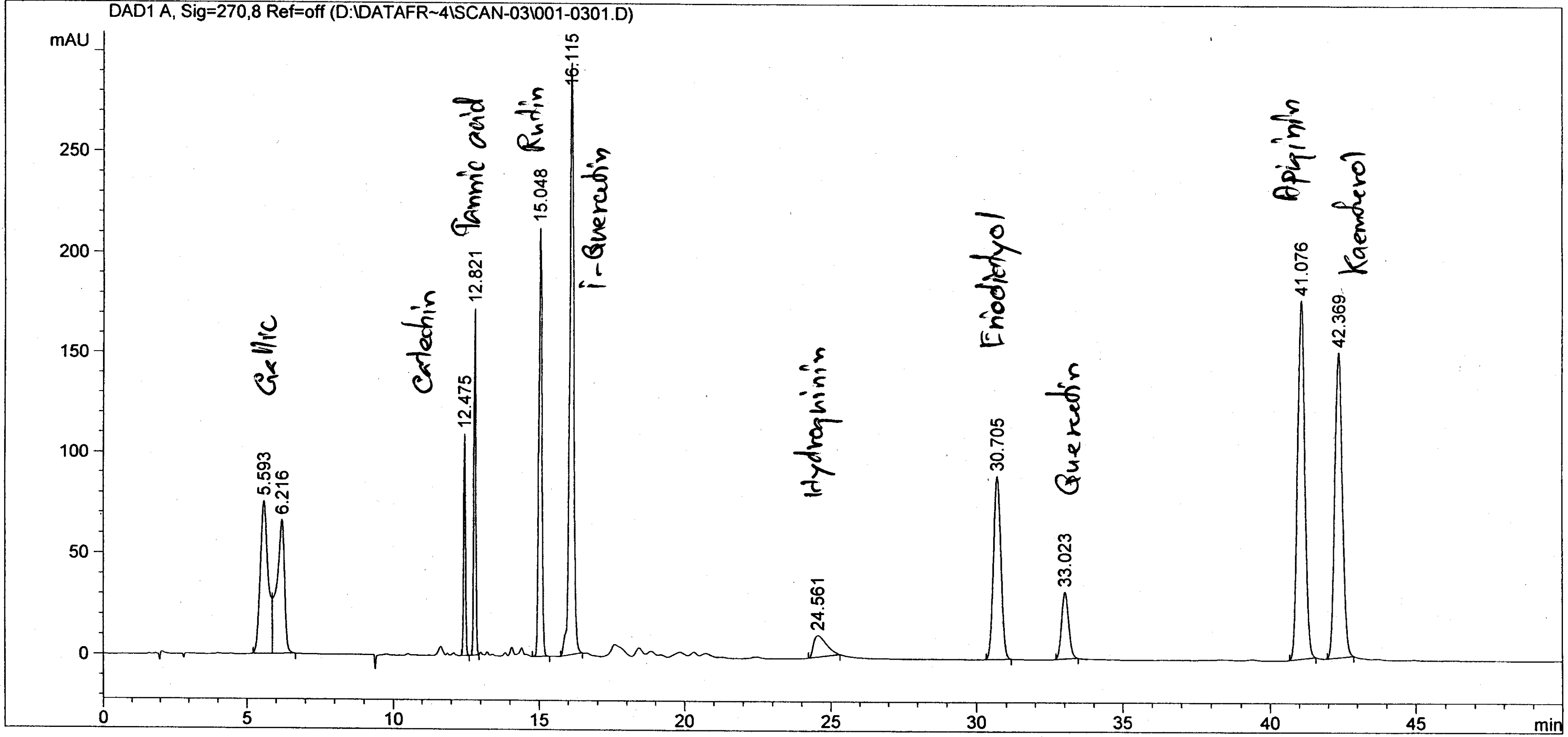

Print of all graphic windows

Gallic â

Current Chromatogram(s)

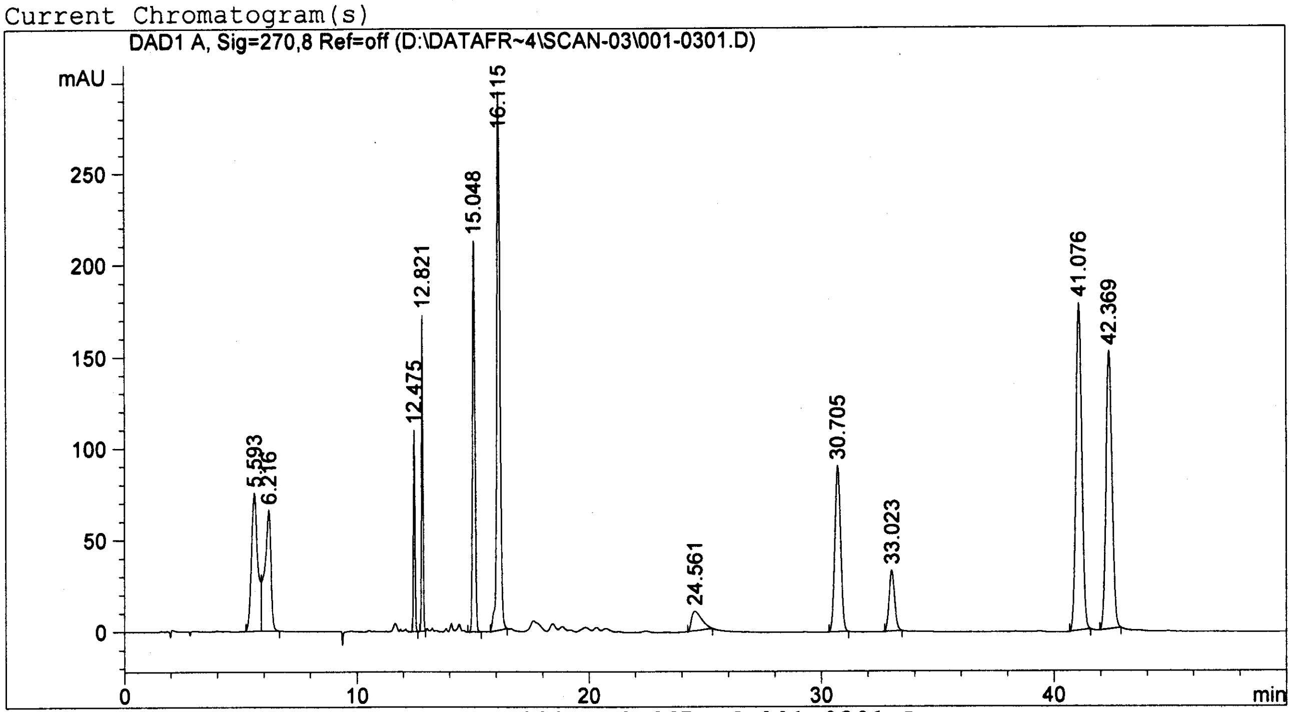

DAD1, 6.167 (196 mAU, -) Ref=5.220 & 6.667 of 001-0301.D

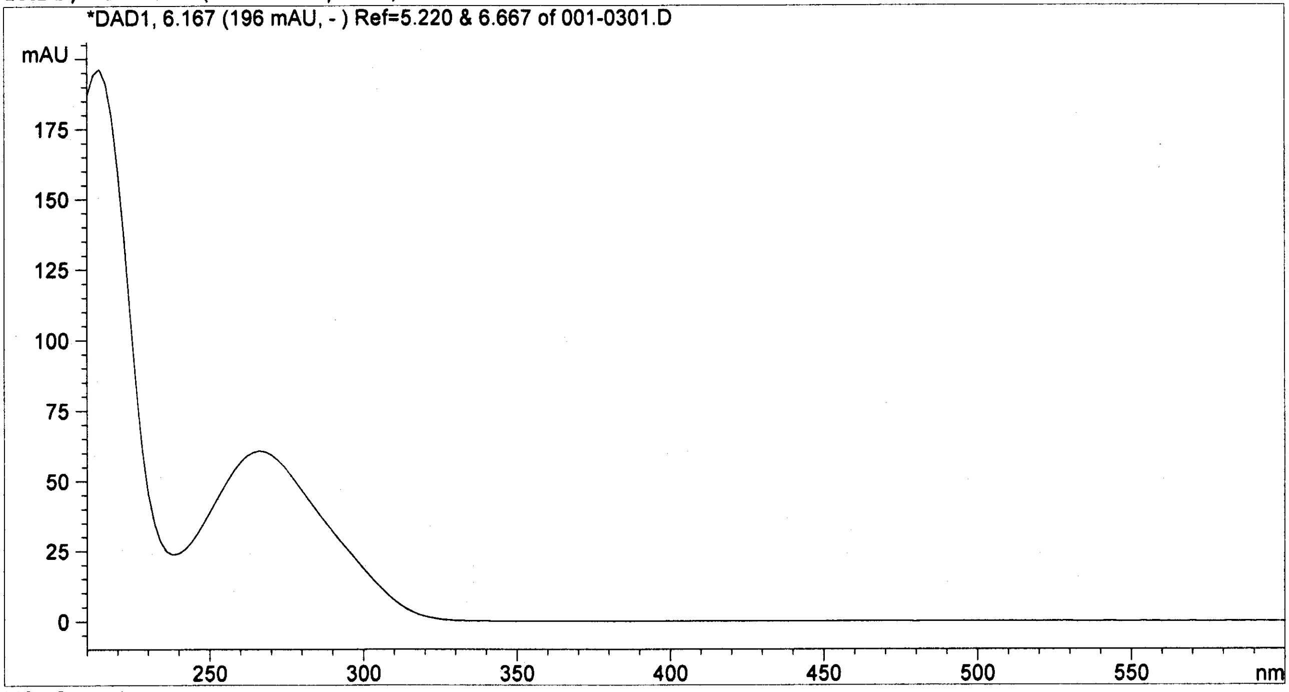

MS Spectrum

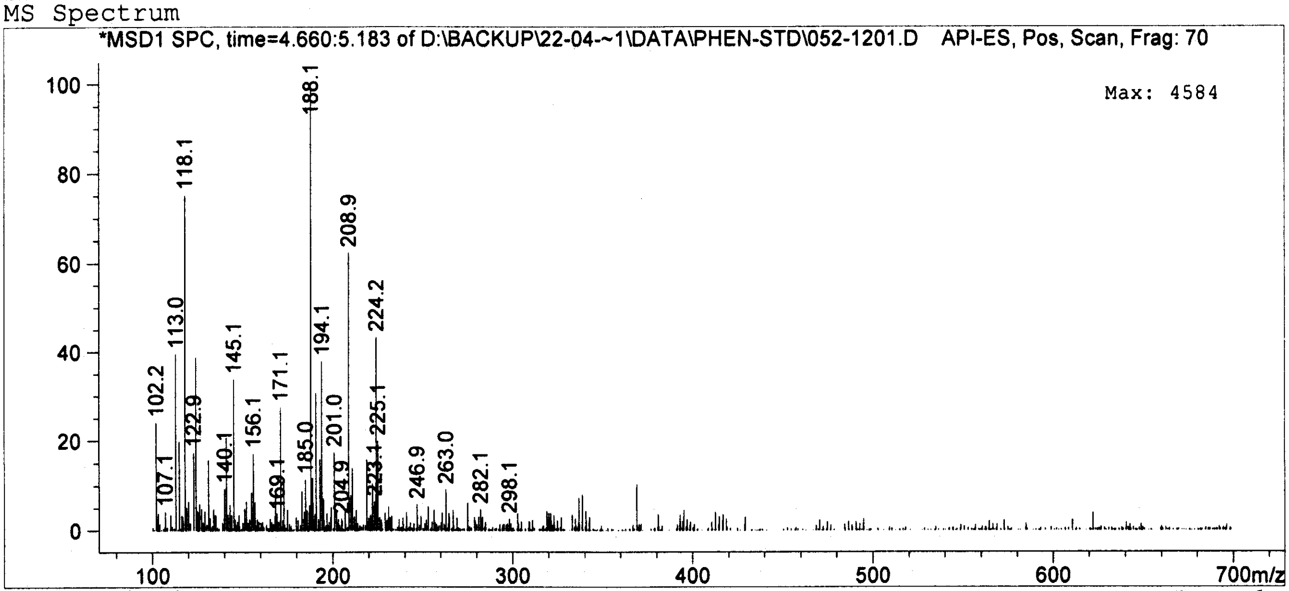

Catechin

Current Chromatogram(s)

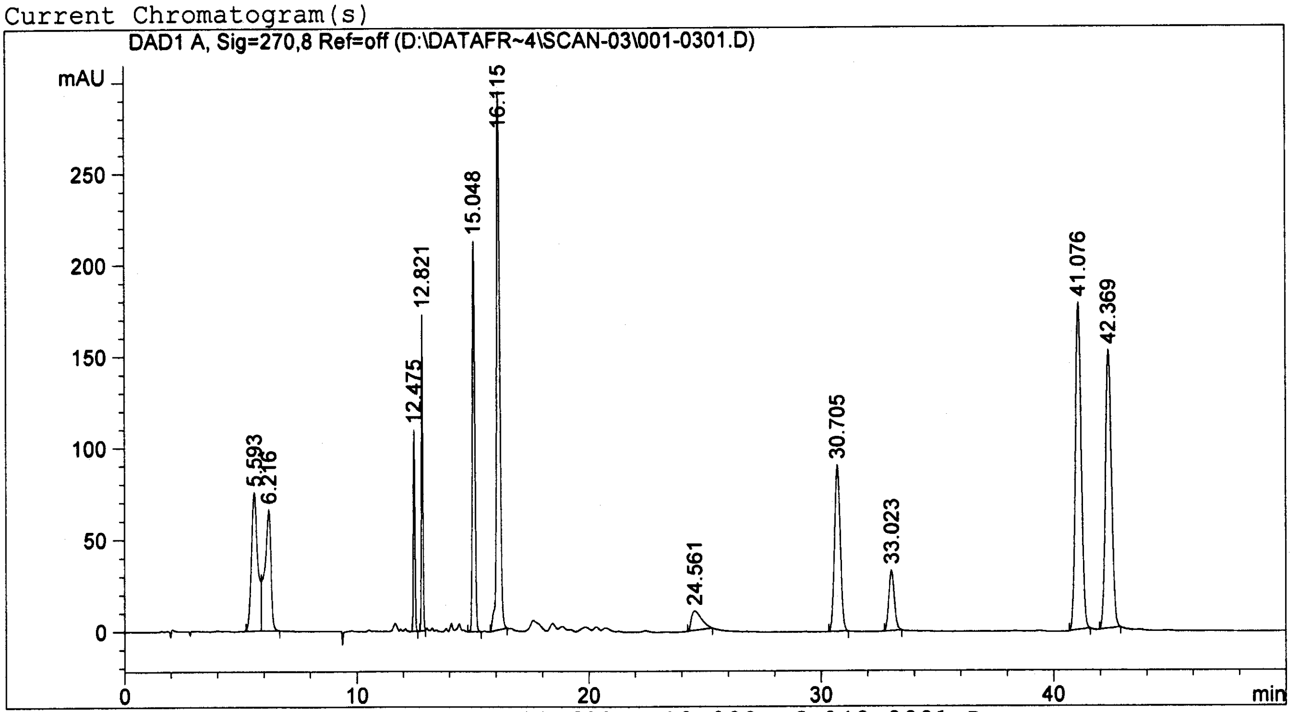

DAD1, 11.857 (1940 mAU, -) Ref=11.690 & 12.090 of 049-0901.D

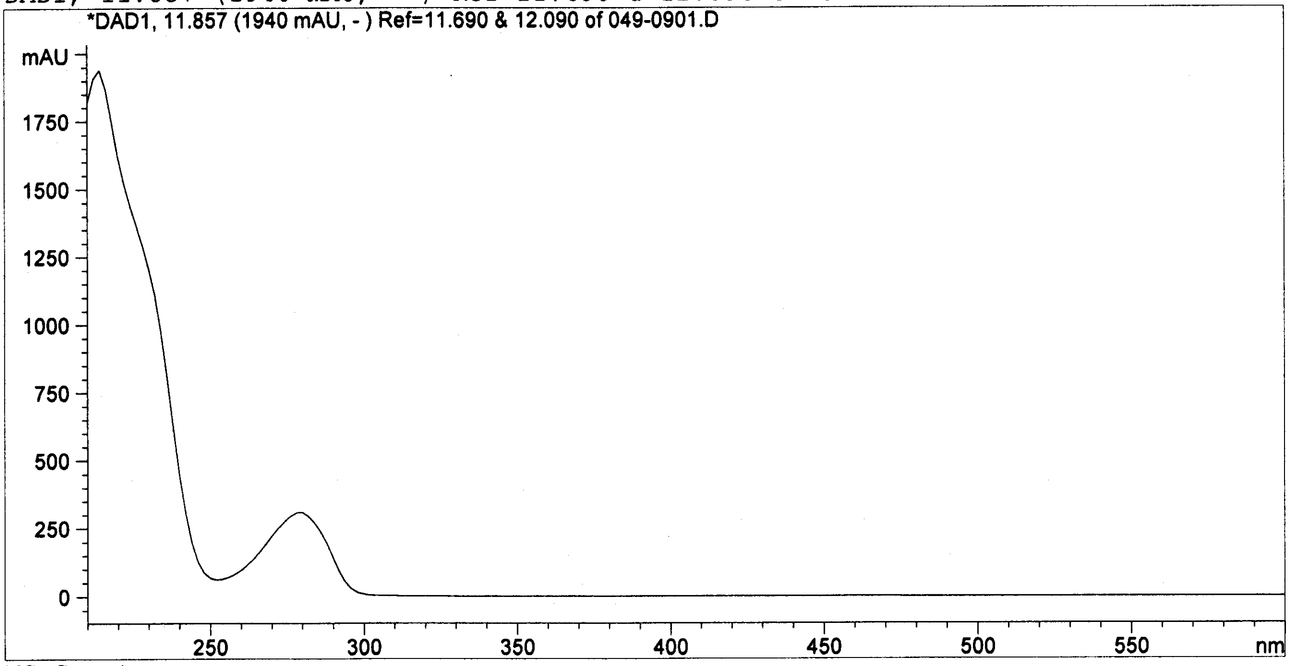

MS Spectrum

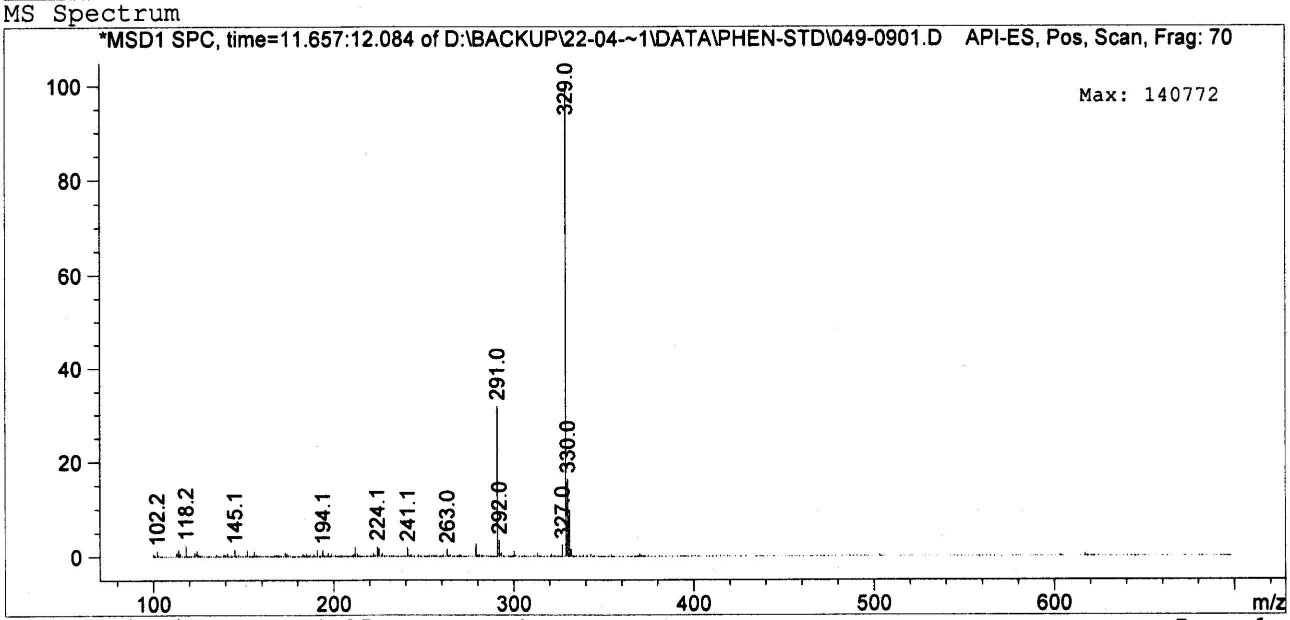

Print of all graphic windows

Parunit 2

Current Chromatogram(s)

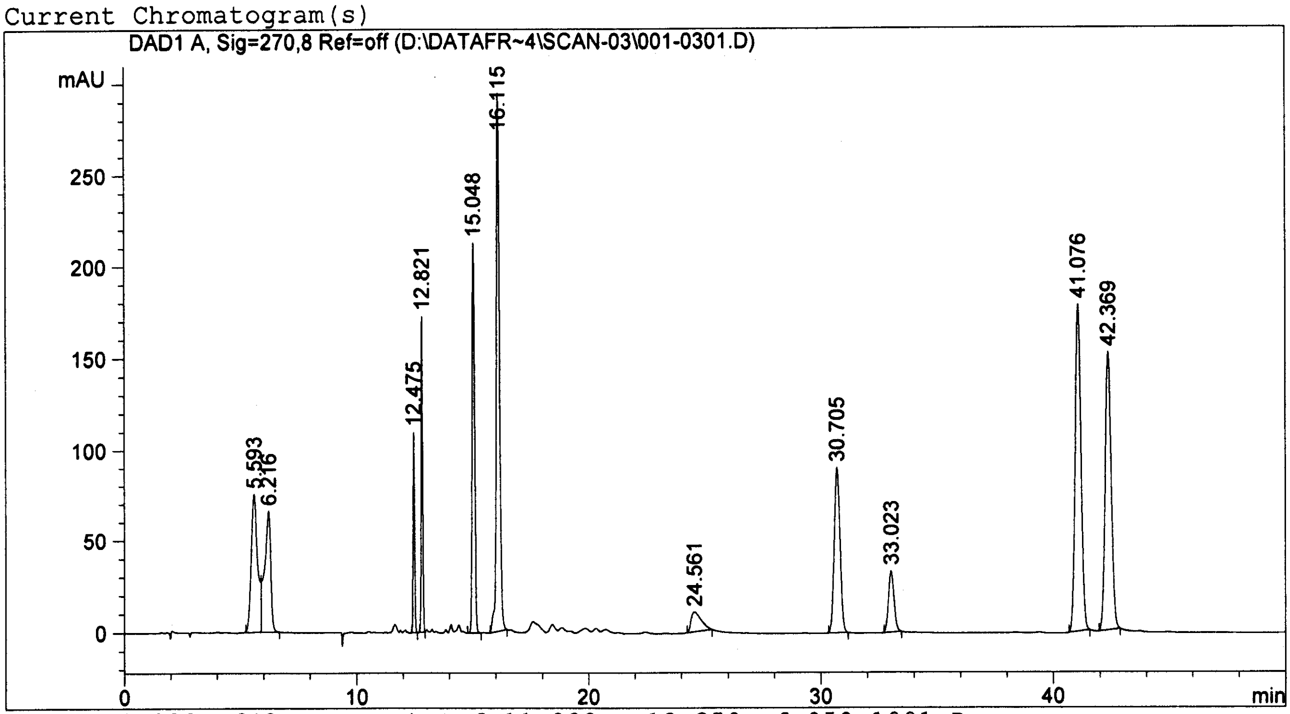

DAD1, 12.030 (642 mAU, -) Ref=11.823 & 12.350 of 050-1001.D

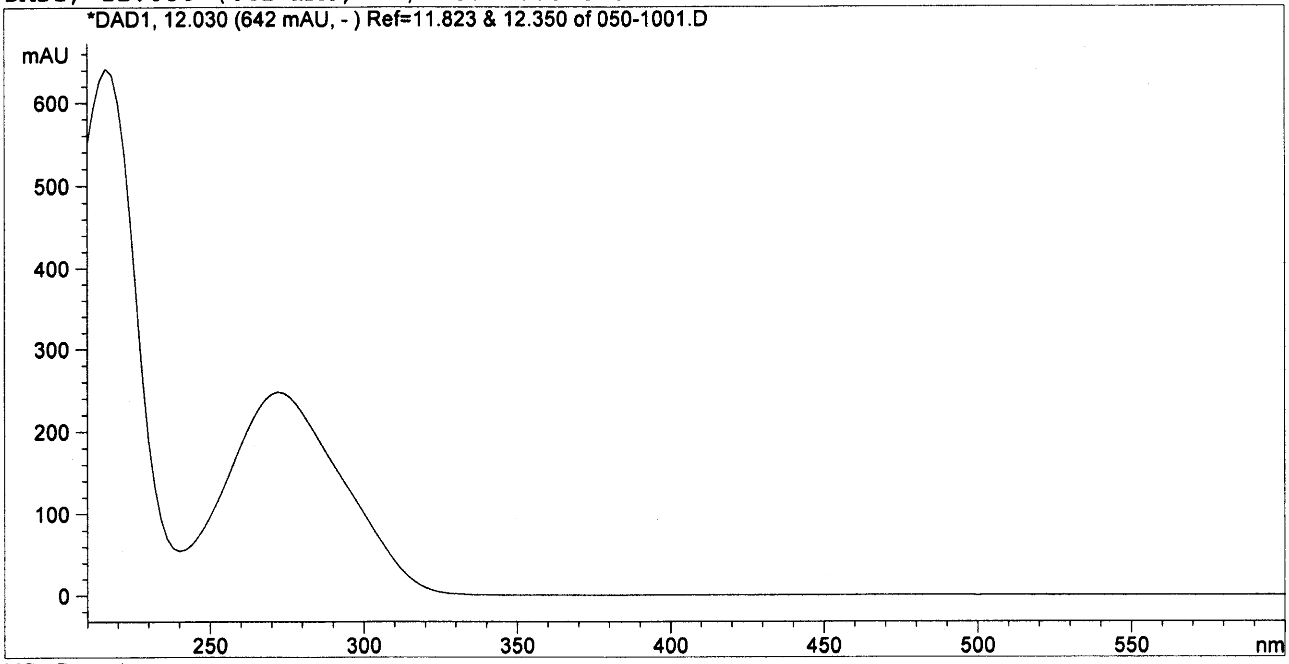

MS Spectrum

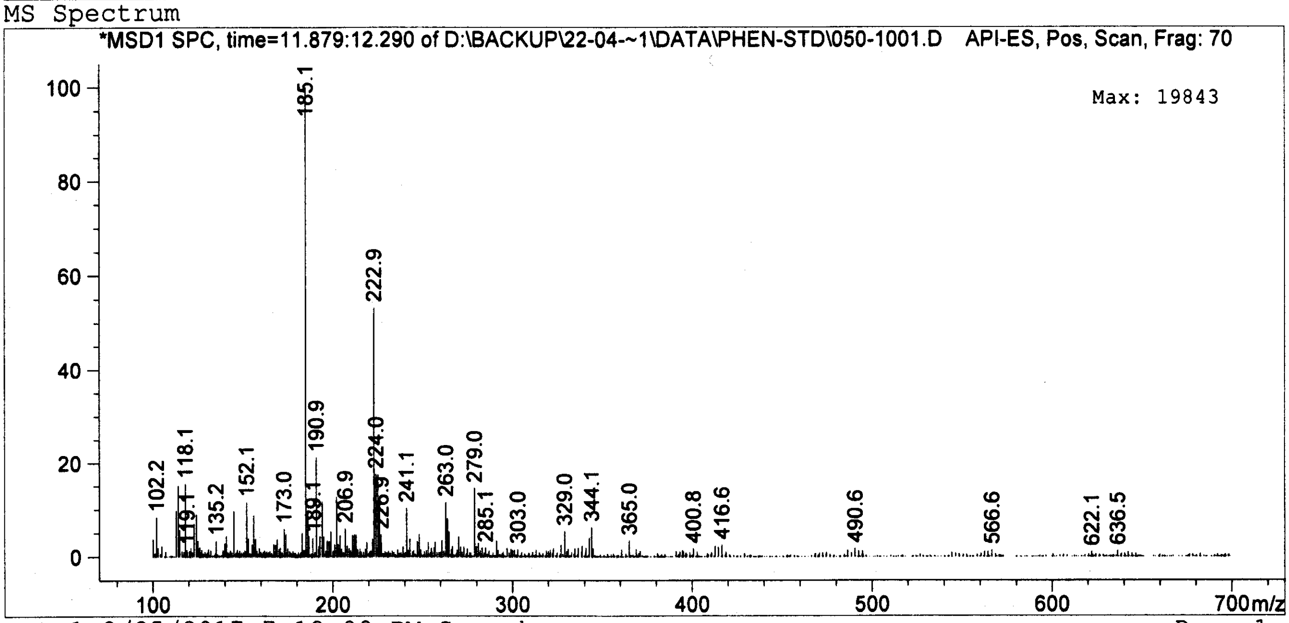

Instrument 1 2/25/2017 7:18:09 PM Somsak

Print of all graphic windows

Rutin

Current Chromatogram(s)

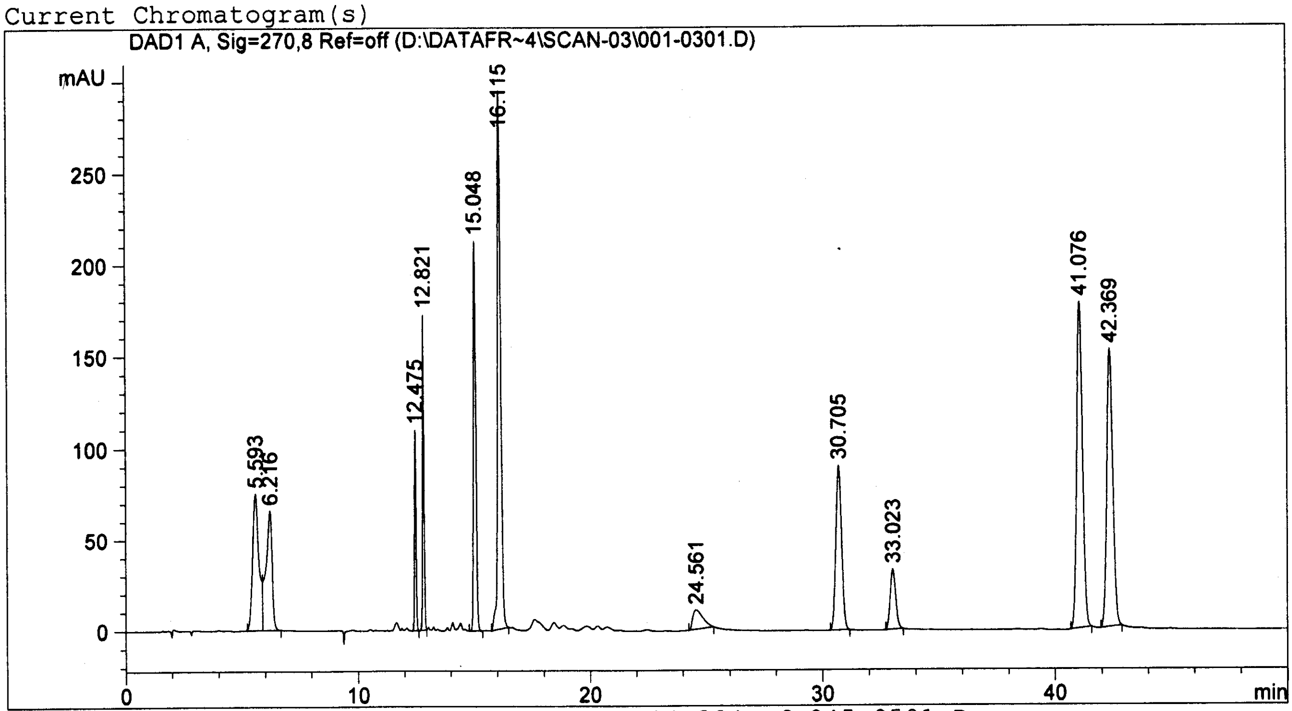

DAD1, 14.087 (522 mAU, -) Ref=13.754 & 14.394 of 045-0501.D

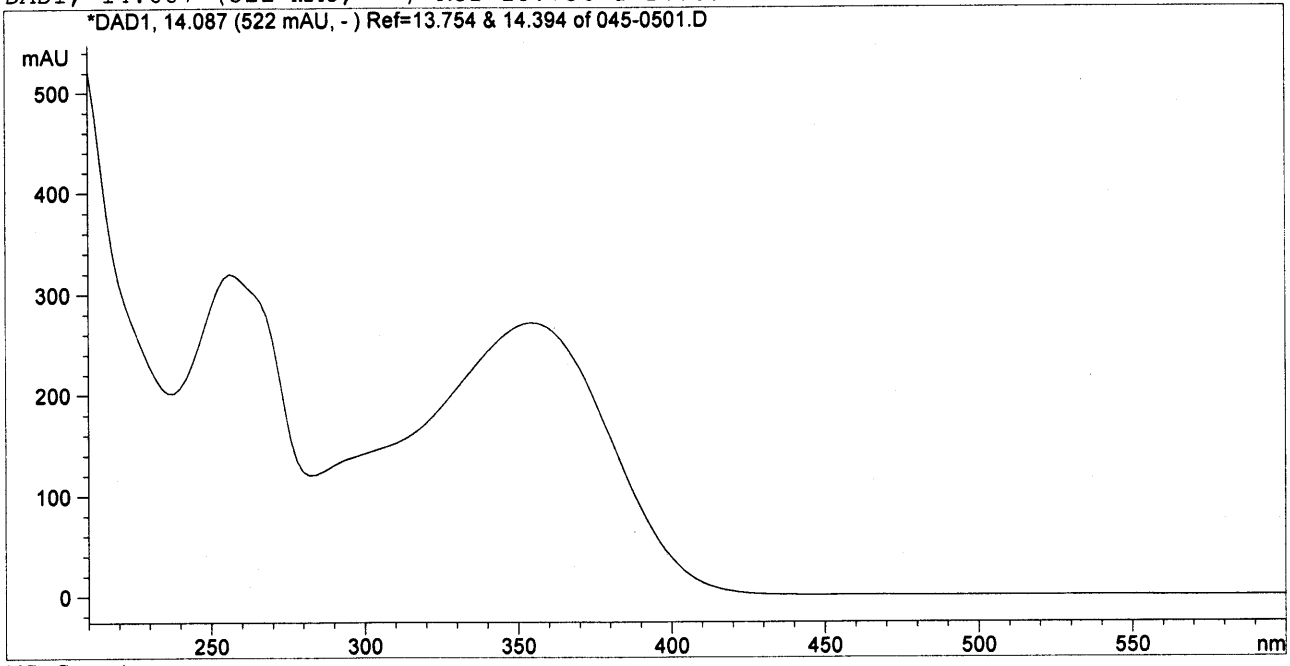

MS Spectrum

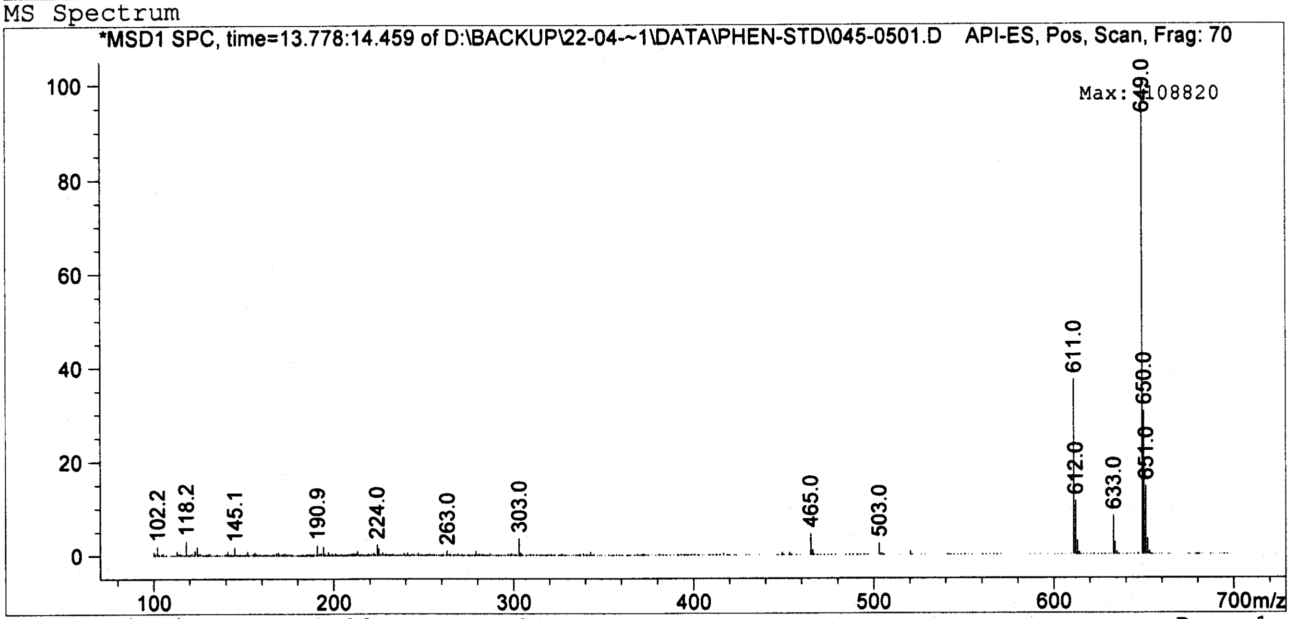

Print of all graphic windows

i-Quercetin

Current Chromatogram(s)

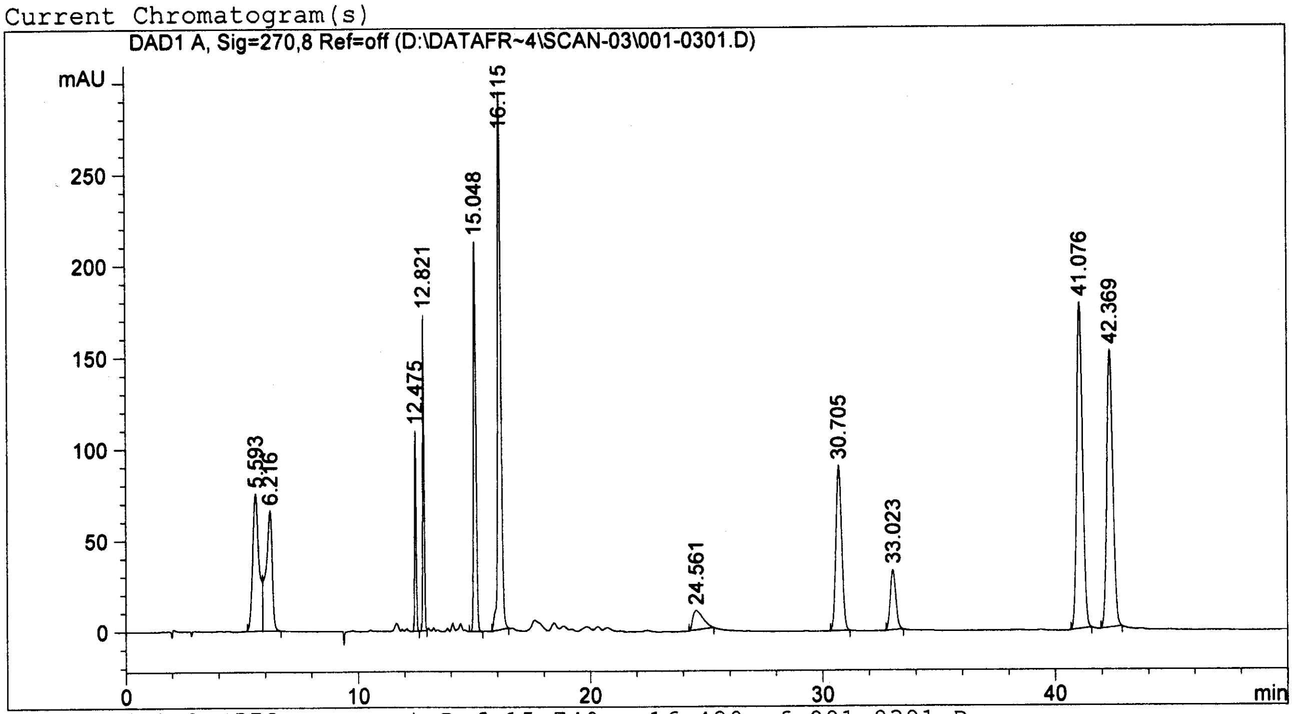

DAD1, 16.140 (573 mAU, -) Ref=15.740 & 16.480 of 001-0301.D

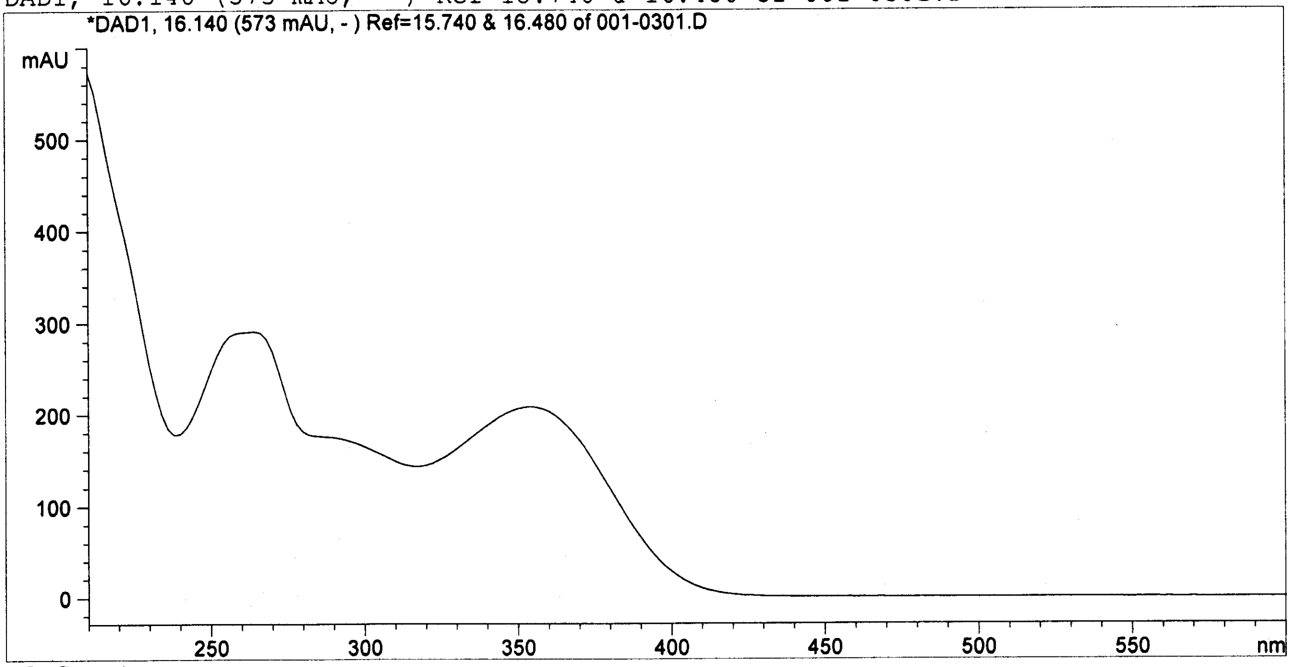

MS Spectrum

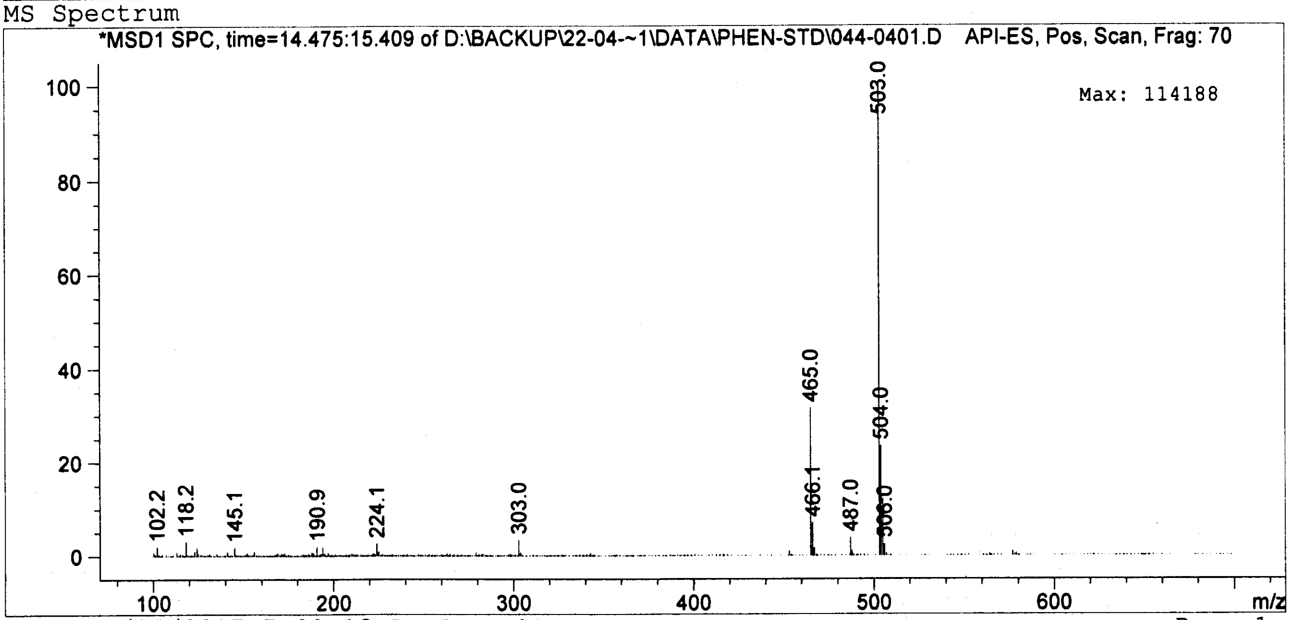

Print of all graphic windows

Hydroquinine

Current Chromatogram(s)

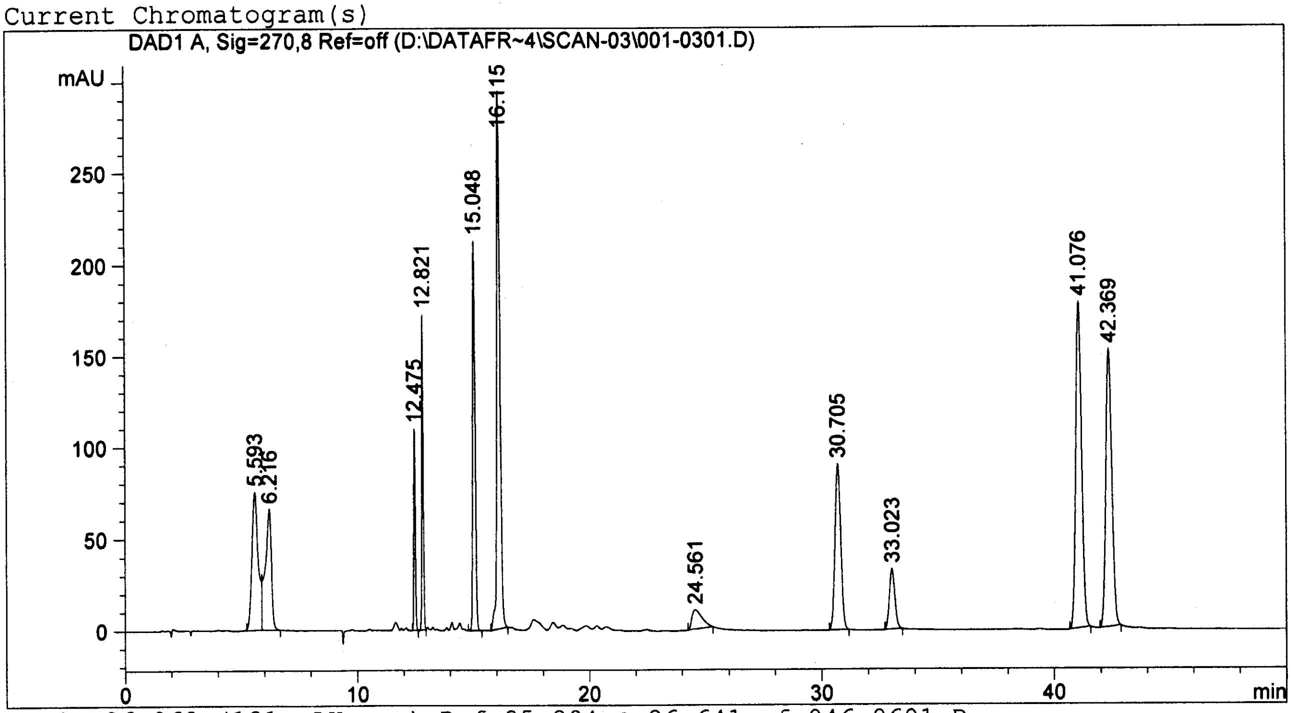

DAD1, 26.068 (121 mAU, -) Ref=25.294 & 26.641 of 046-0601.D

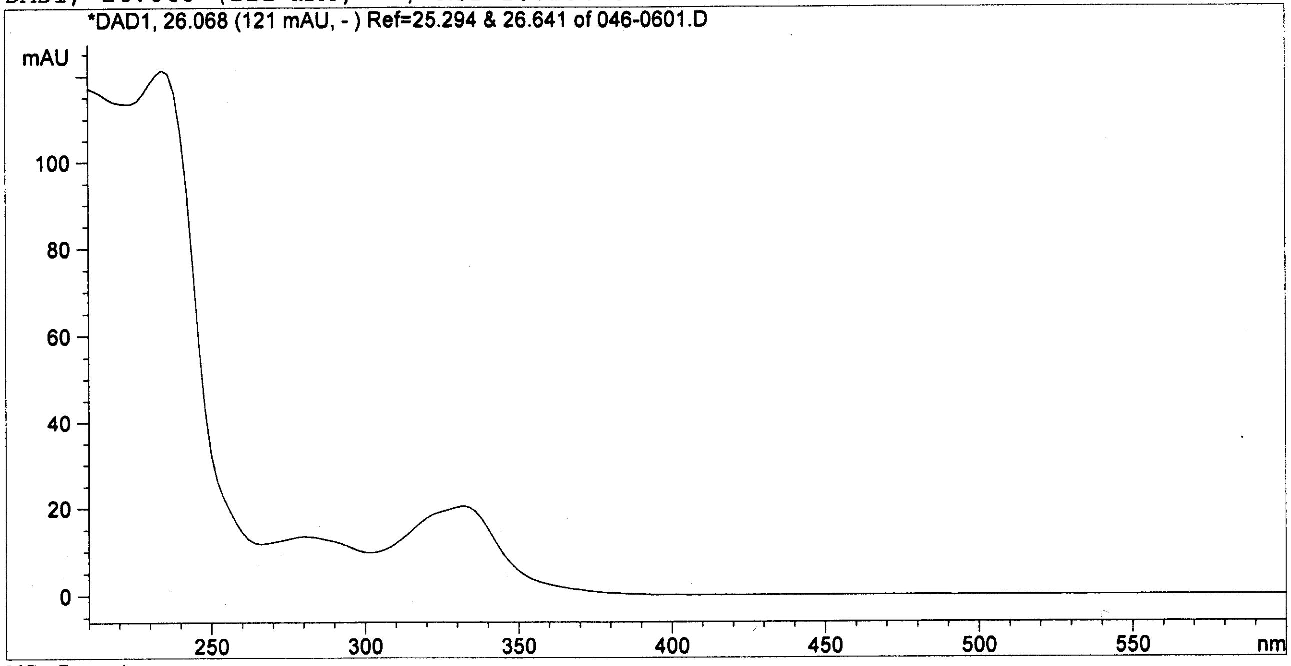

MS Spectrum

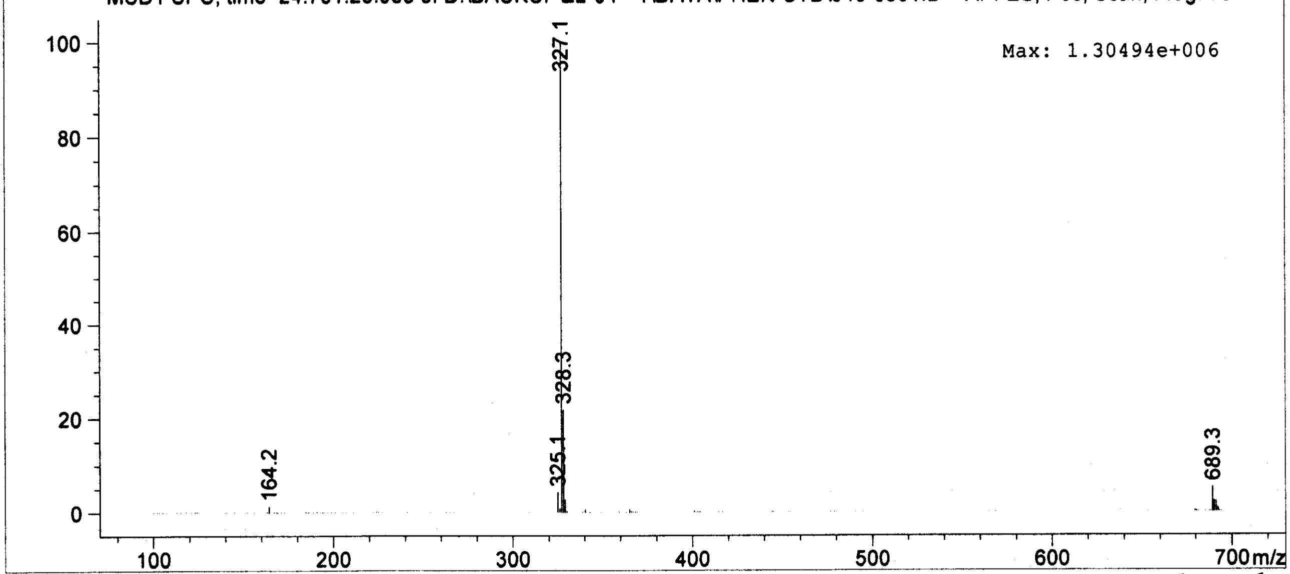

Print of all graphic windows

Eriodictyol.

Current Chromatogram(s)

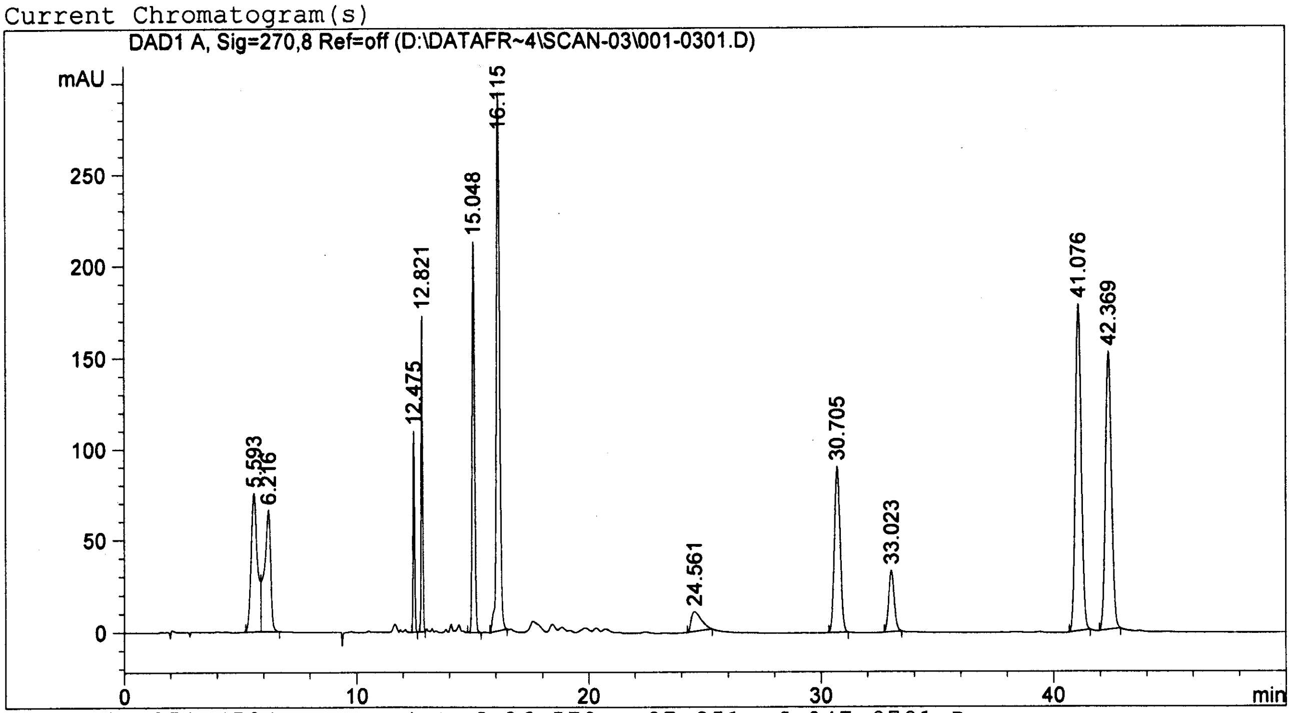

DAD1, 27.271 (524 mAU, -) Ref=26.578 & 27.851 of 047-0701.D

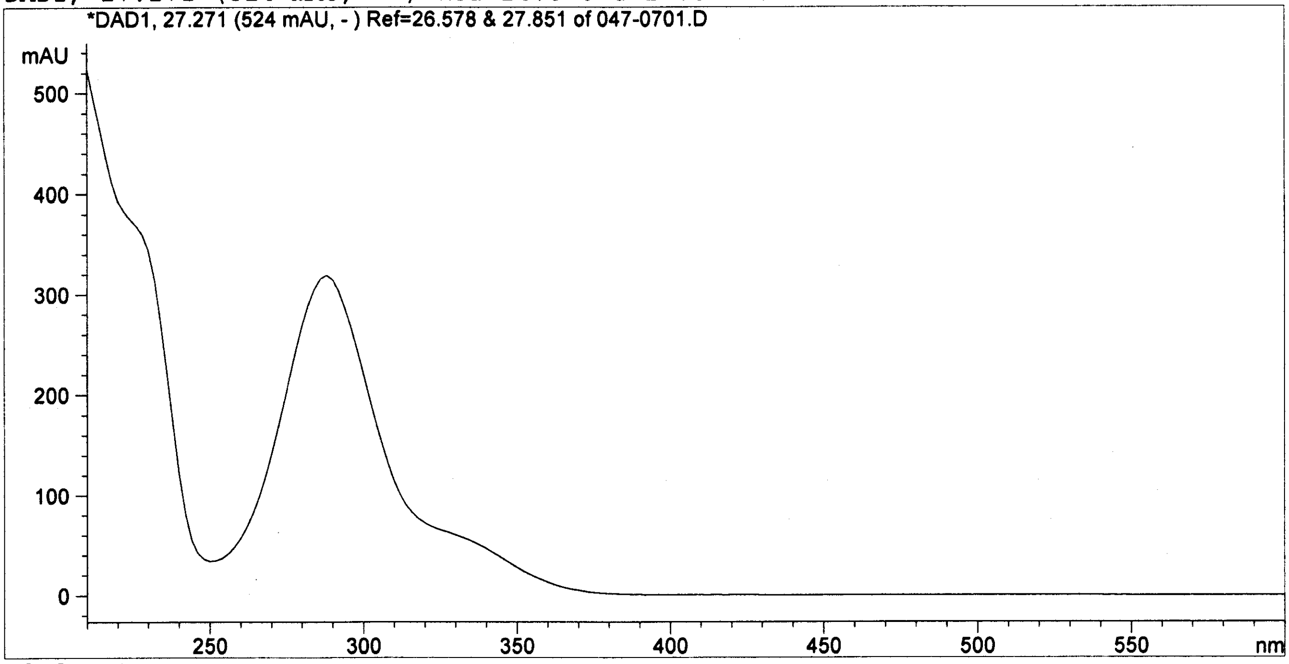

MS Spectrum

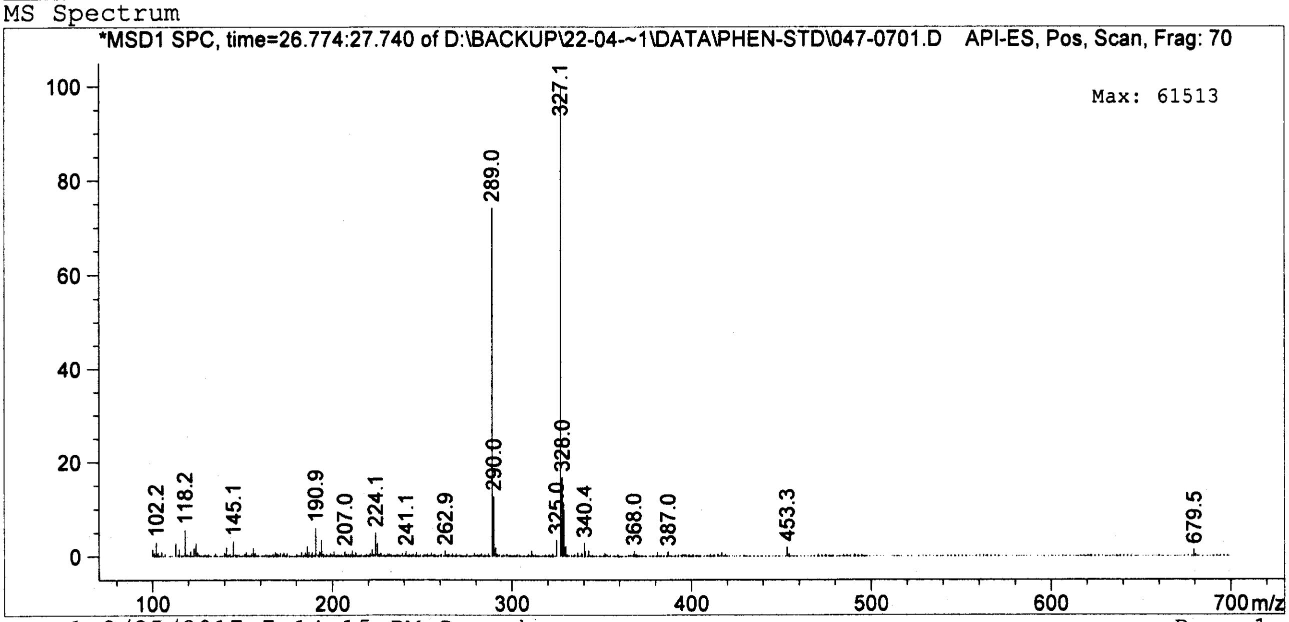

Instrument 1 2/25/2017 7:14:15 PM Somsak

Print of all graphic windows

Quercetin

Current Chromatogram(s)

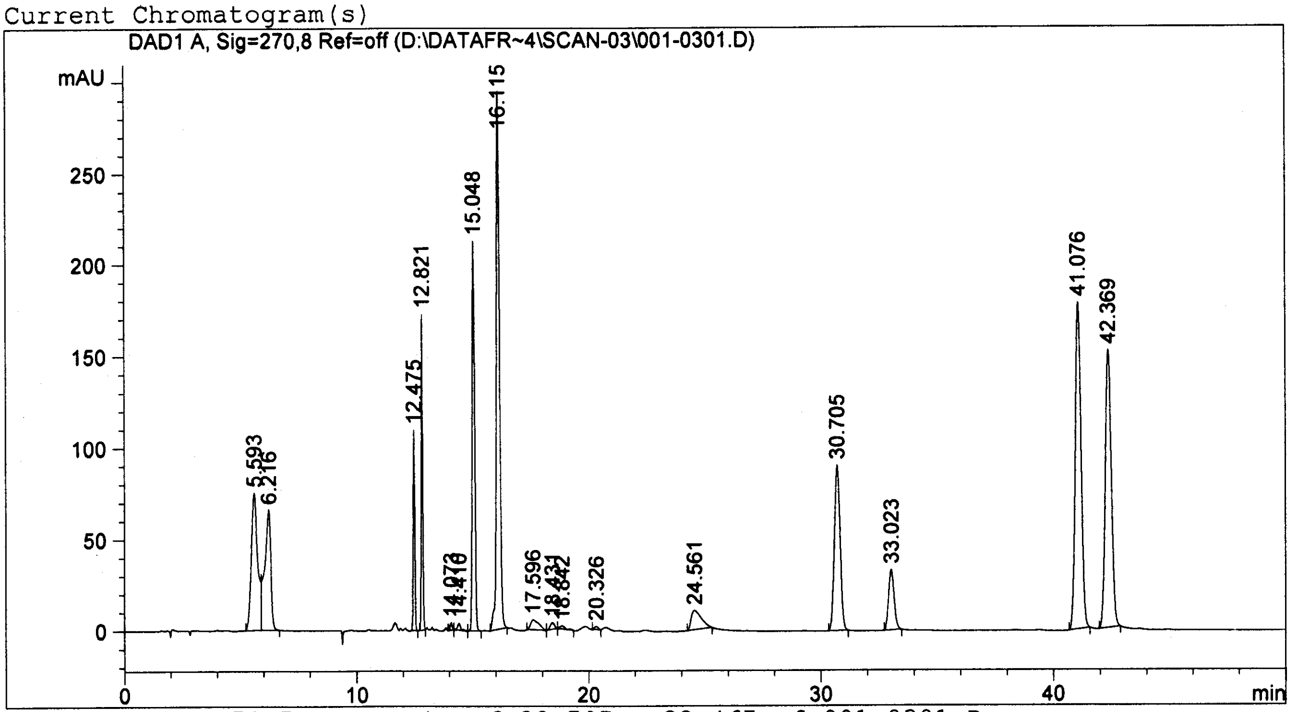

DAD1, 33.047 (71.7 mAU, -) Ref=32.707 & 33.467 of 001-0301.D

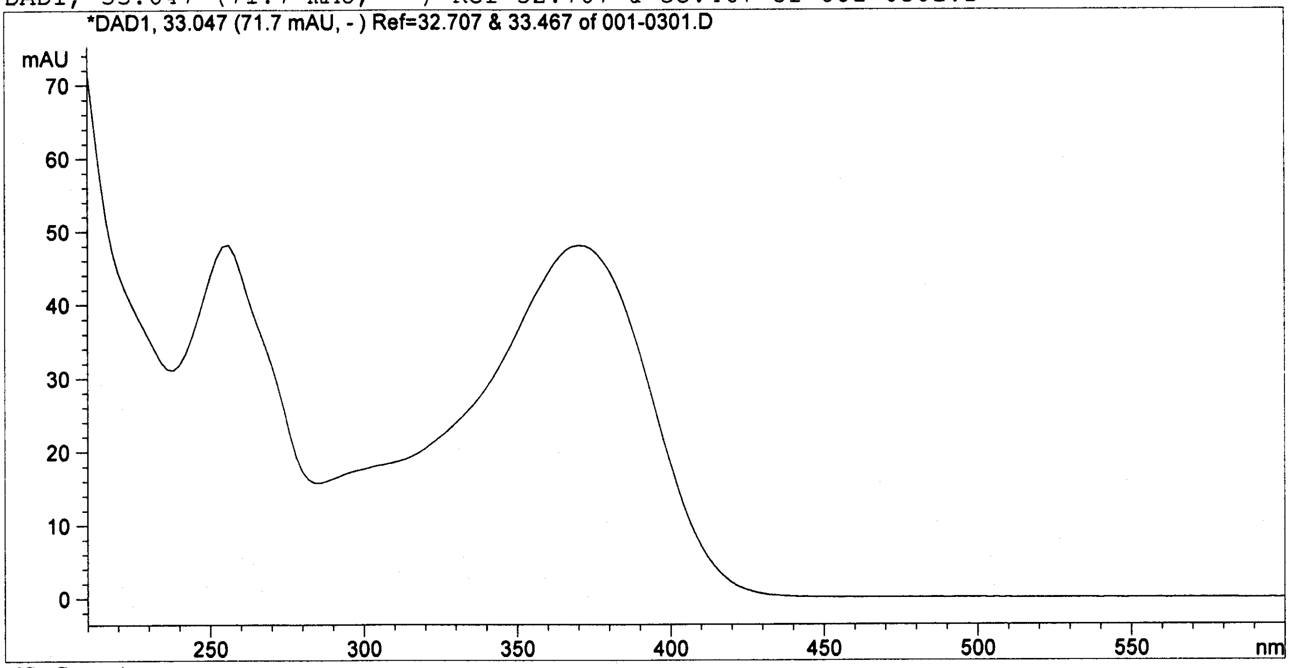

MS Spectrum

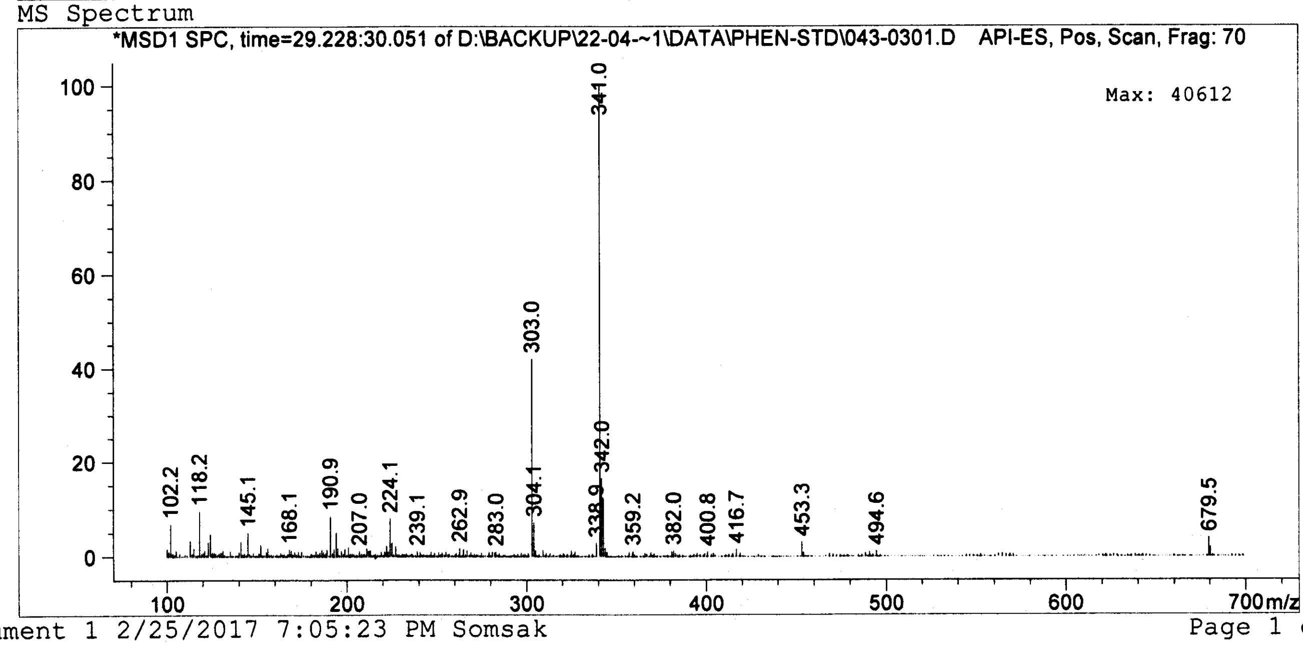

Apiginin

Current Chromatogram(s)

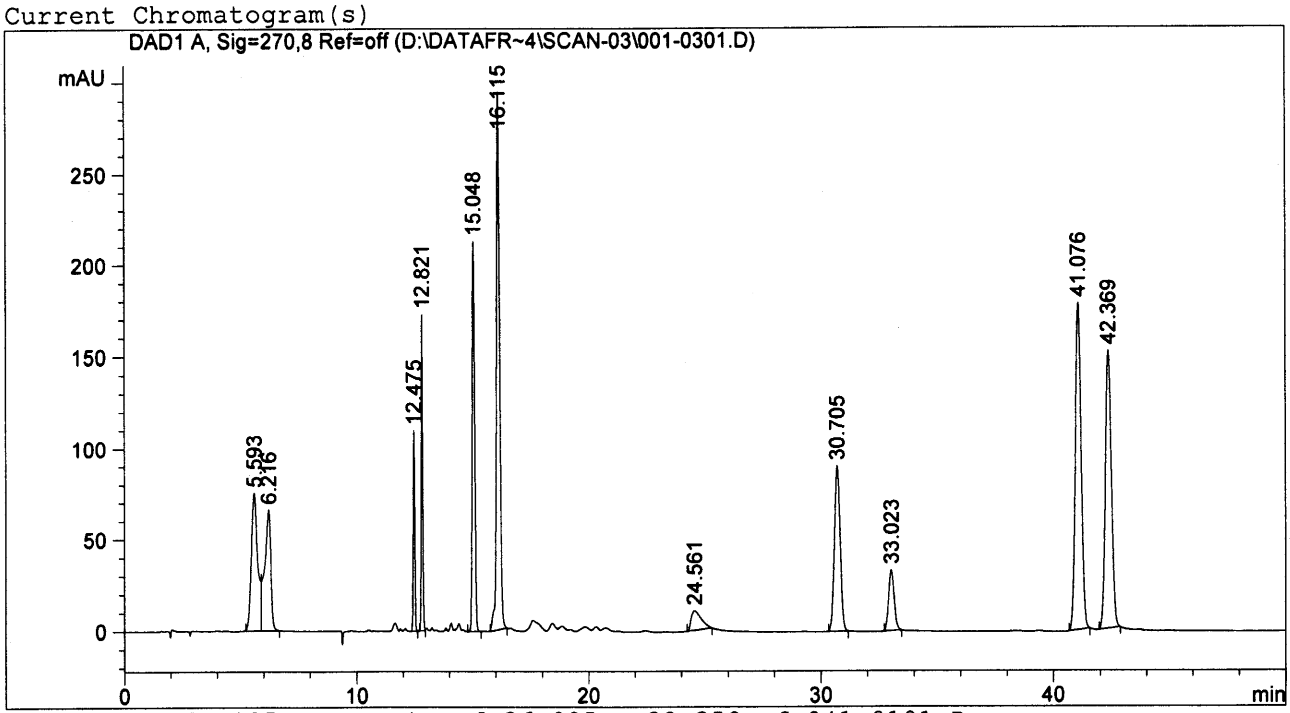

DAD1, 37.845 (105 mAU, -) Ref=36.985 & 38.359 of 041-0101.D

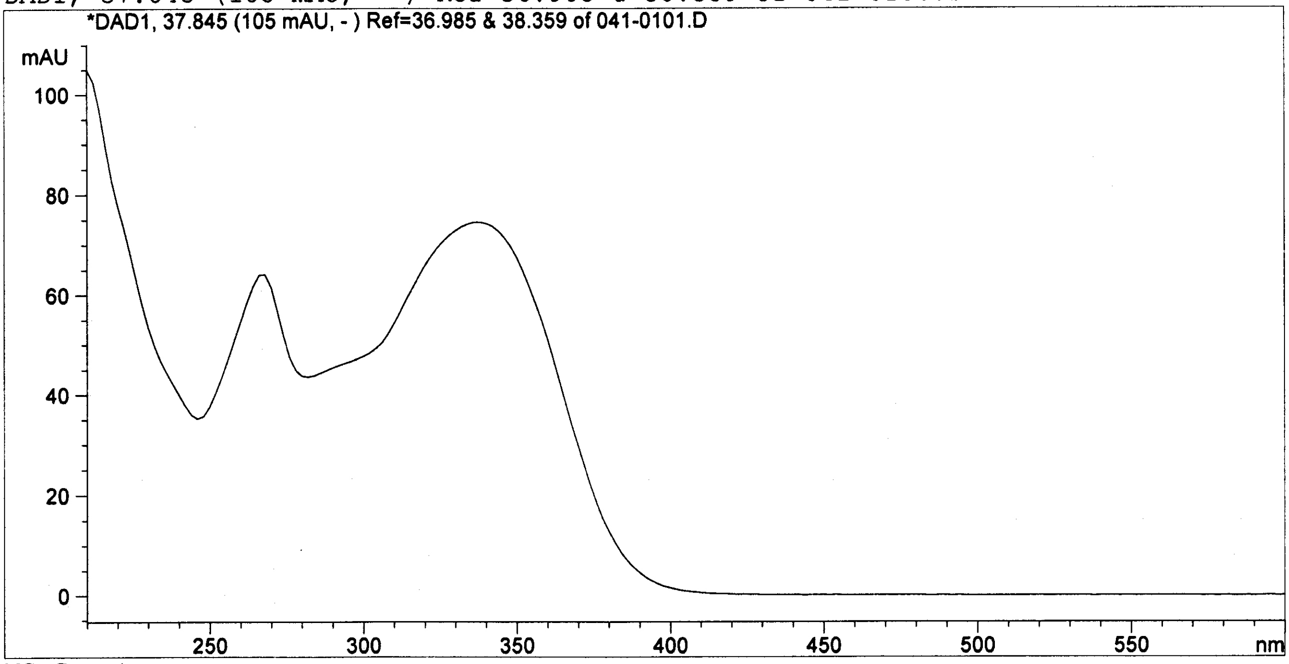

MS Spectrum

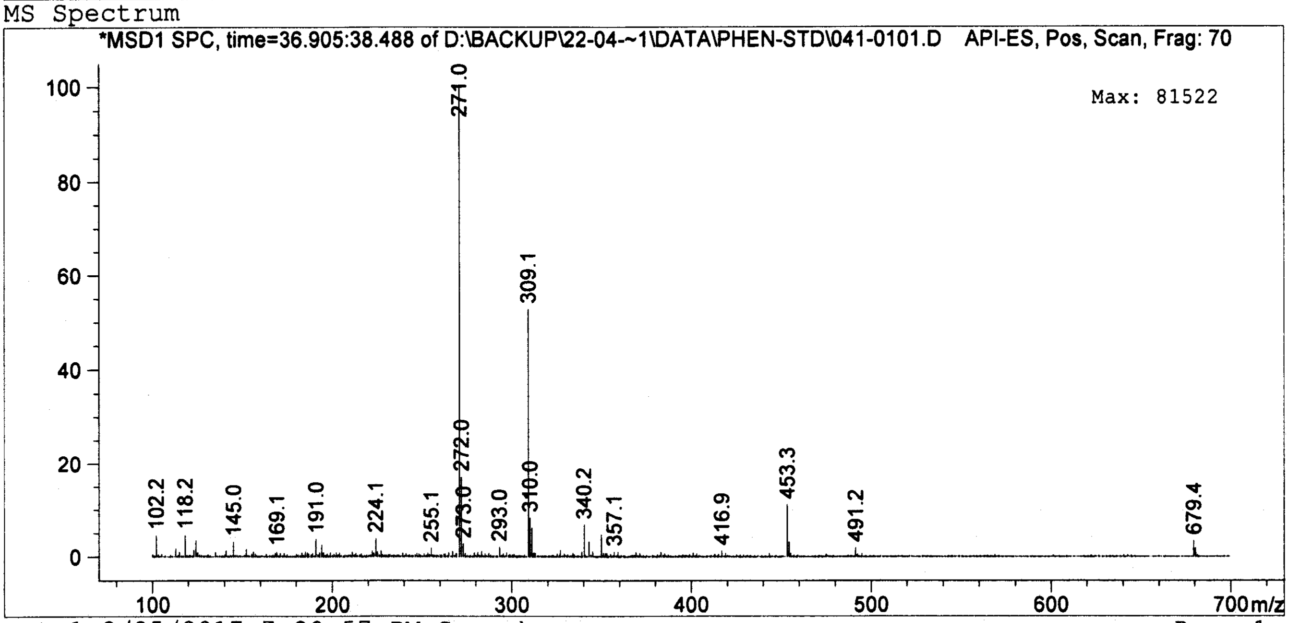

Kampherol

Current Chromatogram(s)

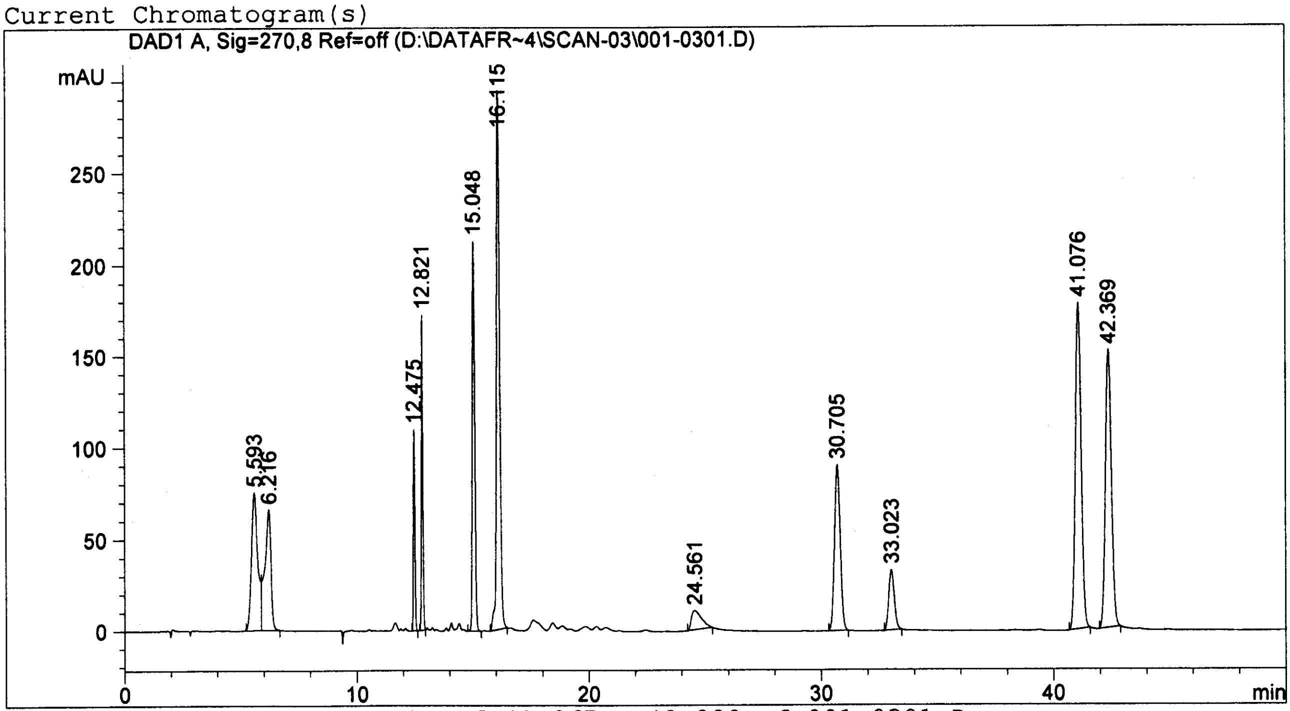

DAD1, 42.467 (154 mAU, -) Ref=41.967 & 42.880 of 001-0301.D

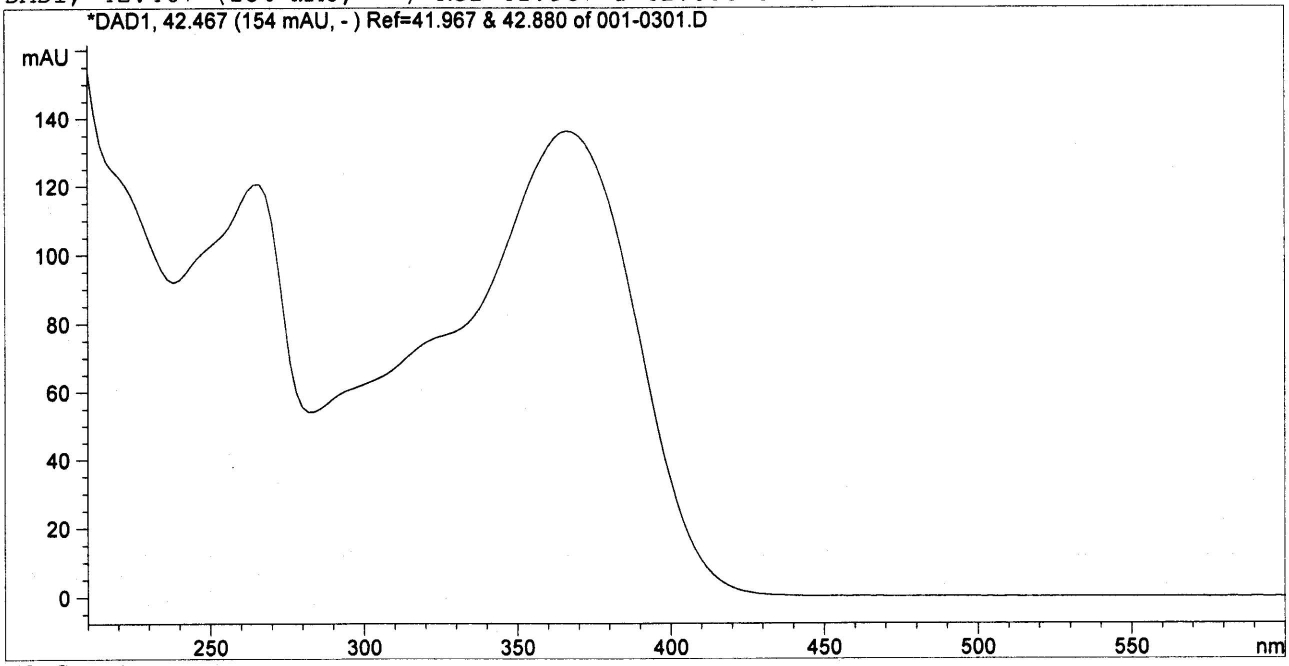

MS Spectrum

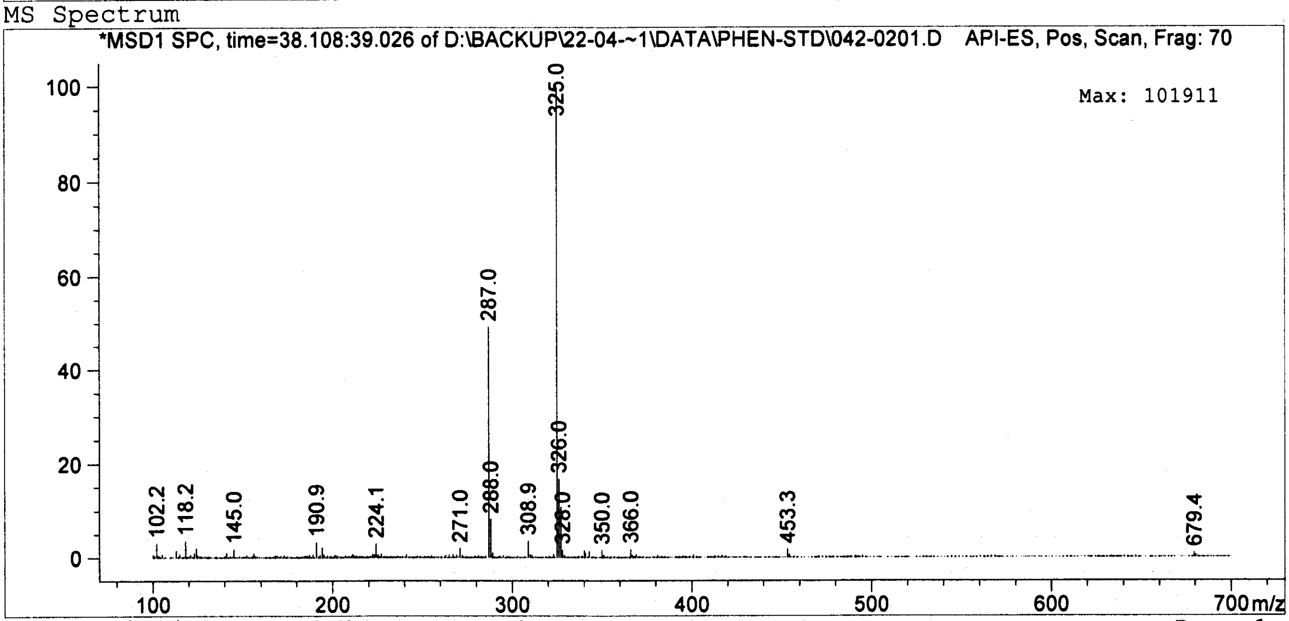

Supplement: Supplementary file 1 [file molecules-25-02474-s001.zip › molecules-804750-final-SM/Figure S3b LM MS for authentic phenolics.pdf]
