# Supplementary material for: Phytosterol, Lipid and Phenolic Composition, and Biological Activities of Guava Seed Oil
Source: Molecules. 2020 May 27;25(11):2474. doi: 10.3390/molecules25112474 (PMC7321134; doi:10.3390/molecules25112474)
Supplement: Supplementary file 1 [file molecules-25-02474-s001.zip › molecules-804750-final-SM/Figure S4 HPLC FLD of authentic FA (100 uM) E2.pdf]

==

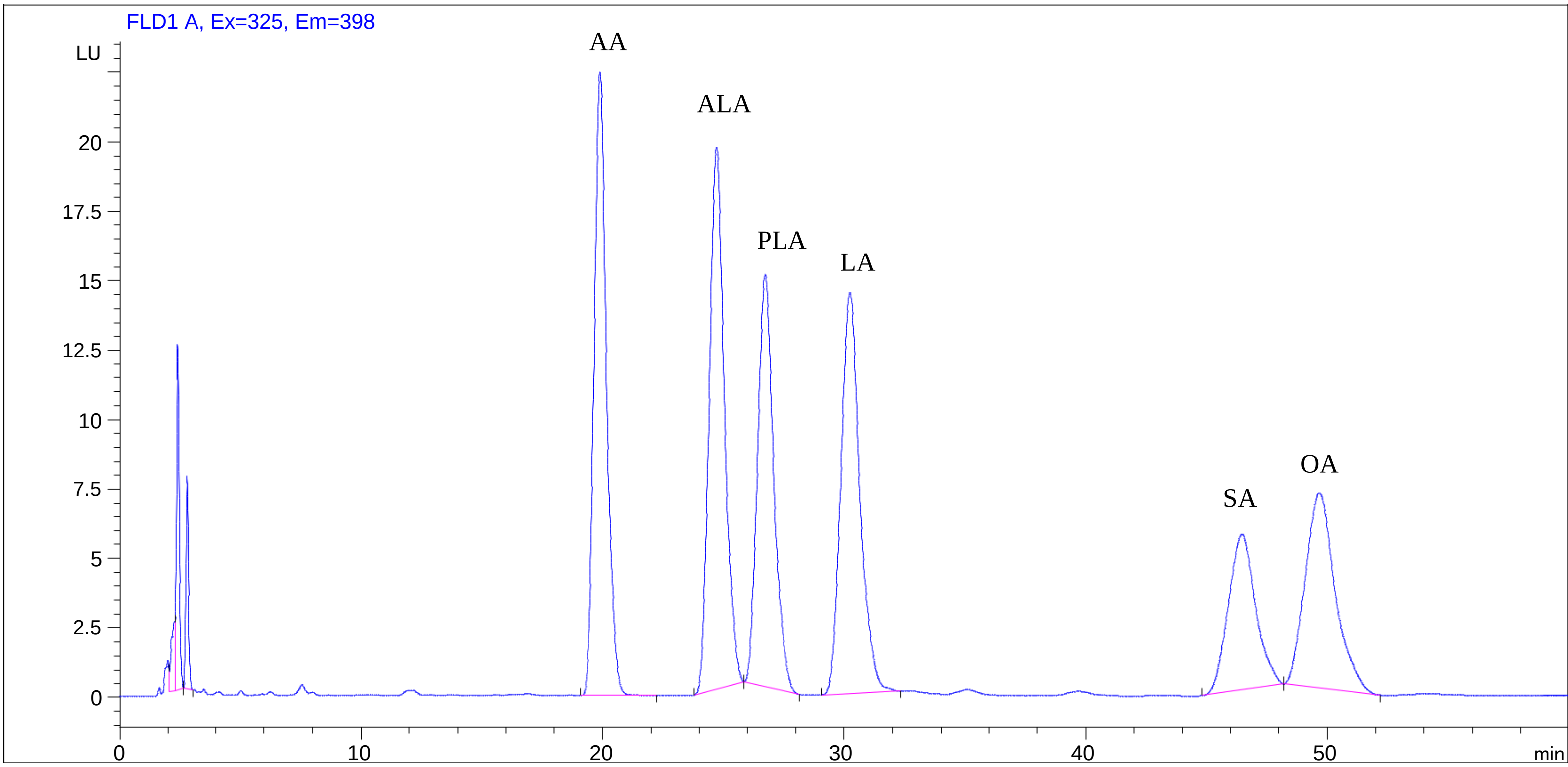

FLD1 A, Ex=325, Em=398
LU
AA
ALA
PLA
LA
SA
OA
0
2.5
5
7.5
10
12.5
15
17.5
20
0
10
20
30
40
50
min

Peak #	RetTime [min]	Type	Width [min]	Area [LU*s]	Height [LU]	Area %
7	30.239	BB	0.8289	784.64801	14.42857	17.9974
8	46.461	BB	1.1585	437.15863	5.60415	10.0271
9	49.658	BB	1.2552	603.83368	7.01252	13.8501
Totals :				4359.78820	106.38985	

*** End of Report ***
